# Synthesis of pyrrolo[2,1-*f*][1,2,4]triazin-4(3*H*)-ones: Rearrangement of pyrrolo[1,2-*d*][1,3,4]oxadiazines and regioselective intramolecular cyclization of 1,2-biscarbamoyl-substituted 1*H*-pyrroles

**DOI:** 10.3762/bjoc.12.168

**Published:** 2016-08-09

**Authors:** Kkonnip Son, Seong Jun Park

**Affiliations:** 1 Research Center for Medicinal Chemistry, Korea Research Institute of Chemical Technology (KRICT), 141 Gajeong-ro, Yuseong-gu, Daejeon 305-600, Korea; 2 Department of Chemistry, Sogang University, 35 Baekbeom-ro, Mapo-gu, Seoul 121-742, Korea

**Keywords:** intramolecular cyclization, pyrrolooxadiazines, pyrrolotriazinone, rearrangement

## Abstract

Pyrrolo[2,1-*f*][1,2,4]triazin-4(3*H*)-ones **12** have been easily prepared via nucleophile-induced rearrangement of pyrrolooxadiazines **11** and regioselective intramolecular cyclization of 1,2-biscarbamoyl-substituted 1*H*-pyrroles **10**. In this work, we demonstrated that the described synthetic approaches can be considered to be more facile and practical than previously reported procedures.

## Introduction

**Figure 1:**
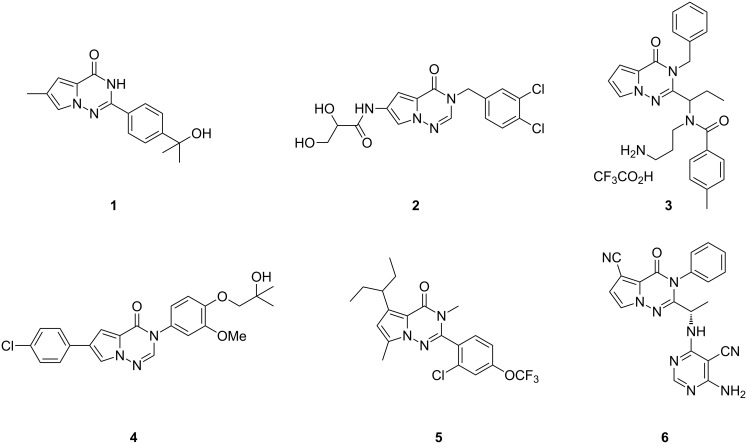
Bioactive pyrrolo[2,1-*f*][1,2,4]triazin-4(3*H*)-ones [1–12].

Pyrrolo[2,1-*f*][1,2,4]triazin-4(3*H*)-ones have been considered to be biologically active compounds. For example, these nitrogen-containing heterocycles have shown intriguing activities as tankyrase inhibitors **1** [1–2], stearoyl CoA desaturase inhibitors **2** [3], Eg5 inhibitors **3** [4–5], melanin-concentrating hormone receptor (MCH)-R1 antagonists **4** [6], and CRF1 receptor antagonists **5** [7–8] (Figure 1). Notably, many patent applications have described pyrrolotriazinones as phosphoinositide 3-kinase (PI3K) inhibitors **6** [9–12].

**Figure 2:**
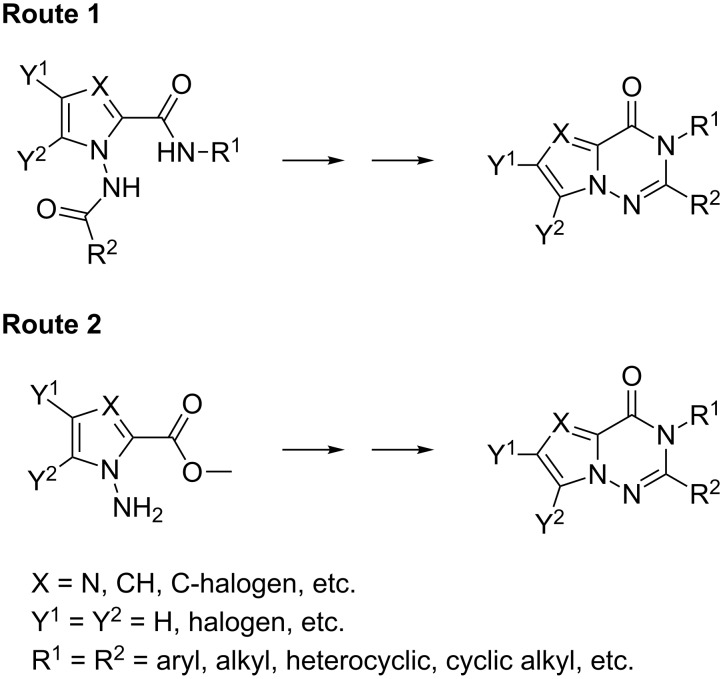
General synthetic routes to pyrrolotriazinones [3–6,8–9,11].

These skeletons are the key intermediates for the synthesis of pyrrolo[2,1-*f*][1,2,4]triazines, which have been shown to have outstanding biological activities [13–17]. Consequently, many research groups have developed synthetic approaches; two main synthetic routes involve *N*-imine intermediates and could be considered for the preparation of pyrrolotriazinones (Figure 2). Based on the reported cyclization methods, however, the reactions require high temperatures and long reaction times (generally overnight) to obtain the desired products [1–12]. For example, these cyclization methods involve procedures such as microwave-assisted heating with NaOMe [1] and H_2_N-Ar [6] at 150–160 °C, refluxing with HC(OEt)_3_ [3] and xylene [4–5,8], stirring at 100 °C in the presence of either NaOH or KOH [4,9], and heating with POCl_3_ [11] (Figure 2). It is reasonable to consider that these harsh conditions are required because it is difficult to form the *N*-imine structure and to subsequently perform intramolecular cyclization (Figure 2).

**Scheme 1:**
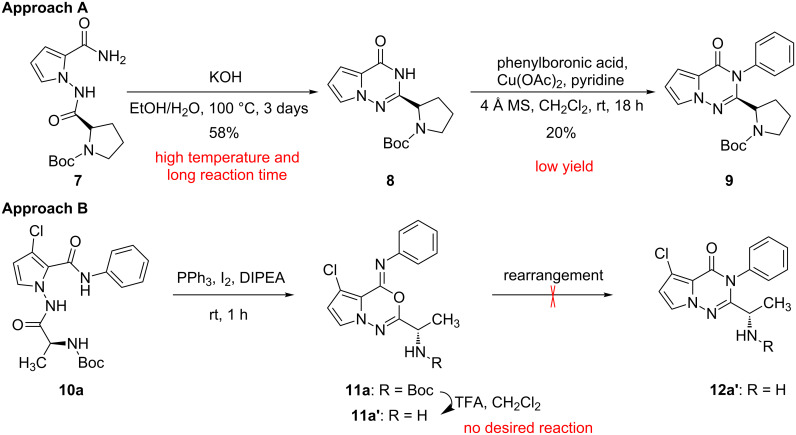
Synthesis of pyrrolotriazinones **9** and **12** [9–10,18].

In our efforts to discover drugs that are PI3K inhibitors, a Hutchison Medipharma patent caught our attention. They reported that pyrrolotriazinones showed excellent inhibitory activities against PI3K enzymes [9]. However, their synthetic method to prepare the target molecule **9** demonstrated a limited scope, and involved high temperature, long reaction time, and low yield (approach A, Scheme 1). Another synthetic approach, reported by researchers at Infinity Pharmaceuticals Inc., has been used to obtain triazinone **12a’** via rearrangement of oxadiazines **11a’** (approach B, Scheme 1) [10].

However, in our investigation of the reported rearrangement reaction, the desired product **12a’** was not accessed (approach B, Scheme 1). For the procedure using silica-gel column chromatography to afford triazinone **12a’** from the free amine-containing oxadiazine **11a’** [10], compound **11a’** was not present after the boc-deprotection reaction because of its instability in the acidic conditions.

Based on the literature and the attempts reported herein, it should be highlighted that limitations exist for the preparation of the desired compounds **12**. Due to these difficulties, we have investigated the synthesis of pyrrolotriazinones **12** by using a more convenient and facile approach than those that have been previously reported in the literature [9–12].

## Results and Discussion

**Scheme 2:**
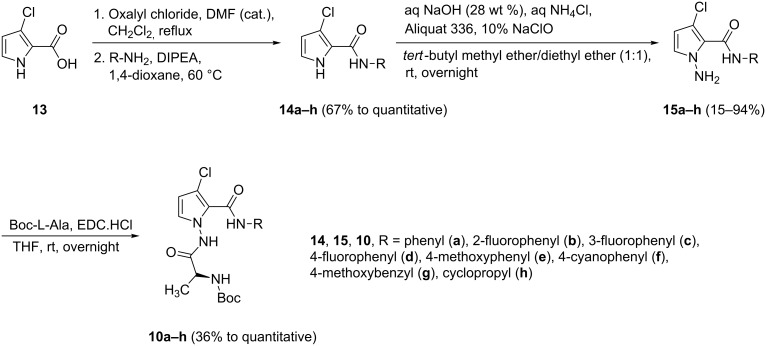
Synthesis of aminopyrrolocarbamate **10**.

Our studies started with the synthesis of aminopyrrolocarbamate **10**. The preparation of compound **10**, which is illustrated in Scheme 2, involved chlorination of 3-chloro-1*H*-pyrrole-2-carboxylic acid (**13**) using the Vilsmeier reagent [9], followed by further amination to produce 1*H*-pyrrole-2-carboxamide **14** in good to excellent yield [9]. A reaction mixture of **14** with NaOH, NH_4_Cl, and NaClO led to the formation of the *N*-aminopyrrole **15** [11]. The addition of the NH_2_^+^ to the nitrogen of pyrrole **14** by using the NaOH/NH_4_Cl/NaClO system [11] can be considered as a more practical method than others, such as those that use NH_2_Cl and HOSA [19]. In contrast to other substituents, 2-fluorophenyl and 4-cyanophenyl groups caused low yields (**15b**: 15%, **15f**: 31%). The *N*-aminopyrroles **15** were then reacted with EDC·HCl and Boc-L-alanine in THF to give the desired aminopyrrolocarbamate **10** in good to excellent yield [9].

To synthesize the desired pyrrolotriazinones **12** regioselectively we initially considered the work of Mazurkiewicz [20–21]. He reported that a mixture of 4*H*-3,1-benzoxazines (*O*-imidoylation products) and 4-quinazolones (*N*-imidoylation products) could be obtained after heating *N*-acylanthranilamides in CH_2_Cl_2_ under reflux with PPh_3_Br_2_ in the absence of triethylamine. In his research, it was proved that HCl or HBr influenced the rearrangement of benzoxazines to quinazolones. Importantly, triethylamine was considered to be an HBr captor [20–21].

With regard to Mazurkiewicz’s work, the effect of Et_3_N on intramolecular cyclization was explored, and the acid-assisted rearrangement was also evaluated.

As shown in Table 1, although all of the obtained yields were influenced by the amount of Et_3_N, the attempt to synthesize compound **12a** directly by optimizing the amount of base was not successful. For example, no reaction was observed in the absence Et_3_N (entry 1, Table 1). When excess amounts of base were used, compounds **11a** and **12a** were only obtained in low yields (40% combined yield, entry 3, Table 1). Alternatively, when 2.5 equivalents of Et_3_N were used, the two regioisomers **11a** and **12a** were obtained in an excellent overall yield of 87% (entry 2, Table 1). In addition, the ratio of **11a** to **12a** was not significantly affected by reaction times and temperatures (entries 4–6, Table 1).

**Table 1:** The studies on various reaction conditions.

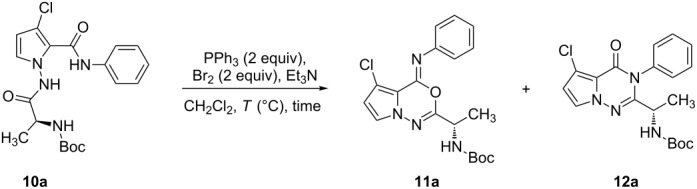			

Entry	Et_3_N (equiv)	Reaction conditions	Yield [%]^a ^**11a** (**12a**)

1	None	0 °C, 1 h → rt, 0.5 h	–^b^ (–^b^)
2	2.5	0 °C, 5 min	53 (34)
3	10	0 °C, 5 min	11 (29)
4	5	0 °C, 5 min	68 (22)
5	5	0 °C, 1 h → rt, 6 h	63 (20)
6	5	reflux, 10 min	59 (16)

^a^After column chromatography, ^b^not obtained.

Although initial attempts to synthesize pyrrolotriazinone **12a** regioselectively were not successful, it should be highlighted that the regioisomers oxadiazine **11a** and triazinone **12a** could be easily prepared under very mild conditions (0 °C for 5 min), whereas only the oxadiazine **11a** had been obtained in other reported procedures [10,12].

The acid-promoted rearrangement of oxadiazine **11** to triazinone **12** was also examined. However, the trial reaction was not successful because compound **11** did not tolerate acidic conditions.

Because of this result, the rearrangement reaction of pyrrolooxadiazine **11a** to pyrrolotriazinone **12a** was explored (Table 2). For nucleophile-induced cyclization, pyrrolidine, Li(Me_3_AlSPh) [22], NaSMe, and NaOMe were assessed. Attempting the Mazurciewitcz–Ganesan procedure [23], using pyrrolidine as a nucleophile, was not successful (entry 1, Table 2). In the cases of Li[Me_3_AlSPh], NaSMe, and NaOMe, the triazinone **12a** was readily obtained after the nucleophilic-addition/ring-closure reaction (entries 2–5, Table 2). For example, similar to benzoxazine [22], treatment of **11a** and **11d** with lithium trimethyl(phenylsulfido)aluminate Li(Me_3_AlSPh) provided the desired pyrrolotriazinone, **12a** and **12d**, in excellent yields and with retention of enantiomeric excesses (ee) (entries 2 and 3, Table 2). Interestingly, the rearrangement of oxadiazine **11a** with sodium thiomethoxide led to the desired compound **12a** (92% yield, entry 4, Table 2), and retention of ee was observed. With sodium methoxide, the ee was not retained, but the desired product **12a** was obtained in excellent yield (entry 5, Table 2). Notably, it has proven that sulfur-based reagents such as Li(Me_3_AlSPh) and NaSMe are efficient for the nucleophile-induced cyclization.

**Table 2:** Rearrangement of pyrrolooxadiazine **11** to pyrrolotriazinone **12**.

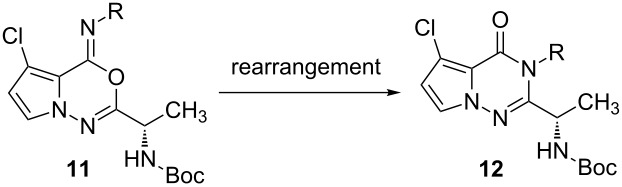					

Entry	R	Substrate/Product	Rearrangement conditions	Yield [%]^a^	ee [%]^b^

1	Ph	**11a**/**12a**	1. pyrrolidine, rt, 18 h 2. AcOH, CH_3_CN	–^c^	–^d^
2	Ph	**11a**/**12a**	Li(Me_3_AlSPh), THF, rt, 20 h	90	99
3	4-F-Ph	**11d**/**12d**	Li(Me_3_AlSPh), THF, rt, 20 h	69	99
4	Ph	**11a**/**12a**	NaSMe, THF/DMF, rt, 0.5 h	92	99
5	Ph	**11a**/**12a**	NaOMe, THF/DMF, rt, 3 h	85	88

^a^After column chromatography; ^b^the enantiomeric excess (ee) was determined after the amide coupling reaction of boc-deprotected **12** with moscher’s acid; ^c^not obtained; ^d^not determined.

Next, the effect of different halogens on the regioselectivity of the cyclization of **10** was investigated (Table 3). In general, the mixture of oxadiazines **11** and triazinones **12** was obtained in 45–98% overall yield. The results show that the regioselectivity is highly dependent on the halogen used. In particular, when PPh_3_Cl_2_ was used, triazinones **12** (*N*-imidoylation product) were more easily obtained than oxadiazines **11** (entries 1 and 4–6, Table 3). In the case of bromine, the *O*-imidoylation products **11** were preferred over the *N*-imidoylation products **12**, whereas for substrates with 2- and 3-fluorophenyl groups different results were obtained (entries 2 and 8–11, Table 3). Based on the literature results [9–12,22–25] and the reactions that are reported herein, the *O*-imidoylation product **11** is more accessible than the *N*-imidoylation product **12** when PPh_3_-Br_2_/I_2_-Et_3_N/DIPEA systems are applied (entries 2, 3, 10 and 11, Table 3).

**Table 3:** Investigation of regioselectivity.

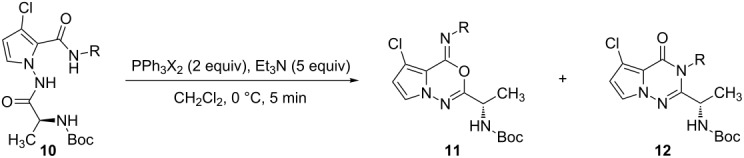				

Entry	R	X_2_	Products	Yield [%]^a ^**11** (**12**)

1	phenyl	Cl	**11a** (**12a**)	10 (87)
2	phenyl	Br	**11a** (**12a**)	63 (18)
3	phenyl	I	**11a** (**12a**)	81 (13)

4	3-fluorophenyl	Cl	**11c** (**12c**)	11 (72)
5	4-fluorophenyl	Cl	**11d** (**12d**)	15 (81)
6	4-methoxyphenyl	Cl	**11e** (**12e**)	20 (78)
7	4-cyanophenyl	Cl	**11f** (**12f**)	43 (41)

8	2-fluorophenyl	Br	**11b** (**12b**)	16 (29)
9	3-fluorophenyl	Br	**11c** (**12c**)	25 (68)
10	4-fluorophenyl	Br	**11d** (**12d**)	41 (19)
11	4-methoxyphenyl	Br	**11e** (**12e**)	70 (10)
12	4-cyanophenyl	Br	**11f** (**12f**)	–^b^ (–^b^)

13	4-methoxybenzyl	Br	(**12g**)	–^b^ (60)
14	cyclopropyl	Br	(**12h**)	–^b^ (66)

^a^After column chromatography; ^b^not obtained.

Interestingly, in the case of the 4-cyanophenyl group, it appeared that the different reaction patterns might be a result of the reagents PPh_3_Br_2_ and PPh_3_Cl_2_ (entries 7 and 12, Table 3). For alkyl substituents (4-methoxybenzyl and cyclopropyl, entries 13 and 14, Table 3), triazinones **12g** and **12h** were selectively prepared in over 60% yield. Based on these results, it is possible to consider that due to the presence of electron-donating groups, such as alkyl substituents, only the *N*-imidoylation products **12g**, and **12h** were formed.

**Figure 3:**
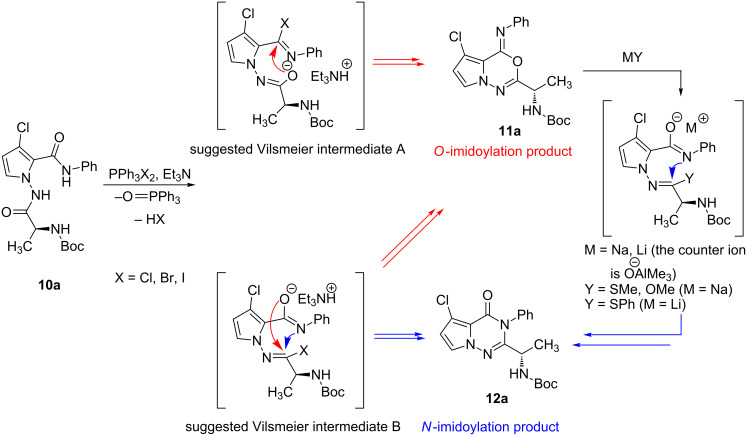
Probable mechanism for the synthesis of triazinone **12a**.

It is possible to propose a reaction mechanism after considering our studies and the literature results (Figure 3) [20–28]. For example, it is not reasonable to consider Mazurkiewicz’s acid-promoted rearrangement [20–21], because oxadiazine is not stable under acidic conditions. In the case of the rearrangement of **11a** to **12a**, the mechanism of the nucleophile-induced cyclization is proposed after considering Hart’s research on the synthesis of fumiquinazolines [22]. It was shown that the nucleophilicity of the *N*-acylnitrenium ion was increased when the oxygen ion was stabilized by counter ions such as lithium and sodium. For the intramolecular cyclization step, it was shown that the regioselectivity depends on the halogen source (Br/Cl) and neighboring groups of the *N*-acylnitrenium ions (electron-withdrawing aryl and -donating alkyl substitutents). This is highlighted by the observation that the *N*-imidoylation product (triazinone) **12** was preferentially obtained when a chlorine-halogen source and electron-donating alkyl groups were used. While further studies are required, we suggest the intermediates are *N*-acylnitrenium ions [26] and halogen-imine structures (the Vilsmeier type) [27–28].

Because oxadiazines **11** and triazinones **12** are non-crystalline, their exact structures were assigned by NMR spectroscopy (^1^H and ^13^C). With the literature results alone [9–12] the identity of the regioisomers could not be accurately confirmed; therefore, the NMR studies were required. As shown in Table 4, different NOEs were observed for compounds **11** and **12**.

**Table 4:** NOE analysis of representative examples (**11a**/**11d** and **12a**/**12d**).

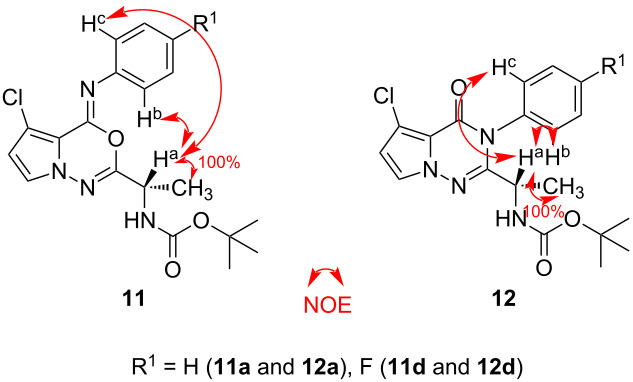				

Entry	R^1^	Product	NOEs	
			H^a^–H^b^ (%)	H^a^–H^c^ (%)

1	H	**11a**	11	6
2	F	**11d**	5	2
3	H	**12a**	39	21
4	F	**12d**	42	31

Upon examination of the ^1^H NMR spectra of oxadiazines **11** and triazinones **12**, different peak patterns of the NH protons were observed (**11** – NH: 4.8 ppm, **12** – NH: 5.1 ppm, see Supporting Information File 1).

**Figure 4:**
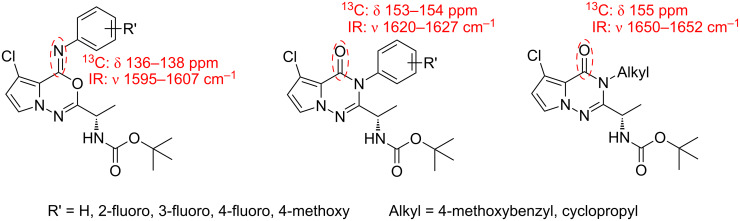
The results of ^13^C NMR and IR studies.

Through ^13^C NMR and IR analysis the presence of two regioisomers could be confirmed by the peaks of specific functional groups (Figure 4).

According to the NMR and IR data, compounds **11** and **12** are believed to have pyrrolooxadiazine and pyrrolotriazinone structures, respectively. Notably, this is the first report in which the exact structures of these regioisomers have been determined.

## Conclusion

In summary, to develop straightforward methods for the synthesis of pyrrolo[2,1-*f*][1,2,4]triazin-4(3*H*)-ones, intramolecular cyclization and rearrangement reactions were investigated. Notably, we found that triazinones **12** can be readily accessed under very mild conditions (0 °C, 5 min). The regioselectivity was influenced by the identities of halogen sources of triphenylphosphorane and the *N*-functional groups. For the rearrangement reaction, it was demonstrated that triazinone **12a** was easily obtained when counter ions of oxygen such as lithium and sodium were used. Finally, we predict that these methods could be useful for the preparation of biologically active pyrrolotriazinones and -triazines.

## Supporting Information

File 1 Experimental and analytical data.
