# Supplementary material for: Synthesis of pyrrolo[2,1-f][1,2,4]triazin-4(3H)-ones: Rearrangement of pyrrolo[1,2-d][1,3,4]oxadiazines and regioselective intramolecular cyclization of 1,2-biscarbamoyl-substituted 1H-pyrroles
Source: Beilstein J Org Chem. 2016 Aug 9;12:1780–7. doi: 10.3762/bjoc.12.168 (PMC5082444; doi:10.3762/bjoc.12.168)

Supporting Information

for

Synthesis of pyrrolo[2,1-*f*][1,2,4]triazin-4(3*H*)-ones: Rearrangement of pyrrolo[1,2-*d*][1,3,4]oxadiazines and regioselective intramolecular cyclization of 1,2-biscarbamoyl-substituted 1*H*-pyrroles

Kkonnip Son[1,2] and Seong Jun Park[1,*]

Address: [1]Research Center for Medicinal Chemistry, Korea Research Institute of Chemical Technology (KRICT), 141 Gajeong-ro, Yuseong-gu, Daejeon 305-600, Korea and [2]Department of Chemistry, Sogang University, 35 Baekbeom-ro, Mapo-gu, Seoul 121-742, Korea

Email: Seong Jun Park - sjunpark@krict.re.kr

*Corresponding author

Experimental and analytical data

Content

Experimental section S2

NMR spectra S38

NOE of **11a** and **12a** S79

With Mosher's acid for confirming enantiomeric excess (ee) value (OCH_3 peak was used for calculations) S81

Different peak patterns in the NH protons (^{1}H NMR spectra, representative examples, **11a**/**11d** and **12a**/**12d**) S84

Experimental section

General information

Analytical thin layer chromatography (TLC) was performed on Kieselgel 60 F_{254} glass plates precoated with a 0.2 mm thickness of silica gel. The TLC plates were visualized by UV (254 nm), potassium permanganate or ceric ammonium molybdate stain. Flash chromatography was carried out with Kieselgel 60 (230–400 mesh) silica gel. Melting points: Barnstead / Elecrothermal 9300, measurements were performed in open glass capillaries. IR spetra: Bruker ALPHA-P & ALPHA-T. NMR spectra: Bruker AV 300 MHz (^{1}H NMR: 300 MHz, ^{13}C NMR: 75 MHz), AV 500 MHz (^{1}H NMR: 500 NHz, ^{13}C NMR: 125 MHz), AV2 500 MHz (^{19}F NMR: 470 MHz), the spectra were recorded in $CDCl_3$ and DMSO-d_6 using TMS as internal standard and are reported in ppm. ^{1}H NMR data are reported as: (s = singlet, d = doublet, t = triplet, q = quartet, br = broad singlet, qui = quintet, oct = octet, m = multiplet; coupling constant(s) *J* are given in Hz; integration, proton assignment). High resolution mass spectra (HRMS): JEOL JMS-700. All solvents were purified using column filter solvent purification system before use unless otherwise indicated. Reagents were purchased and used without further purification.

3-Chloro-*N*-phenyl-1*H*-pyrrole-2-carboxamide (14a)

To a solution of 3-chloro-1*H*-pyrrole-2-carboxylic acid (**13**, 199.39 mg, 1.37 mmol) in dry CH_2Cl_2 (5 mL) was added oxalyl chloride (0.18 mL, 2.06 mmol) and DMF (2 drops) at 0 °C. The reaction was stirred at 70 °C for 5 h. The solvent was removed under reduced pressure, the resulting crude residue was then dried under high vaccum and used in the next reaction without further purification.

To a solution of 3-chloro-1*H*-pyrrole-2-carbonyl chloride (225.34 mg, 1.37 mmol) in 1,4-dioxane (5 mL) was aniline (0.15 mL, 1.65 mmol) and DIPEA (0.72 mL, 4.12 mmol) at 0 °C. The mixture was stirred at 60 °C for 1 h, solvent was concentrated under reduced pressure. The mixture was diluted with EtOAc and H_2O, and the layers were separated. The organic layer was dried over anhydrous Na_2SO_4, filtered and evaporated. The resulting crude residue was purified by column chromatography on silica gel (EtOAc/*n*-hexane 1:10) to give 3-chloro-*N*-phenyl-1*H*-pyrrole-2-carboxamide (**14a**, 302 mg, Quan.) as a white solid.

mp. 144 °C; ^{1}H NMR (300 MHz, $CDCl_3$) δ 10.13 (br, 1H, Ph-N*H*-CO-), 8.61 (s, 1H, pyrrolyl N*H*), 7.66 (d, *J* = 8.1 Hz, 2H, phenyl), 7.40 (t, *J* = 7.8 Hz, 2H, phenyl), 7.18 (t, *J* = 7.4 Hz, 1H, phenyl), 6.94 (t, *J* = 2.7 Hz, 1H, pyrrolyl), 6.30 (t, *J* = 2.7 Hz, 1H, pyrrolyl); ^{13}C NMR (125 MHz, $CDCl_3$) δ 158.0 (CO), 137.6 (C, phenyl), 129.1 (2 x CH, phenyl), 124.5 (CH, pyrrolyl), 121.3 (C, pyrrolyl), 121.1 (CH, phenyl), 120.2 (2 x CH, phenyl), 112.8 (C, pyrrolyl), 111.1 (CH, pyrrolyl).

1-Amino-3-chloro-*N*-phenyl-1*H*-pyrrole-2-carboxamide (15a)

An aqueous solution of sodium hydroxide (28 wt %, 18.5 mL, 130.0 mmol) was added to ammonium chloride (2.1 g, 39.0 mmol) at 0 °C. Then, an aqueous solution of ammonium hydroxide (25 wt %, 8.1 mL, 65.0 mmol), aliquat 336 (0.34 mL, 0.65 mmol), a solution of 3-chloro-*N*-phenyl-1*H*-pyrrole-2-carboxamide (1.43 g, 6.50 mmol,) in *tert*-butylmethyl ether/diethyl ether (1:1) and sodium hypochlorite (10%, 44.0 mL, 65.0 mmol) was added at 0 °C. After stirring at room temperature overnight, the reaction mixture was extracted with EtOAc. The organic layer was dried over Na_2SO_4, filtered and evaporated. The residue was purified by column

chromatography on silica gel (EtOAc/*n*-hexane 1:10) to give 1-amino-3-chloro-*N*-phenyl-1*H*-pyrrole-2-carboxamide (**15a**, 1.07 g, 70%) as a white solid.

mp. 139.9-141.0 °C; ^{1}H NMR (300 MHz, $CDCl_3$) δ 8.52 (s, 1H, pyrrolyl N*H*), 7.58 (d, *J* = 3.4 Hz, 2H, phenyl), 7.37 (t, *J* = 8.0 Hz, 2H, phenyl), 7.15 (t, *J* = 7.5 Hz, 1H, phenyl), 6.90 (d, *J* = 1.1 Hz, 1H, pyrrolyl), 6.07 (d, J = 1.3 Hz, 1H, pyrrolyl), 5.90 (s, 2H, NH_2-pyrrolyl); ^{13}C NMR (125 MHz, $CDCl_3$) δ 158.9 (CO), 137.4 (C, phenyl), 129.1 (2 x CH, phenyl), 126.4 (CH, pyrrolyl), 124.7 (CH, phenyl), 120.5 (2 x CH, phenyl), 118.8 (C, pyrrolyl), 112.6 (C, pyrrolyl), 106.4 (CH, pyrrolyl).

***tert*-Butyl (*S*)-(1-((3-chloro-2-(phenylcarbamoyl)-1*H*-pyrrol-1-yl)amino)-1-oxopropan-2-yl)carbamate (10a)**

To a solution of 1-amino-3-chloro-*N*-phenyl-1*H*-pyrrole-2-carboxamide (**15a**, 2.3 g, 9.76 mmol) in THF (30 mL) was added *N*-(*tert*-butoxycarbonyl)-L-alanine (2.59 g, 13.66 mmol) and 1-(3-dimethylaminopropyl)-3-ethylcarbodiimide hydrochloride (2.62 g, 13.66 mmol) at room temperature. After stirring for 17 h, the reaction mixture was extracted with EtOAc. The organic layer was dried over anhydrous $MgSO_4$, filtered and evaporated. The resulting crude residue was purified by column chromatography on silica gel (EtOAc/*n*-hexane 1:10) to give *tert*-butyl (*S*)-(1-((3-chloro-2-(phenylcarbamoyl)-1*H*-pyrrol-1-yl)amino)-1-oxopropan-2-yl)carbamate (**10a**, 3.48 g, 88%) as a white solid.

mp. 150.9-152.2 °C; IR (KBr): ν 1672, 1637, 1550, 1524, 1442, 1390, 1367, 1253, 1159, 753 cm^{-1}; LC/MS: R_t = 3.01 mins, m/z (ES+) = 407 (M+H for $C_{19}H_{23}ClN_4O_4$); ^{1}H NMR (300 MHz, $CDCl_3$) δ 10.38 (br, 1H, Ph-N*H*-CO-), 8.38 (s, 1H, -N*H*-pyrrolyl), 7.54 (d, *J* = 7.8 Hz, 2H, phenyl), 7.32 (t, *J* = 7.8 Hz, 2H, phenyl), 7.12 (t, *J* = 7.4 Hz,

1H, phenyl), 6.93 (s, 1H, pyrrolyl), 6.18 (d, *J* = 3.3 Hz, 1H, pyrrolyl), 5.24 (br, 1H, Boc-N*H*-), 4.43 (br, 1H, CH_3-C*H*(NH-Boc)-CO-), 1.45 (s, 9H, *t*-butyl), 1.42 (d, *J* = 7.2 Hz, 3H, CH_3-CH(NH-Boc)-CO-); ^{13}C-NMR (125 MHz, $CDCl_3$) δ 172.9 (CO), 157.6 (CO), 155.6 (CO), 137.3 (C, phenyl), 129.0 (2 x CH, phenyl), 126.8 (C, pyrrolyl), 124.7 (CH, phenyl), 120.4 (2 x CH, phenyl), 119.2 (C, pyrrolyl), 113.8 (C, pyrrolyl), 107.7 (CH, pyrrolyl), 80.6 (C), 49.0 (CH), 28.3 (3 x CH_3), 17.6 (CH_3).

***tert*-Butyl (*S*,*Z*)-(1-(5-chloro-4-(phenylimino)-4*H*-pyrrolo[1,2-*d*][1,3,4]oxadiazin-2-yl)ethyl)carbamate (11a) and *tert*-butyl (*S*)-(1-(5-chloro-4-oxo-3-phenyl-3,4-dihydropyrrolo[2,1-*f*][1,2,4]triazin-2-yl)ethyl)carbamate (12a)**

To a solution of triphenylphosphine (129 mg, 0.49 mmol) in CH_2Cl_2 (0.5 mL) was added bromine (0.01 mL, 0.54 mmol) at 0 °C. The mixture was stirred at room temperature for 15 min. Then, a solution of aminopyrrolocarbamate (**10a**, 100 mg, 0.25 mmol) in CH_2Cl_2 (0.5 mL) and triethylamine (0.17 mL, 1.23 mmol) were added at 0 °C. After complete addition, the reaction mixture was stirred at 0 °C for 5 min. Then, the reaction mixture was extracted with CH_2Cl_2. The organic layer was dried over anhydrous $MgSO_4$, filtered and evaporated. The resulting crude residue was purified by column chromatography on silica gel (EtOAc/*n*-hexane 1:10) to give iminooxadiazinone (**11a**, 65 mg, 68%) and triazinone (**12a**, 21 mg, 22%) as a white solid.

11a: mp. 156.2-157.0 °C; IR (KBr): ν 1680, 1595, 1525, 1249, 1162, 997 cm^{-1}; ^{1}H NMR (300 MHz, $CDCl_3$) δ 7.33 (t, *J* = 7.8 Hz, 2H, phenyl), 7.14 (d, *J* = 8.4 Hz, 3H, phenyl) 7.06 (d, *J* = 3.0 Hz, 1H, pyrrolyl), 6.37 (d, *J* = 3.0 Hz, 1H, pyrrolyl), 4.84 (s, 1H, Boc-N*H*-), 4.50 (t, *J* = 6.3 Hz, 1H, CH_3-C*H*(NH-Boc)-C-), 1.43 (s, 9H, *t*-butyl),

1.37 (d, J = 6.9 Hz, 3H, CH_3-CH(NH-Boc)-C-); ^{13}C-NMR (125 MHz, $CDCl_3$) δ 154.8 (CO), 154.7 (C), 144.0 (C, phenyl), 136.3 (C=N), 128.7 (2 x CH, phenyl), 124.5 (CH, pyrrolyl), 122.9 (2 x CH, phenyl), 120.3 (CH, phenyl), 114.5 (C, pyrrolyl), 110.9 (CH, pyrrolyl), 110.3 (C, pyrrolyl), 80.4 (C), 47.5 (CH), 28.3 (3 x CH_3), 19.0 (CH_3); HRMS (EI) calcd for $C_{19}H_{21}ClN_4O_3$ 388.1302, found 388.1306.

12a: mp. 183.8-185 °C; IR (KBr): ν 1710, 1685, 1624, 1527, 1402, 1336, 1246, 1171, 1069, 756 cm^{-1}; ^{1}H NMR (300 MHz, $CDCl_3$) δ 7.51-7.60 (m, 3H, phenyl), 7.42 (d, J = 6.9 Hz, 1H, phenyl), 7.28-7.30 (m, 2H, phenyl and pyrrolyl), 6.50 (d, J = 2.7 Hz, 1H, pyrrolyl), 5.17 (d, J = 8.4 Hz, 1H, Boc-N*H*-), 4.49 (qui, J = 6.9 Hz, 1H, CH_3-C*H*(NH-Boc)-C-), 1.43 (s, 9H, *t*-butyl), 1.26 (d, J = 6.9 Hz, 3H, CH_3-CH(NH-Boc)-C-); ^{13}C NMR (125 MHz, $CDCl_3$) δ 154.6 (CO), 153.8 (CO), 151.7 (C), 134.2 (C, phenyl), 130.2 (CH, pyrrolyl), 129.7 (2 x CH, phenyl), 129.5 (CH, phenyl), 129.3 (CH, phenyl), 129.2 (CH, phenyl), 119.9 (CH, phenyl), 114.0 (C, pyrrolyl), 113.9 (C, pyrrolyl), 111.4 (CH, pyrrolyl), 80.0 (C), 46.7 (CH), 28.4 (3 x CH_3), 20.4 (CH_3); HRMS (EI) calcd for $C_{19}H_{21}ClN_4O_3$ 388.1302, found 388.1300.

3-Chloro-*N*-(2-fluorophenyl)-1*H*-pyrrole-2-carboxamide (14b)

To a solution of 3-chloro-1*H*-pyrrole-2-carboxylic acid (**13**, 615.63 mg, 4.23 mmol) in dry CH_2Cl_2 (20 mL) was added oxalyl chloride (0.54 mL, 6.35 mmol) and DMF (2 drops) at 0 °C. The reaction was stirred at 70 °C for 5 h. The solvent was removed under reduced pressure, the resulting crude residue was then dried under high vaccum and used to the next reaction without further purification.

To a solution of 3-chloro-1*H*-pyrrole-2-carbonyl chloride (700 mg, 4.23 mmol) in 1,4-dioxane (7 mL) was added 2-fluoroaniline (0.52 mL, 5.34 mmol) and DIPEA (2.23 mL,

12.81 mmol) at 0 °C. The mixture was stirred at 60 °C for 5 h, a solvent was concentrated under reduced pressure. The mixture was diluted with EtOAc and H_2O, and the layers were separated. The organic layer was dried over anhydrous $MgSO_4$, filtered and evaporated. The resulting crude residue was purified by column chromatography on silica gel (EtOAc/*n*-hexane 1:5) to give 3-chloro-*N*-(2-fluorophenyl)-1*H*-pyrrole-2-carboxamide (**14b**, 897.7 mg, 88%) as a white solid.

mp. 148.0-150.0 °C; ^{1}H NMR (300 MHz, $CDCl_3$) δ 10.07 (br, 1H, 2-fluorophenyl-N*H*-CO-), 8.93 (s, 1H, pyrrolyl N*H*), 8.44 (t, *J* = 8.1 Hz, 1H, 2-fluorophpenyl), 7.07-7.20 (m, 3H, 2-fluorophenyl), 6.93 (t, *J* = 2.9 Hz, 1H, pyrrolyl), 6.29 (t, *J* = 2.4 Hz, 1H, pyrrolyl); ^{13}C NMR (125 MHz, $CDCl_3$) δ 157.9 (CO), 152.6 (C, d, *J* = 244.4 Hz, phenyl), 126.4 (C, d, *J* = 10.1 Hz, phenyl), 124.6 (C, d, *J* = 3.8 Hz, phenyl), 124.3 (CH, d, *J* = 7.6 Hz, phenyl), 121.6 (CH, pyrrolyl), 121.4 (CH, phenyl), 121.3 (C, pyrrolyl), 114.9 (CH, d, *J* = 18.9 Hz, phenyl), 113.5 (C, pyrrolyl), 111.3 (CH, pyrrolyl).

1-Amino-3-chloro-*N*-(2-fluorophenyl)-1*H*-pyrrole-2-carboxamide (15b)

An aqueous solution of sodium hydroxide (28 wt %, 10.5 mL, 73.50 mmol) was added to ammonium chloride (1.18 g, 22.05 mmol) at 0 °C. Then, an aqueous solution of ammonium hydroxide (25 wt %, 10.3 mL, 73.50 mmol), aliquat 336 (0.17 mL, 0.37 mmol), a solution of 3-chloro-*N*-(2-fluorophenyl)-1*H*-pyrrole-2-carboxamide (876.8 mg, 3.67 mmol) in *tert*-butylmethyl ether/diethyl ether (1:1, 10 mL) and sodium hypochlorite (10%, 24.65 mL, 36.75 mmol) was added at 0 °C. After stirring at room temperature overnight, the reaction mixture was extracted with EtOAc. The organic layer was dried over $MgSO_4$, filtered and evaporated. The residue was purified by column chromatography on silica gel (EtOAc/*n*-hexane 1:5) to give 1-

amino-3-chloro-*N*-(2-fluorophenyl)-1*H*-pyrrole-2-carboxamide (**15b**, 140.6 mg, 15%) as a yellow solid.

mp. 179.0-180.3 °C; ^{1}H NMR (300 MHz, $CDCl_3$) δ 8.90 (br, 1H, 2-fluorophenyl-N*H*-CO-), 8.36 (t, *J* = 8.1 Hz, 1H, 2-fluorophenyl), 7.05-7.19 (m, 3H, 2-fluorophenyl), 6.91 (d, *J* = 1.1 Hz, 1H, pyrrolyl), 6.09 (d, *J* = 1.3 Hz, 1H, pyrrolyl), 5.89 (s, 2H, NH_2-pyrrolyl); ^{13}C NMR (125 MHz, $CDCl_3$) δ 158.9 (CO), 152.8 (C, d, *J* = 244.4 Hz, phenyl), 126.8 (CH, pyrrolyl), 126.2 (C, d, *J* = 10.1 Hz, phenyl), 124.6 (CH, d, *J* = 3.8 Hz, phenyl), 124.5 (CH, d, *J* = 7.6 Hz, phenyl), 121.9 (CH, phenyl), 118.8 (C, pyrrolyl), 115.0 (CH, d, *J* = 18.9 Hz, phenyl), 113.4 (C, pyrrolyl), 106.6 (CH, pyrrolyl).

tert-Butyl (_S_)-(1-((3-chloro-2-((2-fluorophenyl)carbamoyl)-1_H_-pyrrol-1-yl)amino)-1-oxopropan-2-yl)carbamate (10b)

To a solution of 1-amino-3-chloro-*N*-(2-fluorophenyl)-1*H*-pyrrole-2-carboxamide (**15b**, 140.6 mg, 0.55 mmol) in THF (5 mL) was added *N*-(*tert*-butoxycarbonyl)-L-alanine (146.8 mg, 0.78 mmol**)** and 1-(3-dimethylaminopropyl)-3-ethylcarbodiimide hydrochloride (148.74 mg, 0.78 mmol) at room temperature. After stirring overnight, the reaction mixture was extracted with EtOAc. The organic layer was dried over anhydrous $MgSO_4$, filtered and evaporated. The resulting crude residue was purified by column chromatography on silica gel (EtOAc/*n*-hexane 1:2) to give *tert*-butyl (*S*)-(1-((3-chloro-2-((2-fluorophenyl)carbamoyl)-1*H*-pyrrol-1-yl)amino)-1-oxopropan-2-yl)carbamate (**10b**, 164.4 mg, 70%) as a white solid.

mp. 156.8-158.0 °C; IR (KBr): ν 1712, 1687, 1618, 1599, 1541, 1520, 1487, 1453, 1409, 1255, 1164, 755 cm^{-1}; LC/MS: R_t = 3.10 mins, m/z (ES+) = 425 (M+H for $C_{19}H_{22}ClFN_4O_4$); ^{1}H NMR (300 MHz, $CDCl_3$) δ 10.23 (br, 1H, 2-fluorophenyl-N*H*-CO-),

8.72 (s, 1H, -N*H*-pyrrolyl), 8.32 (t, *J* = 6.6 Hz, 1H, 2-fluorophenyl), 7.07-7.11 (m, 3H, 2-fluorophenyl), 7.00 (d, *J* = 1.1 Hz, 1H, pyrrolyl), 6.22 (d, *J* = 1.3 Hz, 1H, pyrrolyl), 5.12 (s, 1H, Boc-N*H*-), 4.43 (s, 1H, CH_3-C*H*(NH-Boc)-CO-), 1.47 (s, 12H, *t*-butyl and CH_3-CH(NH-Boc)-CO-); ^{13}C NMR (125 MHz, $CDCl_3$) δ 172.8 (CO), 157.5 (CO), 155.7 (CO), 152.7 (C, d, *J* = 244.4 Hz, 2-fluorophenyl), 127.2 (CH, 2-fluorophenyl), 126.1 (C, d, *J* = 10.1 Hz, 2-fluorophenyl), 124.6 (CH, d, *J* = 7.6 Hz, 2-fluorophenyl), 124.5 (CH, d, *J* = 3.8 Hz, 2-fluorophenyl), 121.9 (CH, pyrrolyl), 119.0 (C, pyrrolyl), 115.0 (CH, d, *J* = 18.9 Hz, 2-fluorophenyl), 114.6 (C, pyrrolyl), 107.9 (CH, pyrrolyl), 80.7 (C), 49.0 (CH), 28.4 (3 x CH_3), 17.5 (CH_3).

***tert*-Butyl (*S*,*Z*)-(1-(5-chloro-4-((2-fluorophenyl)imino)-4*H*-pyrrolo[1,2-*d*][1,3,4]oxadiazin-2-yl)ethyl)carbamate (11b) and *tert*-butyl (*S*)-(1-(5-chloro-3-(2-fluorophenyl)-4-oxo-3,4-dihydropyrrolo[2,1-*f*][1,2,4]triazin-2-yl)ethyl)carbamate (12b)**

To a solution of triphenylphosphine (142.74 mg, 0.54 mmol) in CH_2Cl_2 (0.5 mL) was added bromine (0.03 mL, 0.54 mmol) at 0 °C. The mixture was stirred at room temperature for 15 min. Then, a solution of aminopyrrolocarbamate (**10b**, 115.6 mg, 0.27 mmol) in CH_2Cl_2 (0.5 mL) and triethylamine (0.19 mL, 1.36 mmol) were added at 0 °C. After complete addition, the reaction mixture was stirred at 0 °C for 5 min. Then, the reaction mixture was extracted with CH_2Cl_2. The organic layer was dried over anhydrous $MgSO_4$, filtered and evaporated. The resulting crude residue was purified by column chromatography on silica gel (EtOAc/*n*-hexane 1:10) to give iminooxadiazinone (**11b**, 18 mg, 16%) and triazinone (**12b**, 32 mg, 29%) as a white solid.

11b: mp. 112.0-114.0 °C; IR (ATR): ν 1681, 1607, 1521, 1492, 1326, 1250, 1162, 1069, 1026, 969, 854, 741 cm^{-1}; ^{1}H NMR (300 MHz, $CDCl_3$) δ 7.09-7.20 (m, 5H, 2-fluorophenyl), 6.40 (d, *J* = 1.26 Hz, pyrrolyl), 4.83 (br, 1H, Boc-N*H*-), 4.49 (t, 1H, CH_3-C*H*(NH-Boc)-C-), 1.42 (s, 9H, *t*-butyl), 1.37 (d, *J* = 3.0 Hz, 3H, CH_3-CH(NH-Boc)-C-); ^{13}C NMR (125 MHz, $CDCl_3$) δ 154.8 (CO), 153.8 (C, d, *J* = 246.5 Hz, 2-fluorophenyl), 138.4 (C=N), 132.2 (C, d, *J* = 13.1 Hz, 2-fluorophenyl), 125.4 (CH, d, *J* = 7.4, 2-fluorophenyl), 124.7 (CH, d, *J* = 1.5 Hz, 2-fluorophenyl), , 124.2 (CH, d, *J* = 3.5 Hz, 2-fluorophenyl), 120.6 (CH, pyrrolyl), 115.7 (CH, d, *J* = 20.4 Hz, 2-fluorophenyl), 115.1 (C, pyrrolyl), 111.1 (CH, pyrrolyl), 110.1 (C, pyrrolyl), 80.3 (C), 47.4 (CH), 29.7, 28.3 (3 x CH_3), 18.8 (CH_3); ^{19}F-NMR (470 MHz) δ -123.53 (d, *J* = 9.4 Hz) ppm; HRMS (EI) calcd for $C_{19}H_{20}ClFN_4O_3$ 406.1208, found 406.1210.

12b: mp. 83.0-84.0 °C; IR (ATR): ν 1689, 1627, 1499, 1403, 1328, 1251, 1158, 1067, 1013, 761 cm^{-1}; ^{1}H NMR (300 MHz, $CDCl_3$) δ 7.40-7.52 (m, 2H, 2-fluorophenyl), 7.25-7.36 (m, 2H, 2-fluorophenyl), 6.50 (d, *J* = 1.3 Hz, 1H, pyrrolyl), 5.08 (d, *J* = 3.4 Hz, 1H, Boc-N*H*-), 4.49 (qui, *J* = 6.9 Hz, 1H, CH_3-C*H*(NH-Boc)-C-), 1.28-1.41 (m, 12H, *t*-butyl and C*H*$_3$-CH(NH-Boc)-C-); ^{13}C NMR (125 MHz, $CDCl_3$) δ 158.5 (d, *J* = 251.9 Hz), 154.7 (CO), 152.8 (CO), 151.4 (C), 131.9 (CH, d, *J* = 7.8 Hz, 2-fluoropheyl), 131.2 (CH, 2-fluorophenyl), 125.5 (CH, d, *J* = 2.5 Hz, 2-fluorophenyl), 121.9 (C, d, *J* = 13.6 Hz, 2-fluorophenyl), 120.2 (CH, pyrrolyl), 116.8 (CH, d, *J* = 19.8 Hz, 2-fluorophenyl), 114.5 (C, pyrrolyl), 113.9 (C, pyrrolyl), 111.5 (CH, pyrrolyl), 80.1 (C), 53.4, 46.8 (CH), 29.7, 28.3 (3 x CH_3), 19.8 (CH_3); ^{19}F-NMR (470 MHz) δ -120.52 ppm; HRMS (EI) calcd for $C_{19}H_{20}ClFN_4O_3$ 406.1208, found 406.1200.

3-Chloro-*N*-(3-fluorophenyl)-1*H*-pyrrole-2-carboxamide (14c)

To a solution of 3-chloro-1*H*-pyrrole-2-carboxylic acid (**13**, 2 g, 13.75 mmol) in dry CH_2Cl_2 (50 mL) was added oxalyl chloride (1.77 mL, 20.63 mmol) and DMF (2 drops) at 0 °C. The reaction was stirred at 70 °C for 4 h. The solvent was removed under reduced pressure, the resulting crude residue was then dried under high vaccum and used to the next reaction without further purification.

To a solution of 3-chloro-1*H*-pyrrole-2-carbonyl chloride (2.56 g, 13.75 mmol) in 1,4-dioxane (20 mL) was 3-fluoroaniline (1.65 mL, 17.18 mmol) and DIPEA (7.28 mL, 41.78 mmol) at 0 °C. The mixture was stirred at 60 °C for 3 h, a solvent was concentrated under reduced pressure. The mixture was diluted with EtOAc and H_2O, and the layers were separated. The organic layer was dried over anhydrous Na_2SO_4, filtered and evaporated. Then, the resulting crude residue was filtered and the filtrate was evaporated to give 3-chloro-*N*-(3-fluorophenyl)-1*H*-pyrrole-2-carboxamide (**14c**, 2.59 g, 67%. 2 steps) as a white solid.

mp. 158.0-158.4 °C; ^{1}H NMR (300 MHz, $CDCl_3$) δ 10.04 (br, 1H, 3-fluorophenyl-N*H*-CO-), 8.66 (s, 1H, pyrrolyl N*H*), 7.66 (d, *J* = 4.5 Hz, 1H, 3-fluorophenyl), 7.23-7.37 (m, 2H, 3-fluorophenyl), 6.96 (s, 1H, pyrrolyl), 6.87 (t, *J* = 8.0 Hz, 3-fluorophenyl), 6.31 (s, 1H, pyrrolyl); ^{13}C NMR (125 MHz, $CDCl_3$) δ 163.1 (C, d, *J* = 245.7 Hz, 3-fluorophenyl), 157.9 (CO), 139.1 (C, d, *J* = 10.1 Hz, 3-fluorophenyl), 130.2 (CH, d, *J* = 10.1 Hz, 3-fluorophenyl), 121.5 (CH, pyrrolyl), 121.0 (C, pyrrolyl), 115.3 (CH, d, *J* = 2.5 Hz, 3-fluorophenyl), 113.1 (C, pyrrolyl), 111.3 (CH, pyrrolyl), 111.2 (CH, d, *J* = 21.4 Hz, 3-fluorophpenyl), 107.7 (CH, d, *J* = 26.5 Hz, 3-fluorophenyl).

1-Amino-3-chloro-*N*-(3-fluorophenyl)-1*H*-pyrrole-2-carboxamide (15c)

An aqueous solution of sodium hydroxide (28 wt %, 46 mL, 324 mmol) was added to ammonium chloride (5.20 g, 97 mmol) at 0 °C. Then, an aqueous solution of ammonium hydroxide (28wt%, 40 mL, 324 mmol), aliquat 336 (0.791 mL, 2 mmol), a solution of 3-chloro-*N*-(3-fluorophenyl)-1*H*-pyrrole-2-carboxamide (**14c**, 2.59 g, 11 mmol) in *tert*-butylmethyl ether/diethyl ether (1:1, 80 mL) and sodium hypochlorite (10%, 109 mL, 162 mmol) was added at 0 °C. After stirring at room temperature for 1 h, the reaction mixture was extracted with EtOAc. The organic layer was dried over $MgSO_4$, filtered and evaporated. The residue was purified by column chromatography on silica gel (EtOAc/*n*-hexane 1:2) to give 1-amino-3-chloro-*N*-(3-fluorophenyl)-1*H*-pyrrole-2-carboxamide (**15c**, 1.72 g, 63%) as a yellow solid.

mp. 153.7-154.0 °C ℃; ^{1}H NMR (300 MHz, $CDCl_3$) δ 8.60 (br, 1H, 3-fluorophenyl-N*H*-CO-), 7.55-7.60 (m, 1H, 3-fluorophenyl), 7.27-7.35 (m, 1H, 3-fluorophenyl), 7.19-7.23 (m, 1H, 3-fluorophenyl), 6.92 (d, *J* = 1.3 Hz, 1H, prrrolyl), 6.82-6.88 (m, 1H, 3-fluorophenyl), 6.09 (d, *J* = 1.3 Hz, 1H, pyrrolyl), 5.88 (s, 2H, NH_2-pyrrolyl).

***tert*-Butyl (*S*)-(1-((3-chloro-2-((3-fluorophenyl)carbamoyl)-1*H*-pyrrol-1-yl)amino)-1-oxopropan-2-yl)carbamate (10c)**

To a solution of 1-amino-3-chloro-*N*-(3-fluorophenyl)-1*H*-pyrrole-2-carboxamide (**15c**, 3.11 g, 12.26 mmol) in THF (40 mL) was added *N*-(*tert*-butoxycarbonyl)-L-alanine (3.25 g, 17.16 mmol) and 1-(3-dimethylaminopropyl)-3-ethylcarbodiimide hydrochloride (3.29 g, 17.16 mmol) at room temperature. After stirring for 15 h, the reaction mixture was extracted with EtOAc. The organic layer was dried over anhydrous $MgSO_4$, filtered and evaporated. The resulting crude residue was purified

by column chromatography on silica gel (EtOAc/*n*-Hexane, 1:10) to give *tert*-butyl (*S*)-(1-((3-chloro-2-((3-fluorophenyl)carbamoyl)-1*H*-pyrrol-1-yl)amino)-1-oxopropan-2-yl)carbamate (**10c**, 5.21 g, quantitative) as a white solid.

mp. 139.0-140.0 °C; IR (KBr): ν 1672, 1641, 1606, 1549, 1526, 1443, 1251, 1160, 777 cm^{-1}; LC/MS: R_t = 3.08 mins, m/z (ES+) = 425 (M+H for $C_{19}H_{22}ClFN_4O_4$); ^1H NMR (300 MHz, $CDCl_3$) δ 10.31 (br, 1H, 3-fluorophenyl-N*H*-CO-), 8.45 (s, 1H, -N*H*-pyrrolyl), 7.59 (d, *J* = 4.7 Hz, 1H, 3-fluorophenyl), 7.22-7.30 (m, 1H, 3-fluorophenyl), 7.10-7.13 (m, 1H, 3-fluorophenyl), 7.00 (d, *J* = 1.0 Hz, 1H, pyrrolyl), 6.79-6.85 (m, 1H, 3-fluorophenyl), 6.19 (d, *J* = 1.3 Hz, 1H, pyrrolyl), 5.15 (br, 1H, Boc-N*H*-), 4.42 (br, 1H, CH_3-C*H*(NH-Boc)-CO-), 1.46 (s, 9H, *t*-butyl), 1.43 (d, *J* = 3.0 Hz, 3H, CH_3-CH(NH-Boc)-CO-); ^{13}C NMR (125 MHz, $CDCl_3$) δ 173.0 (CO), 163.0 (C, d, *J* = 244.4 Hz, 3-fluorophenyl), 157.4 (CO), 155.7 (CO), 138.9 (C, d, *J* = 11.3 Hz, 3-fluorophenyl), 130.0 (CH, d, *J* = 10.1 Hz, 3-fluorophenyl), 127.2 (CH, pyrrolyl), 119.3 (C, pyrrolyl), 115.4 (CH, d, *J* = 2.5 Hz, 3-fluorophenyl), 114.1 (C, pyrrolyl), 111.2 (CH, d, *J* = 21.4 Hz, 3-fluorophenyl), 107.9 (CH, pyrrolyl), 107.7 (CH, d, *J* = 26.5 Hz, 3-fluorophenyl), 80.7 (C), 48.8 (CH), 28.3 (3 x CH_3), 17.5 (CH_3).

***tert*-Butyl (*S*,*Z*)-(1-(5-chloro-4-((3-fluorophenyl)imino)-4*H*-pyrrolo[1,2-*d*][1,3,4]oxadiazin-2-yl)ethyl)carbamate (11c) and *tert*-butyl (*S*)-(1-(5-chloro-3-(3-fluorophenyl)-4-oxo-3,4-dihydropyrrolo[2,1-*f*][1,2,4]triazin-2-yl)ethyl)carbamate (12c)**

To a solution of triphenylphosphine (129 mg, 0.49 mmol) in CH_2Cl_2 (0.5 mL) was added bromine (0.01 mL, 0.54 mmol) at 0 °C. The mixture was stirred at room temperature for 15 min. Then, a solution of aminopyrrolocarbamate (**10c**, 100 mg,

0.25 mmol) in CH_2Cl_2 (0.5 mL) and triethylamine (0.17 mL, 1.23 mmol) were added at 0 °C. After complete addition, the reaction mixture was stirred at 0 °C for 5 min. Then, the reaction mixture was extracted with CH_2Cl_2. The organic layer was dried over anhydrous $MgSO_4$, filtered and evaporated. The resulting crude residue was purified by column chromatography on silica gel (EtOAc/*n*-hexane 1:10) to give iminooxadiazinone (**11c**, 25 mg, 25%) and triazinone (**12c**, 69 mg, 68%) as a white solid.

11c: mp. 155.5-156.3 °C; IR (KBr): ν 1680, 1602, 1585, 1521, 1462, 1324, 1250, 1161, 1133, 1067, 1027, 969, 944, 862, 782, 753 cm^{-1}; ^1H NMR (300 MHz, $CDCl_3$) δ 7.18-7.31 (m, 1H, 3-fluorophenyl), 7.08 (d, *J* = 1.3 Hz, 1H, pyrrolyl), 6.79-6.93 (m, 3H, 3-fluorophenyl), 6.38 (d, *J* = 1.3 Hz, 1H, pyrrolyl), 4.87 (br, 1H, Boc-N*H*-), 4.50 (br, 1H, CH_3-C*H*(NH-Boc)-C-), 1.43 (s, 9H, *t*-butyl), 1.39 (d, *J* = 3.0 Hz, 3H, CH_3-CH(NH-Boc)-C-); ^{13}C NMR (125 MHz, $CDCl_3$) δ 163.0 (C, d, *J* = 245.7 Hz, 3-fluorophenyl), 154.7 (CO), 145.7 (C, d, *J* = 10.1 Hz, 3-fluorophenyl), 137.1 (C=N), 129.7 (CH, d, *J* = 8.8 Hz, 3-fluorophenyl)), 120.6 (CH, 3-fluorophenyl), 118.7 (CH, pyrrolyl), 114.9 (C, pyrrolyl), 111.2 (CH, d, *J* = 21.4 Hz, 3-fluorophenyl), 111.1 (CH, pyrrolyl), 110.1 (CH, d, *J* = 23.9 Hz, 3-fluorophenyl), 110.0 (C, pyrrolyl), 80.4 (C), 47.5 (CH), 28.3 (3 x CH_3), 18.9 (CH_3); ^{19}F-NMR (470 MHz) δ -113.93 ppm; HRMS (EI) calcd for $C_{19}H_{20}ClFN_4O_3$ 406.1208, found 406.1202.

12c: mp. 98.0 °C; IR (ATR): ν 1684, 1625, 1599, 1488, 1404, 1334, 1241, 1160, 1044, 758 cm^{-1}; ^1H NMR (300 MHz, $CDCl_3$) δ 7.47-7.58 (m, 1H, 3-fluorophenyl), 7.29 (d, *J* = 1.3 Hz, 1H, pyrrolyl), 7.15-7.23 (m, 2H, 3-fluorophenyl), 7.02-7.09 (m, 1H, 3-fluorophenyl), 6.51 (d, *J* = 1.3 Hz, 1H, pyrrolyl), 5.05 (m, 1H, Boc-N*H*-), 4.48 (br, 1H,

CH_3-C*H*(NH-Boc)-C-), 1.42 (s, 9H, *t*-butyl), 1.29 (t, *J* = 6.3 Hz, 3H, CH_3-CH(NH-Boc)-C-); ^{13}C NMR (125 MHz, $CDCl_3$) δ 164.2 (C, d, *J* = 250.7 Hz, 3-fluorophenyl), 162.8 (C, d, *J* = 249.5 Hz, 3-fluorophenyl), 154.7, 154.5, 153.54, 153.48, 151.38, 151.09, 135.6, 135.5, 131.3 (CH, d, *J* = 7.6 Hz, 3-fluorophenyl), 130.7 (CH, d, *J* = 7.6 Hz, 4-fluorophenyl), 125.2 (CH, d, *J* = 13.9 Hz, 3-fluorophenyl), 120.2 (CH, pyrrolyl), 117.2 (CH, d, *J* = 8.8 Hz, 3-fluorophenyl), 117.0 (CH, d, *J* = 8.8 Hz, 3-fluorophenyl), 114.4 (C, pyrrolyl), 113.9 (C, pyrrolyl), 111.6 (CH, pyrrolyl), 80.24, 80.18, 53.4, 46.7, 29.7, 28.34, 28.28, 20.5, 20.2; ^{19}F-NMR (470 MHz) δ -110.40 (d, *J* = 6.1 Hz) and -111.52 (d, *J* = 6.6 Hz) ppm; HRMS (EI) calcd for $C_{19}H_{20}ClFN_4O_3$ 406.1208, found 406.1205.

3-Chloro-*N*-(4-fluorophenyl)-1*H*-pyrrole-2-carboxamide (14d)

To a solution of 3-chloro-1*H*-pyrrole-2-carboxylic acid (**13**, 615.63 mg, 4.23 mmol) in dry CH_2Cl_2 (20 mL) was added oxalyl chloride (0.54 mL, 6.35 mmol) and DMF (2 drops) at 0 °C. The reaction was stirred at 70 °C for 5 h. The solvent was removed under reduced pressure, the resulting crude residue was then dried under high vaccum and used to the next reaction without further purification.

To a solution of 3-chloro-1*H*-pyrrole-2-carbonyl chloride (700 mg, 4.23 mmol) in 1,4-dioxane (7 mL) was added 4-fluoroaniline (0.51 mL, 5.34 mmol) and DIPEA (2.23 mL, 12.81 mmol) at 0 °C. The mixture was stirred at 60 °C for 3 h, a solvent was concentrated under reduced pressure. The mixture was diluted with EtOAc and H_2O, and the layers were separated. The organic layer was dried over anhydrous $MgSO_4$, filtered and evaporated. The resulting crude residue was purified by column chromatography on silica gel (EtOAc/*n*-hexane 1:5) to give 3-chloro-*N*-(4-fluorophenyl)-1*H*-pyrrole-2-carboxamide (**14d**, 904.1 mg, 89%) as a white solid.

mp. 164.0-165.8 °C; ^{1}H NMR (300 MHz, $CDCl_3$) δ 10.09 (br, 1H, 4-fluorophenyl-N*H*-CO-), 8.55 (s, 1H, pyrrolyl N*H*), 7.59 (q, *J* = 4.8 Hz and *J* = 8.7 Hz, 2H, 4-fluorophenyl), 7.07 (t, *J* = 8.6 Hz, 2H, 4-fluorophenyl), 6.91 (t, *J* = 2.9 Hz, 1H, pyrrolyl), 6.28 (t, *J* = 2.6 Hz, 1H, pyrrolyl); ^{13}C NMR (125 MHz, $CDCl_3$) δ 159.6 (C, d, *J* = 244.4 Hz, 4-fluorophenyl), 158.0 (CO), 133.5 (C, d, *J* = 2.5 Hz, 4-fluorophenyl), 122.1 (2 x CH, d, *J* = 8.8 Hz, 4-fluorophenyl), 121.2 (CH, pyrrolyl), 121.1 (C, pyrrolyl), 115.8 (2 x CH, d, *J* = 22.7 Hz, 4-fluorophenyl), 112.9 (C, pyrrolyl), 111.2 (CH, pyrrolyl).

1-Amino-3-chloro-*N*-(4-fluorophenyl)-1*H*-pyrrole-2-carboxamide (15d)

An aqueous solution of sodium hydroxide (28 wt %, 10.49 mL, 74.83 mmol) was added to ammonium chloride (1.2 g, 22.45 mmol) at 0 °C. Then, an aqueous solution of ammonium hydroxide (25 wt %, 10.49 mL, 74.83 mmol), aliquat 336 (0.17 mL, 0.37 mmol), a solution of 3-chloro-*N*-(4-fluorophenyl)-1*H*-pyrrole-2-carboxamide (**14d**, 892.7 mg, 3.74 mmol) in *tert*-butylmethyl ether/diethyl ether (1:1, 10 mL) and sodium hypochlorite (10%, 25.1 mL, 37.03 mmol) was added at 0 °C. After stirring at room temperature overnight, the reaction mixture was extracted with EtOAc. The organic layer was dried over $MgSO_4$, filtered and evaporated. The residue was purified by column chromatography on silica gel (EtOAc/*n*-hexane 1:5) to give 1-amino-3-chloro-*N*-(4-fluorophenyl)-1*H*-pyrrole-2-carboxamide (**15d**, 624.2 mg, 66%) as yellow solid.

mp. 144.3-144.8 °C; ^{1}H NMR (300 MHz, $CDCl_3$) δ 8.50 (br, 1H, 4-fluorophenyl-N*H*-CO-), 7.50-7.57 (m, 2H, 4-fluorophenyl), 7.02-7.10 (m, 2H, 4-fluorophenyl), 6.91 (d, *J* = 3.0 Hz, 1H, pyrrolyl), 6.08 (d, *J* = 3.0 Hz, 1H, pyrrolyl), 5.89 (s, 2H, NH_2-pyrrolyl); ^{13}C NMR (125 MHz, $CDCl_3$) δ 159.6 (C, d, *J* = 244.4 Hz, 4-fluorophenyl), 158.9 (CO), 133.3 (C, d, *J* = 2.5 Hz, 4-fluorophenyl), 126.5 (CH, pyrrolyl), 122.3 (2 x CH, d, *J* =

7.6 Hz, 4-fluorophenyl), 118.6 (C, pyrrolyl), 115.8 (2 x CH, d, *J* = 22.7 Hz, 4-fluorophenyl), 112.7 (C, pyrrolyl), 106.5 (CH, pyrrolyl).

***tert*-Butyl (*S*)-(1-((3-chloro-2-((4-fluorophenyl)carbamoyl)-1*H*-pyrrol-1-yl)amino)-1-oxopropan-2-yl)carbamate (10d)**

To a solution of 1-amino-3-chloro-*N*-(4-fluorophenyl)-1*H*-pyrrole-2-carboxamide (**15d**, 624.2 mg, 2.46 mmol) in THF (5 mL) was added *N*-(*tert*-butoxycarbonyl)-L-alanine (651.73 mg, 3.44 mmol) and 1-(3-dimethylaminopropyl)-3-ethylcarbodiimide hydrochloride (660 mg, 3.44 mmol) at room temperature. After stirring overnight, the reaction mixture was extracted with EtOAc. The organic layer was dried over anhydrous $MgSO_4$, filtered and evaporated. The resulting crude residue was purified by column chromatography on silica gel (EtOAc/*n*-hexane 1:2) to give *tert*-butyl (*S*)-(1-((3-chloro-2-((4-fluorophenyl)carbamoyl)-1*H*-pyrrol-1-yl)amino)-1-oxopropan-2-yl)carbamate (**10d**, 957.9 mg, 92%) as a white solid.

mp. 153.0-154.8 °C; IR (KBr): ν 1679, 1649, 1611, 1528, 1422, 1323, 1160, 829 cm^{-1}; LC/MS: R_t = 3.03 mins, m/z (ES+) = 425 (M+H for $C_{19}H_{22}ClFN_4O_4$); ^1H NMR (300 MHz, $CDCl_3$) δ 10.31 (br, 1H, 4-fluorophenyl-N*H*-CO-), 8.35 (s, 1H, -N*H*-pyrrolyl), 7.50 (q, *J* = 4.8 Hz and *J* = 9.0 Hz, 2H, 4-fluorophenyl), 7.01 (t, *J* = 8.6 Hz, 2H, 4-fluorophenyl), 6.95 (d, *J* = 1.1 Hz, 1H, pyrrolyl), 6.18 (d, *J* = 1.3 Hz, 1H, pyrrolyl), 5.17 (br, 1H, Boc-N*H*-), 4.41 (br, 1H, CH_3-C*H*(NH-Boc)-CO-), 1.44 (s, 9H, *t*-butyl), 1.41 (d, *J* = 3.0 Hz, CH_3-CH(NH-Boc)-CO-); ^{13}C NMR (125 MHz, $CDCl_3$) δ 173.1 (CO), 159.6 (C, d, *J* = 244.4 Hz, 4-fluorophenyl), 157.4 (CO), 155.6 (CO), 133.3 (C, d, *J* = 2.5 Hz, 4-fluorophenyl), 126.9 (CH, pyrrolyl), 122.2 (2 x CH, d, *J* = 7.6 Hz, 4-fluorophenyl), 119.6 (C, pyrrolyl), 115.7 (2 x CH, d, *J* = 22.7 Hz, 4-fluorophenyl),

113.9 (C, pyrrolyl), 107.8 (CH, pyrrolyl), 80.5 (C), 48.9 (CH), 28.3 (3 x CH_3), 17.8 (CH_3).

***tert*-Butyl (*S*,*Z*)-(1-(5-chloro-4-((4-fluorophenyl)imino)-4*H*-pyrrolo[1,2-*d*][1,3,4]oxadiazin-2-yl)ethyl)carbamate (11d) and *tert*-butyl (*S*)-(1-(5-chloro-3-(4-fluorophenyl)-4-oxo-3,4-dihydropyrrolo[2,1-*f*][1,2,4]triazin-2-yl)ethyl)carbamate (12d)**

To a solution of triphenylphosphine (123.46 mg, 0.47 mmol) in CH_2Cl_2 (0.5 mL) was added bromine (0.02 mL, 0.47 mmol) at 0 °C. The mixture was stirred at room temperature for 15 min. Then, a solution of aminopyrrolocarbamate (**10d**, 100 mg, 0.23 mmol) in CH_2Cl_2 (0.5 mL) and triethylamine (0.16 mL, 1.14 mmol) were added at 0 °C. After complete addition, the reaction mixture was stirred at 0 °C for 5 min. Then the reaction mixture was extracted with CH_2Cl_2. The organic layer was dried over anhydrous $MgSO_4$, filtered and evaporated. The resulting crude residue was purified by column chromatography on silica gel (EtOAc/*n*-hexane 1:10) to give iminooxadiazinone (**11d**, 42 mg, 41%) and triazinone (**12d**, 19 mg, 19%) as a white solid.

11d: mp. 140.0-140.8 °C; IR (ATR): ν 1677, 1598, 1522, 1500, 1463, 1325, 1308, 1211, 1160, 1068, 1026, 966, 841, 763 cm^{-1}; ^{1}H NMR (300 MHz, $CDCl_3$) δ 7.14 (q, *J* = 4.8 Hz and *J* = 8.7 Hz, 2H, 4-fluorophenyl), 6.99-7.06 (m, 3H, 4-fluorophenyl and pyrrolyl), 6.37 (d, *J* = 1.1 Hz, pyrrolyl), 4.84 (br, 1H, Boc-N*H*-), 4.52 (br, 1H, CH_3-C*H*(NH-Boc)-C-), 1.43 (s, 9H, *t*-butyl), 1.39 (d, *J* = 6.9 Hz, CH_3-CH(NH-Boc)-C-); ^{13}C NMR (125 MHz, $CDCl_3$) δ 159.9 (C, d, *J* = 243.9 Hz, 4-fluorophenyl), 155.4 (C), 154.7 (CO), 140.0 (CH, d, *J* = 2.9 Hz, 4-fluorophenyl), 136.5 (C=N), 124.8 (CH, d, *J*

= 8.3 Hz, 4-fluorophenyl), 124.6 (CH, d, *J* = 8.1 Hz, 4-fluorophenyl), 120.3 (CH, pyrrolyl), 120.2, 115.5 (CH, d, *J* = 11.6 Hz, 4-fluorophenyl), 115.4 (CH, d, *J* = 11.3 Hz, 4-fluorophenyl), 114.6 (C, pyrrolyl), 111.0 (CH, pyrrolyl), 110.2 (C, pyrrolyl), 80.4 (C), 47.5 (CH), 29.7, 28.3 (3 x CH_3), 18.9 (CH_3), 18.8; ^{19}F-NMR (470 MHz) δ -119.46 ppm; HRMS (EI) calcd for $C_{19}H_{20}ClFN_4O_3$ 406.1208, found 406.1208.

12d: mp. 241.7-242.0 °C; IR (ATR): ν 1692, 1620, 1504, 1461, 1401, 1335, 1221, 1154, 1043, 833, 772, 762 cm^{-1}; ^{1}H NMR (300 MHz, $CDCl_3$) δ 7.39-7.40 (m, 1H, 4-fluorophenyl), 7.21-7.29 (m, 4H, 4-fluorophenyl), 6.50 (d, *J* = 1.1 Hz, 1H, pyrrolyl), 5.08 (d, *J* = 3.5 Hz, Boc-N*H*-), 4.46 (qui, *J* = 7.1 Hz, CH_3-C*H*(NH-Boc)-C-), 1.42 (s, 9H, *t*-butyl), 1.26-1.28 (m, 3H, CH_3-CH(NH-Boc)-C-); ^{13}C NMR (125 MHz, $CDCl_3$) δ 163.0 (C, d, *J* = 251.0 Hz, 4-fluorophenyl), 154.7 (CO), 153.8 (CO), 151.7 (C), 131.2 (CH, d, *J* = 9.0 Hz, 4-fluorophenyl), 131.1 (CH, d, *J* = 9.2 Hz, 4-fluorophenyl), 130.0 (CH, d, *J* = 3.0 Hz, 4-fluorophenyl), 120.1 (CH, pyrrolyl), 117.3 (CH, d, *J* = 23.7 Hz, 4-fluorophenyl), 116.6 (d, *J* = 23.2 Hz), 114.2 (C, pyrrolyl), 113.9 (C, pyrrolyl), 111.5 (CH, pyrrolyl), 80.2 (C), 46.7 (CH), 29.7, 28.3 (3 x CH_3), 20.3 (CH_3); ^{19}F-NMR (470 MHz) δ -111.80 ppm; HRMS (EI) calcd for $C_{19}H_{20}ClFN_4O_3$ 406.1208, found 406.1206.

3-Chloro-*N*-(4-methoxyphenyl)-1*H*-pyrrole-2-carboxamide (14e)

To a solution of 3-chloro-1*H*-pyrrole-2-carboxylic acid (**13**, 615.63 mg, 4.23 mmol) in dry CH_2Cl_2 (20 mL) was added oxalyl chloride (0.54 mL, 6.35 mmol) and DMF (2 drops) at 0 °C. The reaction was stirred at 70 °C for 5 h. The solvent was removed under reduced pressure, the resulting crude residue was then dried under high vaccum and used to the next reaction without further purification.

To a solution of 3-chloro-1*H*-pyrrole-2-carbonyl chloride (700 mg, 4.23 mmol) in 1,4-

dioxane (7 mL) was added *p*-Anisidine (657.13 mg, 5.34 mmol) and DIPEA (2.23 mL, 12.81 mmol) at 0 °C. The mixture was stirred at 60 °C for 4 h, a solvent was concentrated under reduced pressure. The mixture was diluted with EtOAc and H_2O, and the layers were separated. The organic layer was dried over anhydrous $MgSO_4$, filtered and evaporated. The resulting crude residue was purified by column chromatography on silica gel (EtOAc/*n*-hexane 1:5) to give 3-chloro-*N*-(4-methoxyphenyl)-1*H*-pyrrole-2-carboxamide (**14e**, 941.5 mg, 85%) as a white solid.

mp. 146.0-150.0 °C; ^{1}H NMR (300 MHz, $CDCl_3$) δ 10.17 (br, 1H, 4-methoxyphenyl-N*H*-CO-), 8.48 (s, 1H, pyrrolyl N*H*), 7.54 (d, *J* = 3.8 Hz, 2H, 4-methoxyphenyl), 6.88-6.93 (m, 3H, 4-methoxyphenyl and pyrrolyl), 6.26 (t, *J* = 2.6 Hz, 1H, pyrrolyl), 3.82 (s, 3H, 4-methoxyphpenyl); ^{13}C NMR (125 MHz, $CDCl_3$) δ 157.9 (CO), 156.6 (C, 4-methoxyphenyl), 130.6 (C, 4-methoxyphenyl), 122.1 (2 x CH, 4-methoxyphenyl), 121.3 (C, pyrrolyl), 120.9 (CH, pyrrolyl), 114.3 (2 x CH, 4-methoxyphpenyl), 112.5 (C, pyrrolyl), 111.0 (CH, pyrrolyl), 55.5 (OCH_3, 4-methoxyphenyl).

1-Amino-3-chloro-*N*-(4-methoxyphenyl)-1*H*-pyrrole-2-carboxamide (15e)

An aqueous solution of sodium hydroxide (28 wt %, 10.58 mL, 74.06 mmol) was added to ammonium chloride (1.19 g, 22.22 mmol) at 0 °C. Then, an aqueous solution of ammonium hydroxide (25 wt %, 10.38 mL, 74.06 mmol), aliquat 336 (0.17 mL, 0.37 mmol), a solution of 3-chloro-*N*-(4-methoxyphenyl)-1*H*-pyrrole-2-carboxamide (928.4 mg, 3.70 mmol) in *tert*-butylmethyl ether/diethyl ether (1:1, 10 mL) and sodium hypochlorite (10%, 24.8 mL, 37.03 mmol) was added at 0 °C. After stirring at room temperature overnight, the reaction mixture was extracted with EtOAc. The organic layer was dried over $MgSO_4$, filtered and evaporated. The

residue was purified by column chromatography on silica gel (EtOAc/*n*-hexane, 1:10) to give 1-amino-3-chloro-*N*-(4-methoxyphenyl)-1*H*-pyrrole-2-carboxamide (**15e**, 762.1 mg, 77%) as a yellow solid.

mp. 160.5-161.7 °C; ^{1}H NMR (300 MHz, $CDCl_3$) δ 8.41 (br, 1H, 4-methoxyphenyl-N*H*-CO-), 7.48 (d, *J* = 3.7 Hz, 2H, 4-methoxyphenyl), 6.88-6.92 (m, 3H, 4-methoxyphenyl and pyrrolyl), 6.07 (d, *J* = 1.1 Hz, 1H, pyrrolyl), 5.92 (s, 2H, NH_2-pyrrolyl), 3.81 (s, 3H, 4-methoxyphenyl); ^{13}C NMR (125 MHz, $CDCl_3$) δ 158.9 (CO), 156.8 (C, 4-methoxyphenyl), 130.3 (C, 4-methoxyphenyl), 126.2 (CH, pyrrolyl), 122.4 (2 x CH, 4-methoxyphenyl), 118.8 (C, pyrrolyl), 114.3 (2 x CH, 4-methoxyphenyl), 112.4 (C, pyrrolyl), 106.3 (CH, pyrrolyl), 55.5 (OCH_3, 4-methoxyphenyl).

***tert*-Butyl (*S*)-(1-((3-chloro-2-((4-methoxyphenyl)carbamoyl)-1*H*-pyrrol-1-yl)amino)-1-oxopropan-2-yl)carbamate (10e)**

To a solution of 1-amino-3-chloro-*N*-(4-methoxyphenyl)-1*H*-pyrrole-2-carboxamide (**15e**, 742.8 mg, 2.80 mmol) in THF (5 mL) was added *N*-(*tert*-butoxycarbonyl)-L-alanine (740.5 mg, 3.91 mmol) and 1-(3-dimethylaminopropyl)-3-ethylcarbodiimide hydrochloride (750.28 mg, 3.91 mmol) at room temperature. After stirring overnight, the reaction mixture was extracted with EtOAc. The organic layer was dried over anhydrous $MgSO_4$, filtered and evaporated. The resulting crude residue was purified by column chromatography on silica gel (EtOAc/*n*-hexane 1:5) to give *tert*-butyl (*S*)-(1-((3-chloro-2-((4-methoxyphenyl)carbamoyl)-1*H*-pyrrol-1-yl)amino)-1-oxopropan-2-yl)carbamate (**10e**, 971.5 mg, 80%) as a white solid.

mp. 137.0-138.0 °C ℃; IR (KBr): ν 1680, 1640, 1600, 1514, 1458, 1423, 1248, 1172, 1031, 826 cm^{-1}; LC/MS: R_t = 2.96 mins, m/z (ES+) = 437 (M+H for

$C_{20}H_{25}ClN_4O_5$); ^{1}H NMR (300 MHz, $CDCl_3$) δ 10.36 (br, 1H, 4-methoxyphenyl-N*H*-CO-), 8.29 (s, 1H, -N*H*-pyrrolyl), 7.44 (d, *J* = 3.7 Hz, 2H, 4-methoxyphenyl), 7.00 (d, *J* = 1.3 Hz, 1H, pyrrolyl), 6.87 (d, *J* = 3.8 Hz, 2H, 4-methoxyphenyl), 6.19 (d, *J* = 1.3 Hz, 2H, pyrrolyl), 5.13 (d, *J* = 2.9 Hz, 1H, Boc-N*H*-), 4.40 (t, *J* = 6.6 Hz, 1H, CH_3-C*H*(NH-Boc)-CO-), 3.80 (s, 3H, 4-methoxyphenyl), 1.45 (s, 9H, *t*-butyl), 1.43 (d, *J* = 3.0 Hz, 3H, CH_3-CH(NH-Boc)-CO-); ^{13}C NMR (125 MHz, $CDCl_3$) δ 172.8 (CO), 157.6 (CO), 156.8 (CO), 155.6 (C, 4-methoxyphenyl), 130.2 (C, 4-methoxyphenyl), 126.5 (CH, pyrrolyl), 122.4 (2 x CH, 4-methoxyphenyl), 119.1 (C, pyrrolyl), 114.2 (2 x CH, 4-methoxyphenyl), 113.6 (C, pyrrolyl), 107.6 (CH, pyrrolyl), 80.6 (C), 55.5 (OCH_3, 4-methoxyphenyl), 49.1 (CH), 28.3 (3 x CH_3), 17.7 (CH_3).

***tert*-Butyl (*S*,*Z*)-(1-(5-chloro-4-((4-methoxyphenyl)imino)-4*H*-pyrrolo[1,2-*d*][1,3,4]oxadiazin-2-yl)ethyl)carbamate (11e) and *tert*-butyl (*S*)-(1-(5-chloro-3-(4-methoxyphenyl)-4-oxo-3,4-dihydropyrrolo[2,1-*f*][1,2,4]triazin-2-yl)ethyl)carbamate (12e)**

To a solution of dichlorotriphenylphosphorane (229 mg, 0.69 mmol) in CH_2Cl_2 (0.5 mL) was added aminopyrrolocarbamates (**10e**, 100 mg, 0.23 mmol) and triethylamine (0.16 mL, 1.14 mmol) at 0 °C. After complete addition, the reaction mixture was stirred at 0 °C for 5 min. Then, the reaction mixture was extracted with CH_2Cl_2. The organic layer was dried over anhydrous $MgSO_4$, filtered and evaporated. The resulting crude residue was purified by column chromatography on silica gel (EtOAc/*n*-hexane 1:10) to give iminooxadiazinone (**11e**, 19 mg, 20%) and triazinone (**12e**, 75 mg, 78%) as a white solid.

11e: mp. 153-154 °C; IR (KBr): ν 1689, 1649, 1601, 1522, 1505, 1313, 1245, 1161,

951, 803, 741 cm^{-1}; ^{1}H NMR (300 MHz, $CDCl_3$) δ 7.20 (d, *J* = 8.8 Hz, 2H, 4-methoxyphenyl), 7.04 (d, *J* = 3.0 Hz, 1H, pyrrolyl), 6.88 (d, *J* = 8.9 Hz, 2H, 4-methoxyphenyl), 6.36 (d, *J* = 3.0 Hz, 1H, pyrrolyl), 4.87 (br, 1H, Boc-N*H*-), 4.55 (br, 1H, CH_3-C*H*(NH-Boc)-C-), 3.82 (s, 3H, 4-methoxyphenyl), 1.44 (s, 10H, *t*-butyl and C*H*3-CH(NH-Boc)-C-), 1.41 (s, 2H, C*H*3-CH(NH-Boc)-C-); δ ^{13}C NMR (125 MHz, $CDCl_3$) δ 156.9, 154.8, 136.8, 125.0, 120.0, 114.0, 113.9, 110.8, 110.6, 80.4, 55.4, 47.5, 28.3, 19.1; HRMS (EI) calcd for $C_{20}H_{23}ClN_4O_4$ 418.1408, found 418.1418.

12e: mp. 89-90 °C; IR (KBr): ν 1702, 1623, 1511, 1404, 1336, 1250, 1168, 1029, 798 cm^{-1}; ^{1}H NMR (300 MHz, $CDCl_3$) δ 7.23 – 7.43 (m, 2H), 7.16 – 7.19 (m, 1H), 6.99 – 7.06 (m, 2H), 6.48 (d, *J* = 2.5 Hz, 1H, pyrrolyl), 5.16 (br, 1H, Boc-N*H*-), 4.52 (br, 1H, CH_3-C*H*(NH-Boc)-C-), 3.85 (s, 3H, 4-methoxyphenyl), 1.42 (s, 9H, *t*-butyl), 1.26 (d, *J* = 6.6 Hz, 3H, C*H*3-CH(NH-Boc)-C-); ^{13}C NMR (125 MHz, $CDCl_3$) δ 160.3, 154.7, 154.0, 152.1, 130.3 (d, *J* = 9.4 Hz), 126.4, 119.8, 115.2, 114.9, 114.1, 113.8, 111.3, 80.0, 55.5, 46.7, 28.4, 20.5; HRMS (EI) calcd for $C_{20}H_{23}ClN_4O_4$ 418.1408, found 418.1407.

3-chloro-*N*-(4-cyanophenyl)-1*H*-pyrrole-2-carboxamide (14f)

To a solution of 3-chloro-1*H*-pyrrole-2-carboxylic acid (**13**, 585.07 mg, 4.02 mmol) in dry CH_2Cl_2 (20 mL) was added oxalyl chloride (0.52 mL, 6.03 mmol) and DMF (2 drops) at 0 °C. The reaction was stirred at 70 °C for 4 h. The solvent was removed under reduced pressure, the resulting crude residue was then dried under high vaccum and used to the next reaction without further purification.

To a solution of 3-chloro-1*H*-pyrrole-2-carbonyl chloride (584.7 mg, 4.02 mmol) in 1,4-dioxane (7 mL) was 4-aminobenzonitrile (593.28 mg, 5.02 mmol) and DIPEA

(2.10 mL, 12.05 mmol) at 0 °C. The mixture was stirred at 80 °C for 16 h, a solvent was concentrated under reduced pressure. The mixture was diluted with EtOAc and H_2O, and the layers were separated. The organic layer was dried over anhydrous $MgSO_4$, filtered and evaporated. The resulting crude residue was purified by column chromatography on silica gel (EtOAc/*n*-hexane 1:2) to give 3-chloro-*N*-(4-cyanophenyl)-1*H*-pyrrole-2-carboxamide (**14f**, 699 mg, 71%) as a yellow solid.

mp. 216.0-216.8 °C; ^{1}H NMR (300 MHz, DMSO) δ 12.04 (br, 1H, 4-cyanophenyl-N*H*-CO-), 9.87 (s, 1H, pyrrolyl N*H*), 7.84 (q, *J* = 8.7 Hz and *J* = 18.0 Hz, 4H, 4-cyanophenyl), 7.08 (t, *J* = 2.9 Hz, 1H, pyrrolyl), 6.29 (t, *J* = 2.4 Hz, 1H, pyrrolyl); ^{13}C NMR (125 MHz, DMSO) δ 158.6, 143.5, 133.7, 122.7, 121.7, 120.2, 119.5, 114.8, 110.8, 105.6.

1-amino-3-chloro-*N*-(4-cyanophenyl)-1*H*-pyrrole-2-carboxamide (15f)

An aqueous solution of sodium hydroxide (28 wt %, 8.13 mL, 56.90 mmol) was added to ammonium chloride (913.04 mg, 17.07 mmol) at 0 °C. Then, an aqueous solution of ammonium hydroxide (25 wt %, 7.98 mL, 56.90 mmol), aliquat 336 (0.13 mL, 0.28 mmol), a solution of 3-chloro-*N*-(4-cyanophenyl)-1*H*-pyrrole-2-carboxamide (699 mg, 2.84 mmol,) in *tert*-butylmethyl ether/diethyl ether/EtOAc (1:1:1, 15 mL) and sodium hypochlorite (10%, 19.1 mL, 28.45 mmol) was added at 0 °C. After stirring at room temperature overnight the reaction mixture was extracted with EtOAc. The organic layer was dried over $MgSO_4$, filtered and evaporated. The crude compound was used to the next reaction without further purification.

mp. 192 °C; ^{1}H NMR (300 MHz, $CDCl_3$) δ 8.77 (br, 1H, 4-cyanophenyl-N*H*-CO-), 7.74 (d, *J* = 8.7 Hz, 2H, 4-cyanophenyl), 7.65 (d, *J* = 9.0 Hz, 2H, 4-cyanophenyl),

6.95 (d, *J* = 3.0 Hz, 1H, pyrrolyl), 6.11 (d, *J* = 3.0 Hz, 1H, pyrrolyl), 5.84 (s, 2H, NH_2-pyrrolyl).

***tert*-Butyl (*S*)-(1-((3-chloro-2-((4-cyanophenyl)carbamoyl)-1*H*-pyrrol-1-yl)amino)-1-oxopropan-2-yl)carbamate (10f)**

To a solution of 1-amino-3-chloro-*N*-(4-cyanophenyl)-*1H*-pyrrole-2-carboxamide (**15f**, 229 mg, 0.88 mmol) in THF (5 mL) was added *N*-(*tert*-butoxycarbonyl)-L-alanine (235.75 mg, 1.23 mmol) and 1-(3-dimethylaminopropyl)-3-ethylcarbodiimide hydrochloride (232.69 mg, 1.23 mmol) at room temperature. After stirring overnight, the reaction mixture was extracted with EtOAc. The organic layer was dried over anhydrous $MgSO_4$, filtered and evaporated. The resulting crude residue was purified by column chromatography on silica gel (EtOAc/*n*-hexane 1:5) to give *tert*-butyl (*S*)-(1-((3-chloro-2-((4-cyanophenyl)carbamoyl)-1*H*-pyrrol-1-yl)amino)-1-oxopropan-2-yl)carbamate (**10f**, 135.9 mg, 36%) as colorless solid.

mp. 137 °C; IR (KBr): ν 2227, 1682, 1654, 1591, 1526, 1418, 1322, 1256, 1173, 834 cm^{-1}; LC/MS: R_t = 2.92 mins, m/z (ES+) = 432 (M+H for $C_{20}H_{22}ClN_5O_4$); ^{1}H NMR (300 MHz, $CDCl_3$) δ 10.13 (s, 1H, 4-cyanophenyl-N*H*-CO-), 8.57 (s, 1H, pyrrolyl-N*H*-), 7.66-7.72 (m, 2H, 4-cyanophenyl), 7.60-7.64 (m, 2H, 4-cyanophenyl), 6.98 (d, *J* = 3.0 Hz, 1H, pyrrolyl), 6.23 (d, *J* = 3.3 Hz, 1H, pyrrolyl), 5.09 (br, 1H, Boc-N*H*-), 4.41 (t, *J* = 6.9 Hz, 1H, CH_3-C*H*(NH-Boc)-CO-), 1.47 (s, 9H, *t*-butyl), 1.43 (d, *J* = 7.2 Hz, 3H, CH_3-CH(NH-Boc)-CO-); ^{13}C NMR (75 MHz, $CDCl_3$) δ 173.0, 167.0, 157.3, 155.8, 141.5, 133.3, 127.9, 119.9, 119.0, 118.8, 114.7, 108.2, 108.1, 107.4, 80.8, 48.8, 28.4, 28.3, 17.3.

***tert*-Butyl (*S*,*Z*)-(1-(5-chloro-4-((4-cyanophenyl)imino)-4*H*-pyrrolo[1,2-*d*][1,3,4]oxadiazin-2-yl)ethyl)carbamate (11f) and *tert*-butyl (*S*)-(1-(5-chloro-3-(4-cyanophenyl)-4-oxo-3,4-dihydropyrrolo[2,1-*f*][1,2,4]triazin-2-yl)ethyl)carbamate (12f):**

To a solution of dichlorotriphenylphosphorane (93 mg, 0.28 mmol) in CH_2Cl_2 (0.5 mL) was added aminopyrrolocarbamate (**10f**, 40 mg, 0.09 mmol) at 0 °C. Then, triethylamine (0.07 mL, 0.46 mmol) were added at 0 °C. After complete addition, the reaction mixture was stirred at 0 °C for 5 min. Then, the reaction mixture was extracted with CH_2Cl_2. The organic layer was dried over anhydrous $MgSO_4$, filtered and evaporated. The resulting crude residue was purified by column chromatography on silica gel (EtOAc/*n*-hexane 1:5) to give iminooxadiazinone (**11f**, 17 mg, 43%) and triazinone (**12f**, 16 mg, 41%) as a white solid.

11f: mp. 155.8-156.0 °C; IR (KBr): ν 2230, 1697, 1678, 1599, 1524, 1462, 1411, 1249, 1163, 970, 854, 764 cm^{-1}; ^{1}H NMR (300 MHz, $CDCl_3$) δ 7.63 (d, 2H, *J* = 8.4, 4-cyanophenyl), 7.18 (d, *J* = 8.4 Hz, 2H, 4-cyanophenyl), 7.13 (d, *J* = 2.7 Hz, 1H, pyrrolyl), 6.43 (d, *J* = 2.7 Hz, 1H, pyrrolyl), 4.76 (br, 1H, Boc-N*H*-), 4.50 (br, 1H, CH_3-C*H*(NH-Boc)-C-), 1.43 (s, 9H, *t*-butyl), 1.38 (d, 3H, *J* = 6.9 Hz, CH_3-CH(NH-Boc)-C-); ^{13}C NMR (125 MHz, $CDCl_3$) δ; 154.6, 148.6, 138.0, 132.9, 123.4, 121.1, 119.2, 115.6, 111.4, 109.7, 107.6, 80.6, 47.4, 28.3, 18.8; HRMS (EI) calcd for $C_{20}H_{20}ClN_5O_3$ 413.1255, found 413.1251.

12f: mp. 182.7-182.9 °C; IR (KBr): ν 2233, 1699, 1678, 1624, 1507, 1401, 1338, 1236, 1155, 1055, 770 cm^{-1}; ^{1}H NMR (300 MHz, $CDCl_3$) δ 7.85 (t, 2H, *J* = 8.6 Hz, 4-

cyanophenyl), 7.60 (d, *J* = 7.8 Hz, 1H, 4-cyanophenyl), 7.43 (d, *J* = 8.1 Hz, 1H, 4-cyanophenyl), 7.32 (d, *J* = 2.7 Hz, 1H, pyrrolyl), 6.53 (d, *J* = 2.7 Hz, 1H, pyrrolyl), 5.01 (d, *J* = 8.4 Hz, 1H, Boc-N*H*-), 4.39 (qui, *J* = 7.5 Hz, 1H, CH_3-C*H*(NH-Boc)-C-), 1.41 (s, 9H, *t*-butyl), 1.27 (d, 3H, *J* = 6.9 Hz, CH_3-CH(NH-Boc)-C-); ^{13}C NMR (125 MHz, $CDCl_3$) δ; 154.7, 153.3, 150.8, 138.4, 133.9, 133.3, 130.8, 130.4, 120.6, 117.7, 114.9, 113.9, 113.6, 111.8, 80.5, 46.7, 28.3, 20.1; HRMS (EI) calcd for $C_{20}H_{20}ClN_5O_3$ 413.1255, found 413.1254.

3-Chloro-*N*-(4-methoxybenzyl)-1*H*-pyrrole-2-carboxamide (14g)

To a solution of 3-chloro-1*H*-pyrrole-2-carboxylic acid (700 mg, 4.81 mmol) in dry CH_2Cl_2 (20 mL) was added oxalyl chloride (0.62 mL, 7.21 mmol) and DMF (2 drops) at 0 °C. The reaction was stirred at 70 °C for 4 h. The solvent was removed under reduced pressure, the resulting crude residue was then dried under high vaccum and used to the next reaction without further purification.

To a solution of 3-chloro-1*H*-pyrrole-2-carbonyl chloride (788.79 mg, 4.81 mmol) in 1,4-dioxane (7 mL) was added 4-methoxybenzylamine (0.8 mL, 6.01 mmol) and DIPEA (2.51 mL, 14.43 mmol) at 0 °C. The reaction mixture was stirred at 65 °C for 3 h, a solvent was concentrated under reduced pressure. The mixture was diluted with EtOAc and H_2O, and the layers were separated. The organic layer was dried over anhydrous $MgSO_4$, filtered and evaporated. The resulting crude residue was purified by column chromatography on silica gel (EtOAc/*n*-hexane 1:5) to give 3-chloro-*N*-(4-methoxybenzyl)-1*H*-pyrrole-2-carboxamide (**14g**, 1.09 g, 86%) as a brown solid.

mp. 101.0-102.0 °C; ^{1}H NMR (300 MHz, $CDCl_3$) δ 10.02 (br, 1H, pyrrolyl N*H*), 7.30 (d, *J* = 3.8 Hz, 2H, 4-methoxybenzyl), 7.07 (s, 1H, 4-methoxybenzyl-N*H*-CO-), 6.91 (d, *J*

= 3.3 Hz, 2H, 4-methoxybenzyl), 6.84 (s, 1H, pyrrolyl), 6.22 (s, 1H, pyrrolyl), 4.62 (t, *J* = 2.3 Hz, 2H, CH_3O-Ph-CH_2-NH-), 3.83 (s, 3H, CH_3O-Ph-CH_2-NH-); ^{13}C NMR (125 MHz, $CDCl_3$) δ 160.0 (CO), 159.1 (C, 4-methoxybenzyl), 130.2 (C, 4-methoxybenzyl), 128.9 (2 x CH), 121.2 (C, pyrrolyl), 120.4 (CH, pyrrolyl), 114.2 (2 x CH, 4-methoxybenzyl), 112.5 (C, pyrrolyl), 110.8 (CH, pyrrolyl), 55.3 (OCH_3, 4-methoxybenzyl), 42.9 (CH_2, 4-methoxybenzyl).

1-Amino-3-chloro-*N*-(4-methoxybenzyl)-1*H*-pyrrole-2-carboxamide (15g)

An aqueous solution of sodium hydroxide (28 wt %, 11.64 mL, 81.50 mmol) was added to ammonium chloride (1.3 g, 24.45 mmol) at 0 °C. Then, an aqueous solution of ammonium hydroxide (25 wt %, 11.42mL, 81.50 mmol), aliquat 336 (0.17 mL, 0.41 mmol), a solution of 3-chloro-*N*-(4-methoxybenzyl)-1*H*-pyrrole-2-carboxamide (**14g**, 1.0786 g, 4.07 mmol) in *tert*-butylmethyl ether/diethyl ether/EtOAc (1:1:1, 15 mL) and sodium hypochlorite (10%, 24.6 mL, 40.75 mmol) was added at 0 °C. After stirring at room temperature overnight, the reaction mixture was extracted with EtOAc. The organic layer was dried over $MgSO_4$, filtered and evaporated. The residue was purified by column chromatography on silica gel (EtOAc/*n*-hexane, 1:5) to give 1-amino-3-chloro-*N*-(4-methoxybenzyl)-1*H*-pyrrole-2-carboxamide (**15g**, 1.06 g, 94%) as a yellow solid.

mp. 93.6-94.8 °C; ^{1}H NMR (300 MHz, $CDCl_3$) δ 7.28-7.32 (m, 2H, 4-methoxybenzyl), 7.07 (br, 1H, 4-methoxybenzyl-N*H*-CO-), 6.91 (d, *J* = 3.4 Hz, 2H, 4-methoxybenzyl), 6.86 (d, *J* = 1.3 Hz, 1H, pyrrolyl), 6.03 (d, *J* = 1.3 Hz, 2H, pyrrolyl), 5.95 (s, 2H, NH_2-pyrrolyl), 4.58 (d, *J* = 3.5 Hz, 2H, CH_3O-Ph-CH_2-NH-), 3.83 (s, 3H, CH_3O-Ph-CH_2-NH-); ^{13}C NMR (125 MHz, $CDCl_3$) δ 160.9 (CO), 159.1 (C, 4-methoxybenzyl), 130.0

(C, 4-methoxybenzyl), 128.9 (2 x CH, 4-methoxybenzyl), 125.5 (CH, pyrrolyl), 118.6 (C, pyrrolyl), 114.2 (2 x CH, 4-methoxybenzyl), 112.3 (C, pyrrolyl), 106.1 (CH, pyrrolyl), 55.3 (OCH_3, 4-methoxybenzyl), 42.7 (CH_2, 4-methoxybenzyl).

***tert*-Butyl (*S*)-(1-((3-chloro-2-((4-methoxybenzyl)carbamoyl)-1*H*-pyrrol-1-yl)amino)-1-oxopropan-2-yl)carbamate (10g)**

To a solution of 1-amino-3-chloro-*N*-(4-methoxybenzyl)-1*H*-pyrrole-2-carboxamide (**15g**, 1.06 g, 3.78 mmol) in THF (5 mL) was added *N*-(*tert*-butoxycarbonyl)-L-alanine (1.02 g, 5.29 mmol) and 1-(3-dimethylaminopropyl)-3-ethylcarbodiimide hydrochloride (1.00 g, 5.29 mmol) at room temperature. After stirring overnight, the reaction mixture was extracted with EtOAc. The organic layer was dried over anhydrous $MgSO_4$, filtered and evaporated. The resulting crude residue was purified by column chromatography on silica gel (EtOAc/*n*-hexane, 1:5) to give *tert*-butyl (*S*)-(1-((3-chloro-2-((4-methoxybenzyl)carbamoyl)-1*H*-pyrrol-1-yl)amino)-1-oxopropan-2-yl)carbamate (**10g**, 1.45 g, 85%) as a white solid.

mp. 142.0-143.0 °C; IR (KBr): ν 1679, 1625, 1551, 1515, 1485, 1454, 1366, 1250, 1174, 1039 cm^{-1}; LC/MS: R_t = 2.96 mins, m/z (ES+) = 451 (M+H for $C_{21}H_{27}ClN_4O_5$); ^1H NMR (300 MHz, $CDCl_3$) δ 10.47 (br, 1H, -N*H*-pyrrolyl), 7.23 (d, *J* = 3.5 Hz, 2H, 4-methoxybenzyl), 6.99 (d, *J* = 1.3 Hz, 1H, pyrrolyl), 6.86-6.88 (m, 3H, 4-methoxybenzyl-N*H*-CO- and 4-methoxybenzyl), 6.13 (d, *J* = 1.3 Hz, 1H, pyrrolyl), 5.15 (br, 1H, Boc-N*H*-), 4.50 (d, *J* = 2.3 Hz, 2H, CH_3O-Ph-CH_2-NH-), 4.40 (br, 1H, CH_3-C*H*(NH-Boc)-CO-), 3.80 (s, 3H, CH_3O-Ph-CH_2-NH-), 1.41-1.47 (m, 12H, *t*-butyl and CH_3-CH(NH-Boc)-CO-); ^{13}C NMR (125 MHz, $CDCl_3$) δ 172.8 (CO), 159.8 (CO), 159.1 (C, 4-methoxybenzyl), 155.4 (CO), 129.7 (C, 4-methoxybenzyl), 128.9 (2 x CH,

4-methoxybenzyl), 125.9 (CH, pyrrolyl), 118.7 (C, pyrrolyl), 114.2 (2 x CH, 4-methoxybenzyl), 113.5 (C, pyrrolyl), 107.4 (CH, pyrrolyl), 80.5 (C), 55.3 (OCH_3, 4-methoxybenzyl), 49.3 (CH), 42.8 (CH_2, 4-methoxybenzyl), 28.4 (3 x CH_3), 17.9 (CH_3).

tert-Butyl (_S_)-(1-(5-chloro-3-(4-methoxybenzyl)-4-oxo-3,4-dihydropyrrolo[2,1-_f_][1,2,4]triazin-2-yl)ethyl)carbamate (12g)

To a solution of triphenylphosphine (116.35 mg, 0.44 mmol) in CH_2Cl_2 (0.5 mL) was added bromine (0.02 mL, 0.44 mmol) at 0 °C. The mixture was stirred at room temperature for 15 min. Then, a solution of aminopyrrolocarbamate (**10g**, 100 mg, 0.22 mmol) in CH_2Cl_2 (0.5 mL) and triethylamine (0.15 mL, 1.11 mmol) were added at 0 °C. After complete addition, the reaction mixture was stirred at 0 °C for 5 min. Then, the reaction mixture was extracted with CH_2Cl_2. The organic layer was dried over anhydrous $MgSO_4$, filtered and evaporated. The resulting crude residue was purified by column chromatography on silica gel (EtOAc/_n_-hexane 1:2) to give triazinone (**12g**, 57.5 mg, 60%) as a white solid.

mp. 134.7-135.6 °C; IR (KBr): ν 1710, 1686, 1652, 1526, 1511, 1323, 1305, 1246, 1221, 1166, 1118, 1078, 1061, 1042, 1029, 951, 813, 737 cm^{-1}; ^1H NMR (300 MHz, $CDCl_3$) δ 7.37 (d, J = 8.4 Hz, 2H, 4-methoxybenzyl), 6.97 (d, J = 3.0 Hz, 1H, pyrrolyl), 6.88 (d, J = 8.4 Hz, 2H, 4-methoxybenzyl), 6.29 (d, J = 3.0 Hz, 1H, pyrrolyl), 4.92 (br, 1H, Boc-N_H_-), 4.73 (s, 2H, CH_3O-Ph-CH_2-NH-), 4.62 (br, 1H, CH_3-C_H_(NH-Boc)-C-), 3.80 (s, 3H, CH_3O-Ph-CH_2-NH-), 1.47 (s, 3H, CH_3-CH(NH-Boc)-C-), 1.46 (s, 9H, _t_-butyl); ^{13}C NMR (125 MHz, $CDCl_3$) δ 158.4 (C, 4-methoxybenzyl), 154.8 (CO), 154.7 (CO), 137.5 (C), 131.8 (C, 4-methoxybenzyl), 128.5 (2 x CH, 4-methoxybenzyl), 119.4 (CH, pyrrolyl), 113.8 (2 x CH, 4-methoxybenzyl), 113.4 (C,

pyrrolyl), 110.6 (CH, pyrrolyl), 110.4 (C, pyrrolyl), 80.4 (C), 55.3 (OCH_3, 4-methoxybenzyl), 48.8 (CH_2, 4-methoxybenzyl), 47.4 (CH), 29.7, 28.3 (3 x CH_3), 18.9 (CH_3); HRMS (EI) calcd for $C_{21}H_{25}ClN_4O_4$ 432.1564, found 432.1566.

3-Chloro-*N*-cyclopropyl-1*H*-pyrrole-2-carboxamide (14h)

To a solution of 3-chloro-1*H*-pyrrole-2-carboxylic acid (**13**, 585.07 mg, 4.02 mmol) in dry CH_2Cl_2 (20 mL) was added oxalyl chloride (0.52 mL, 6.03 mmol) and DMF (2 drops) at 0 °C. The reaction was stirred at 70 °C for 4 h. The solvent was removed under reduced pressure, the resulting crude residue was then dried under high vaccum and used to the next reaction without further purification.

To a solution of 3-chloro-1*H*-pyrrole-2-carbonyl chloride (700 mg, 4.27 mmol) in 1,4-dioxane (7 mL) was cyclopropylamine (0.37 mL, 5.34 mmol) and DIPEA (2.23 mL, 12.81 mmol) at 0 °C. The mixture was stirred at 60 °C for 5 h, a solvent was concentrated under reduced pressure. The mixture was diluted with EtOAc and H_2O, and the layers were separated. The organic layer was dried over anhydrous $MgSO_4$, filtered and evaporated. The resulting crude residue was purified by column chromatography on silica gel (EtOAc/*n*-hexane 1:2) to give 3-chloro-*N*-cyclopropyl-1*H*-pyrrole-2-carboxamide (**14h**, 667.9 mg, 85%) as a white solid.

mp. 115.4-115.7 °C; ^{1}H NMR (300 MHz, $CDCl_3$) δ 10.09 (br, 1H, pyrrolyl N*H*), 6.86 (br, 1H, cyclopropyl-N*H*-CO-), 6.83 (t, *J* = 2.7 Hz, 1H, pyrrolyl), 6.18 (d, *J* = 2.7 Hz, 1H, pyrrolyl), 2.87 (oct, *J* = 3.3 Hz and *J* = 6.9 Hz, 1H, cyclopropyl), 0.86 (q, *J* = 6.5 Hz and *J* = 12.8 Hz, 2H, cyclopropyl), 0.60-0.65 (m 2H, cyclopropyl); ^{13}C NMR (125 MHz, $CDCl_3$) δ 161.4 (CO), 121.2 (C, pyrrolyl), 120.4 (CH, pyrrolyl), 112.4 (C, pyrrolyl), 110.7 (CH, pyrrolyl), 22.5 (CH, cyclopropyl), 6.9 (2 x CH_2, cyclopropyl).

1-Amino-3-chloro-*N*-cyclopropyl-1*H*-pyrrole-2-carboxamide (15h)

An aqueous solution of sodium hydroxide (28 wt %, 10.16 mL, 71.11 mmol) was added to ammonium chloride (1.14 g, 21.33 mmol) at 0 °C. Then, an aqueous solution of ammonium hydroxide (25 wt %, 9.9 mL, 71.11 mmol), aliquat 336 (0.16 mL, 0.35 mmol), a solution of 3-chloro-*N*-cyclopropyl-1*H*-pyrrole-2-carboxamide (**14h**, 656.4 mg, 3.55 mmol) in *tert*-butylmethyl ether/diethyl ether (1:1, 10 mL) and sodium hypochlorite (10%, 24 mL, 35.55 mmol) was added at 0 °C. After stirring at room temperature for 3 h, the reaction mixture was extracted with EtOAc. The organic layer was dried over $MgSO_4$, filtered and evaporated. The residue was purified by column chromatography on silica gel (EtOAc/*n*-hexane 1:2) to give 1-amino-3-chloro-*N*-cyclopropyl-1*H*-pyrrole-2-carboxamide (**15h**, 321 mg, 45%) as a yellow solid.

mp. 141.7-142.0 °C; ^{1}H NMR (300 MHz, $CDCl_3$) δ 6.85 (br, 1H, cyclopropyl-N*H*-CO-), 6.81 (s, 1H, pyrrolyl), 5.97 (s, 1H, pyrrolyl), 5.33 (br, 2H, NH_2-pyrrolyl), 2.82 (s, 1H, cyclopropyl), 0.85 (d, J = 6.3 Hz, 2H, cyclopropyl), 0.60 (s, 2H, cyclopropyl); ^{13}C NMR (75 MHz, $CDCl_3$) δ 162.4, 125.5, 118.4, 112.2, 106.2, 106.0, 22.3, 22.2, 6.8.

***tert*-Butyl (*S*)-(1-((3-chloro-2-(cyclopropylcarbamoyl)-1*H*-pyrrol-1-yl)amino)-1-oxopropan-2-yl)carbamate (10h)**

To a solution of 1-amino-3-chloro-*N*-cyclopropyl-1*H*-pyrrole-2-carboxamide (**15h**, 52.1 mg, 0.26 mmol) in THF (5 mL) was added *N*-(*tert*-butoxycarbonyl)-L-alanine (69.14 mg, 0.37 mmol) and 1-(3-dimethylaminopropyl)-3-ethylcarbodiimide hydrochloride (70.05 mg, 0.37 mmol) at room temperature. After stirring overnight, the reaction mixture was extracted with EtOAc. The organic layer was dried over anhydrous $MgSO_4$, filtered and evaporated. The resulting crude residue was purified

by column chromatography on silica gel (EtOAc/*n*-hexane 1:2) to give *tert*-butyl (*S*)-(1-((3-chloro-2-(cyclopropylcarbamoyl)-1*H*-pyrrol-1-yl)amino)-1-oxopropan-2-yl)carbamate (**10h**, 80.4 mg, 83%) as a white solid.

mp. 139.7-140.9 °C; IR (KBr): ν 1678, 1635, 1526, 1456, 1367, 1252, 1163 cm^{-1};

LC/MS: R_t = 2.55 mins, m/z (ES+) = 371 (M+H for $C_{16}H_{23}ClN_4O_4$); ^{1}H NMR (300 MHz, $CDCl_3$) δ 10.55 (br, 1H, pyrrolyl-N*H*-), 6.96 (d, *J* = 3.0 Hz, 1H, pyrrolyl), 6.76 (br, 1H, cyclopropyl-N*H*-CO-), 6.10 (d, *J* = 3.0 Hz, 1H, pyrrolyl), 5.25 (br, 1H, Boc-N*H*-), 4.41 (br, 1H, CH_3-C*H*(NH-Boc)-CO-), 2.78 (oct, *J* = 3.5 Hz and *J* = 6.8 Hz, 1H, cyclopropyl), 1.45 (s, 9H, *t*-butyl), 1.42 (s, 3H, CH_3-CH(NH-Boc)-CO-), 0.82 (q, *J* = 6.0 Hz and *J* = 12.0 Hz, 2H, cyclopropyl), 0.55-0.60 (m, 2H, cyclopropyl); ^{13}C NMR (125 MHz, $CDCl_3$) δ 172.8 (CO), 161.3 (CO), 155.4 (CO), 125.9 (CH, pyrrolyl), 118.8 (C, pyrrolyl), 113.5 (C, pyrrolyl), 107.4 (CH, pyrrolyl), 80.3 (C), 49.2 (CH), 28.3 (3 x CH_3), 22.4 (CH, cyclopropyl), 18.1 (CH_3), 6.84 (CH_2), 6.80 (CH_2).

***tert*-Butyl (*S*)-(1-(5-chloro-3-(cyclopropylmethyl)-4-oxo-3,4-dihydropyrrolo[2,1-*f*][1,2,4]triazin-2-yl)ethyl)carbamate (12h)**

To a solution of triphenylphosphine (127.76 mg, 0.49 mmol) in CH_2Cl_2 (0.5 mL) was added bromine (0.03 mL, 0.49 mmol) at 0 °C. The mixture was stirred at room temperature for 15 min. Then, a solution of aminopyrrolocarbamate (**10h**, 90.3 mg, 0.24 mmol) in CH_2Cl_2 (0.5 mL) and triethylamine (0.17 mL, 1.22 mmol) were added at 0 °C. After complete addition, the reaction mixture was stirred at 0 °C for 5 min. Then, the reaction mixture was extracted with CH_2Cl_2. The organic layer was dried over anhydrous $MgSO_4$, filtered and evaporated. The resulting crude residue was purified by column chromatography on silica gel (EtOAc/*n*-hexane 1:10) to give

triazinone (**12h**, 57.2 mg, 66%) as a white solid.

mp. 140.7-141.2 °C; IR (KBr): ν 1689, 1650, 1520, 1366, 1324, 1301, 1246, 1231, 1160, 1073, 1060, 1024, 966, 744 cm^{-1};^{1}H NMR (300 MHz, $CDCl_3$) δ 6.91 (d, *J* = 3.0 Hz, 1H, pyrrolyl), 6.23 (d, *J* = 3.0 Hz, 1H, pyrrolyl), 4.94 (br, 1H, Boc-N*H*-), 4.62 (br, 1H, CH_3-C*H*(NH-Boc)-C-), 3.36-3.44 (m, 1H, cyclopropyl) 1.50 (d, *J* = 7.2 Hz, 3H, CH_3-CH(NH-Boc)-C-), 1.45 (s, 9H, *t*-butyl), 0.80-0.89 (m, 4H, cyclopropyl); ^{13}C-NMR (75 MHz, $CDCl_3$) δ 154.84 (CO), 154.75 (CO), 137.4 (C), 119.02, 119.0, 112.4, 110.7, 110.2, 110.0, 80.3, 47.4, 28.4, 28.3, 28.2, 18.9, 8.8; HRMS (EI) calcd for $C_{16}H_{21}ClN_4O_3$ 352.1302, found 352.1300.

Introduction of mosher's acid for determining enantiomeric excess (ee)

To a solution of pyrrolotriazinone (**12a**, 27 mg, 0.07 mmol) in CH_2Cl_2 (0.5 mL) was added trifluoroacetic acid (0.3 mL, 4.1 mmol) at 0 °C. The mixture was stirred at room temperature for 4 h. Then, the reaction mixture was quenched with aq. $NaHCO_3$ (3 mL) and extracted with CH_2Cl_2. The organic layer was dried over anhydrous Na_2SO_4, filtered and evaporated. The resulting crude residue was then dried under high vaccum and used to the next reaction without further purification.

To a solution of free amine containing **12a** (20 mg, 0.07 mmol) in THF (0.5 mL) was added 1-[bis(dimethylamino)methylene]-1*H*-1,2,3-triazolo[4,5-*b*]pyridinium 3-oxide hexafluorophosphate (HATU, 29 mg, 0.08 mmol), DIPEA (0.02 mL, 0.1 mmol), and (*S*)-(−)-α-methoxy-α-(trifluoromethyl)phenylacetic acid (Mosher's acid, 18 mg, 0.08 mmol) at 0 °C. The mixture was stirred at room temperature for 4 h, a solvent was

concentrated under reduced pressure. The mixture was diluted with EtOAc and H_2O, and the layers were separated. The organic layer was dried over anhydrous $MgSO_4$, filtered and evaporated. The resulting crude residue was purified by column chromatography on silica gel (EtOAc/*n*-hexane 1:5) to give ((*S*)-*N*-((*S*)-1-(5-chloro-4-oxo-3-phenyl-3,4-dihydropyrrolo[2,1-*f*][1,2,4]triazin-2-yl)ethyl)-3,3,3-trifluoro-2-methoxy-2-phenylpropanamide (25 mg, 71%) as a white solid.

Rearrangement reactions

1. Entry 2, Table 2

   To a solution of thiophenol (0.02 mL, 0.23 mmol) in dry THF (0.5 mL) was dropwise added *n*-BuLi (0.09 mL, 0.23 mmol, 2.5 M in hexane) under a nitrogen atmosphere at −78 °C. After stirring for 10 min, $AlMe_3$ (0.21 mL, 0.23 mmol, 2.0 M in toluene) was dropwise added. The reaction mixture was stirred at -78 ^{o}C for 10 min. After adding a solution of *tert*-butyl (*S*,*Z*)-(1-(5-chloro-4-(phenylimino)-4*H*-pyrrolo[1,2-*d*][1,3,4]oxadiazin-2-yl)ethyl)carbamate (**11a**, 30.0 mg, 0.077 mmol) in dried THF (0.8 mL), the reaction mixture was stirred at room temperature for 20 h. Then, the reaction mixture was quenched with 1 N HCl (0.5 mL), and extracted with EtOAc. The organic layer was dried over anhydrous $MgSO_4$, filtered and evaporated. The resulting crude residue was purified by column chromatography on silica gel (EtOAc/*n*-Hexane, 1:5) to give *tert*-butyl (*S*)-(1-(5-chloro-4-oxo-3-phenyl-3,4-dihydropyrrolo[2,1-*f*][1,2,4]triazin-2-yl)ethyl)carbamate (**12a**, 26.8 mg, 90%) as a white solid.

2. Entry 3, Table 2

To a solution of thiophenol (0.02 mL, 0.23 mmol) in dry THF (0.5 mL) was dropwise added *n*-BuLi (0.09 mL, 0.23 mmol, 2.5 M in hexane) under a nitrogen atmosphere at −78 °C. After stirring for 10 min, $AlMe_3$ (0.21 mL, 0.23 mmol, 2.0 M in toluene,) was dropwise added. The reaction mixture was stirred at −78 °C for 10 min. After adding a solution of *tert*-butyl (*S*,*Z*)-(1-(5-chloro-4-((4-fluorophenyl)imino)-4*H*-pyrrolo[1,2-*d*][1,3,4]oxadiazin-2-yl)ethyl)carbamate (**11d**, 31.4 mg, 0.077 mmol) in dry THF (0.8 mL), the reaction mixture was stirred at room temperature for 20 h. Then, the reaction mixture was quenched with 1 N HCl (0.5 mL), and extracted with EtOAc. The organic layer was dried over anhydrous $MgSO_4$, filtered and evaporated. The resulting crude residue was purified by column chromatography on silica gel (EtOAc/*n*-hexane, 1:7) to give *tert*-butyl (*S*)-(1-(5-chloro-3-(4-fluorophenyl)-4-oxo-3,4-dihydropyrrolo[2,1-*f*][1,2,4]triazin-2-yl)ethyl)carbamate (**12d**, 21.7 mg, 69%) as a white solid.

3. Entry 4, Table 2

To a solution of *tert*-butyl (*S*,*Z*)-(1-(5-chloro-4-(phenylimino)-4*H*-pyrrolo[1,2-*d*][1,3,4]oxadiazin-2-yl)ethyl)carbamate (**11a**, 10.0 mg, 0.03 mmol) in dry THF/DMF (9:1) (0.3 mL) was added sodium thiomethoxide (95%) (5.69 mg, 0.08 mmol) at room temperature. After stirring at room temperature for 0.5 h, the reaction mixture was quenched with aq. $NaHCO_3$ (0.5 mL) at 0 °C. Then, the reaction mixture was extracted with EtOAc. The organic layer was dried over anhydrous $MgSO_4$, filtered and evaporated. The resulting solid was

washed with *n*-hexane / diethyl ether to give *tert*-butyl (*S*)-(1-(5-chloro-4-oxo-3-phenyl-3,4-dihydropyrrolo[2,1-*f*][1,2,4]triazin-2-yl)ethyl)carbamate (**12a**, 9.2 mg, 92%) as a white solid.

4. Entry 5, Table 2

To a solution of *tert*-butyl (*S*,*Z*)-(1-(5-chloro-4-(phenylimino)-4*H*-pyrrolo[1,2-*d*][1,3,4]oxadiazin-2-yl)ethyl)carbamate (**11a,** 10.0 mg, 0.03 mmol) in dried THF/DMF (9:1) (0.5 mL) was added sodium methoxide (95%) (4.39 mg, 0.08 mmol) at room temperature. After stirring at room temperature for 3 h, the reaction mixture was quenched with aq. $NaHCO_3$ (0.5 mL) at 0 °C. Then, the reaction mixture was extracted with EtOAc. The organic layer was dried over anhydrous $MgSO_4$, filtered and evaporated. The resulting crude residue was purified by column chromatography on silica gel (EtOAc/*n*-Hexane, 1:2) to give *tert*-butyl (*S*)-(1-(5-chloro-4-oxo-3-phenyl-3,4-dihydropyrrolo[2,1-*f*][1,2,4]triazin-2-yl)ethyl)carbamate (**12a**, 8.5 mg, 85%) as a white solid.

^{1}H and ^{13}C NMR of compound **14a**

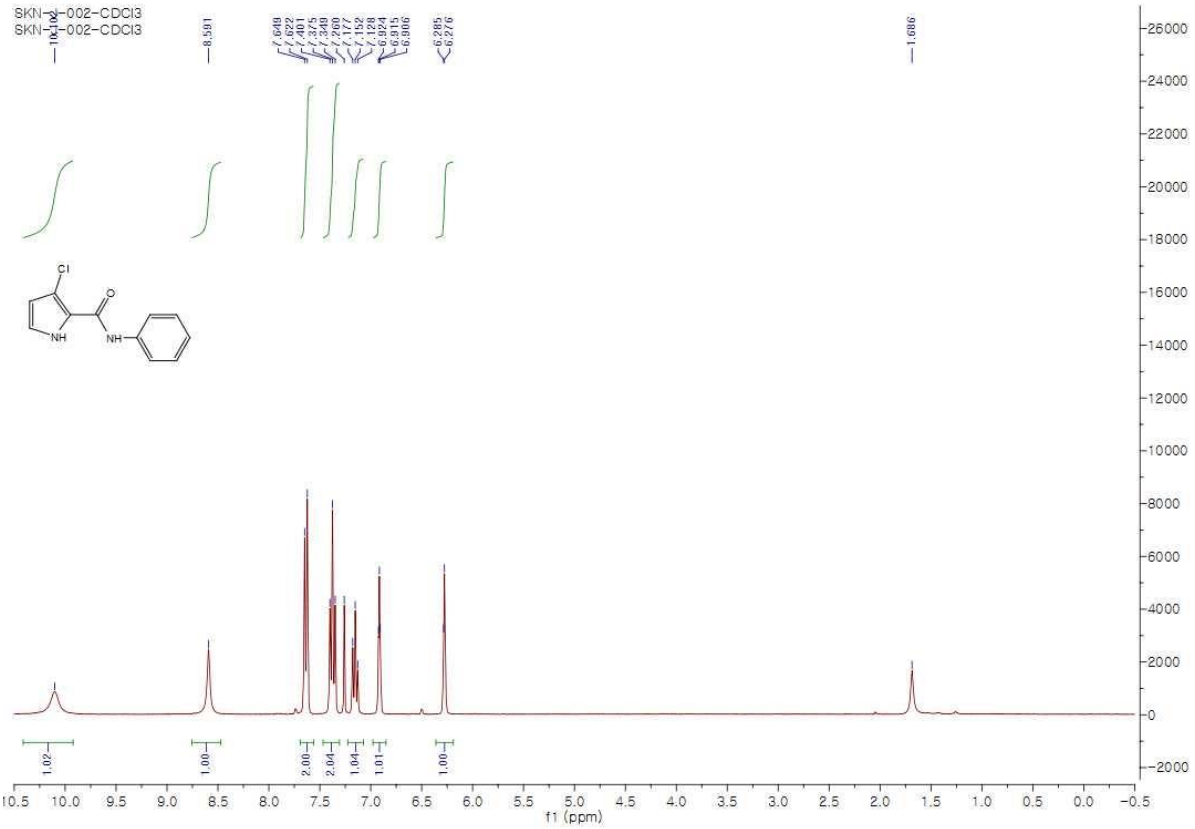

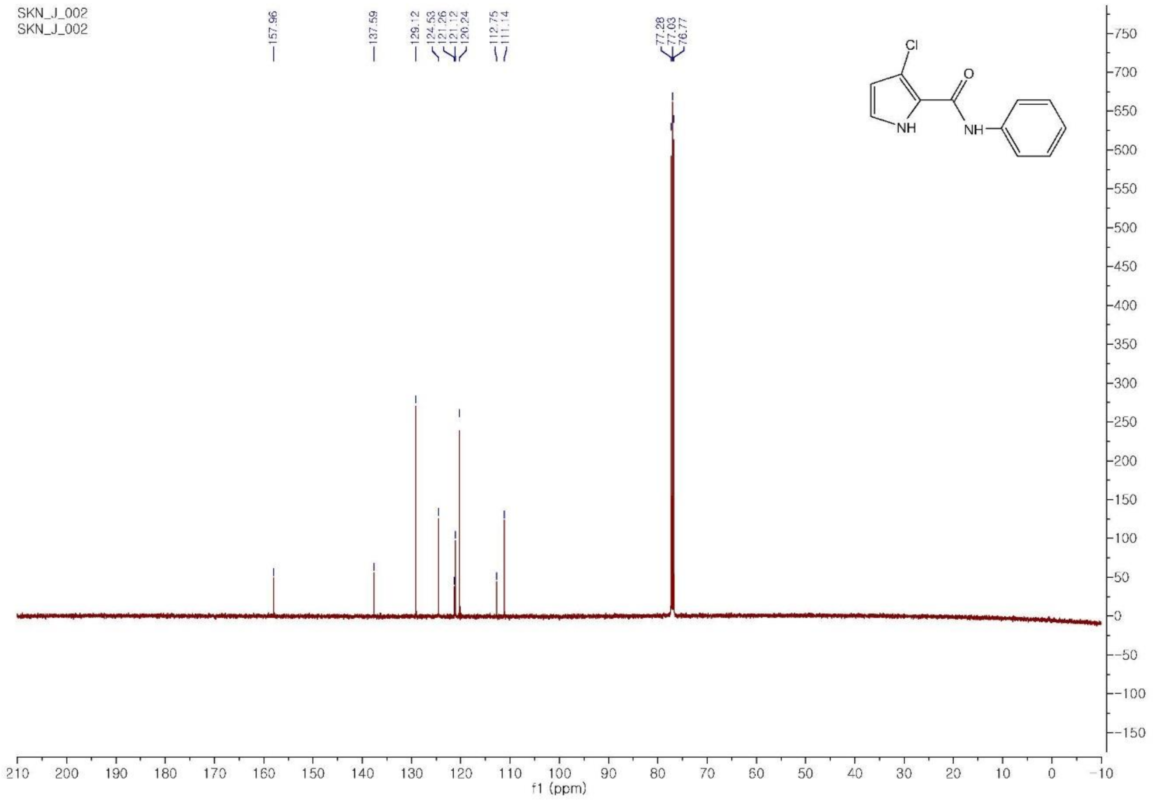

^{1}H and ^{13}C NMR of compound **15a**

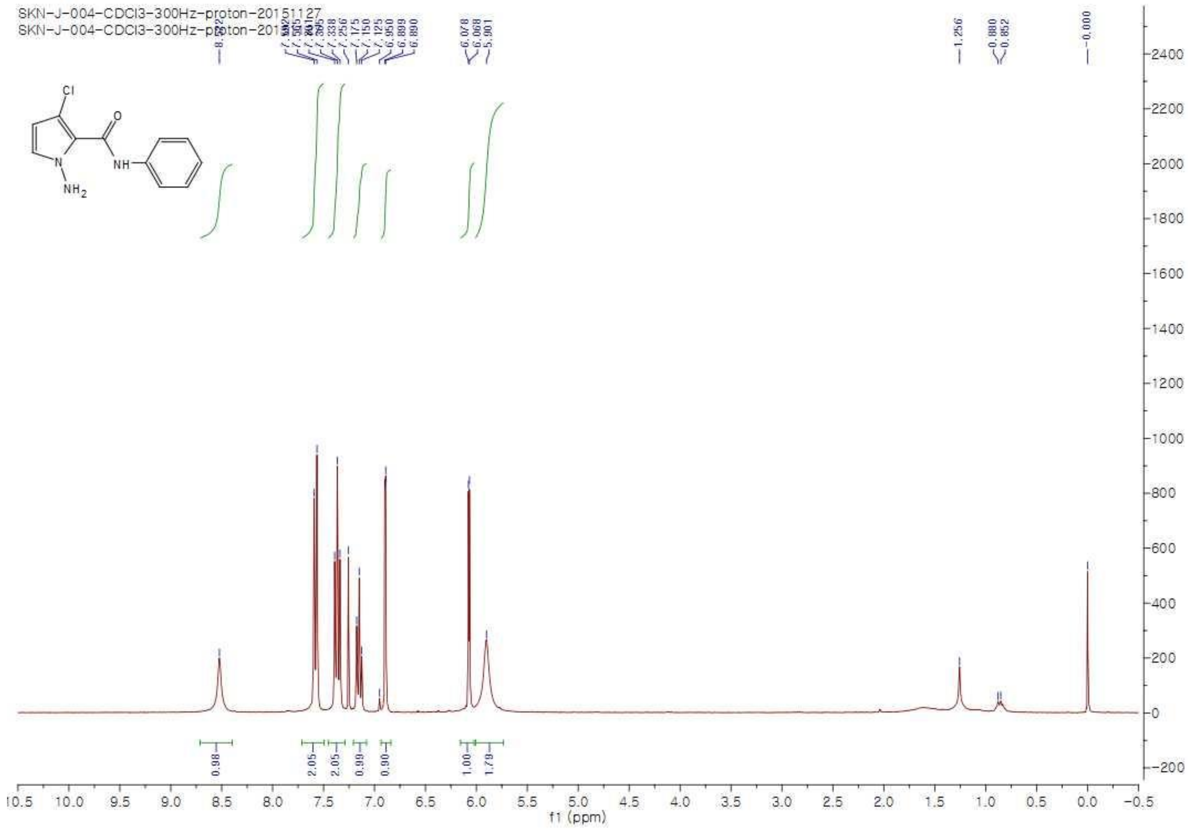

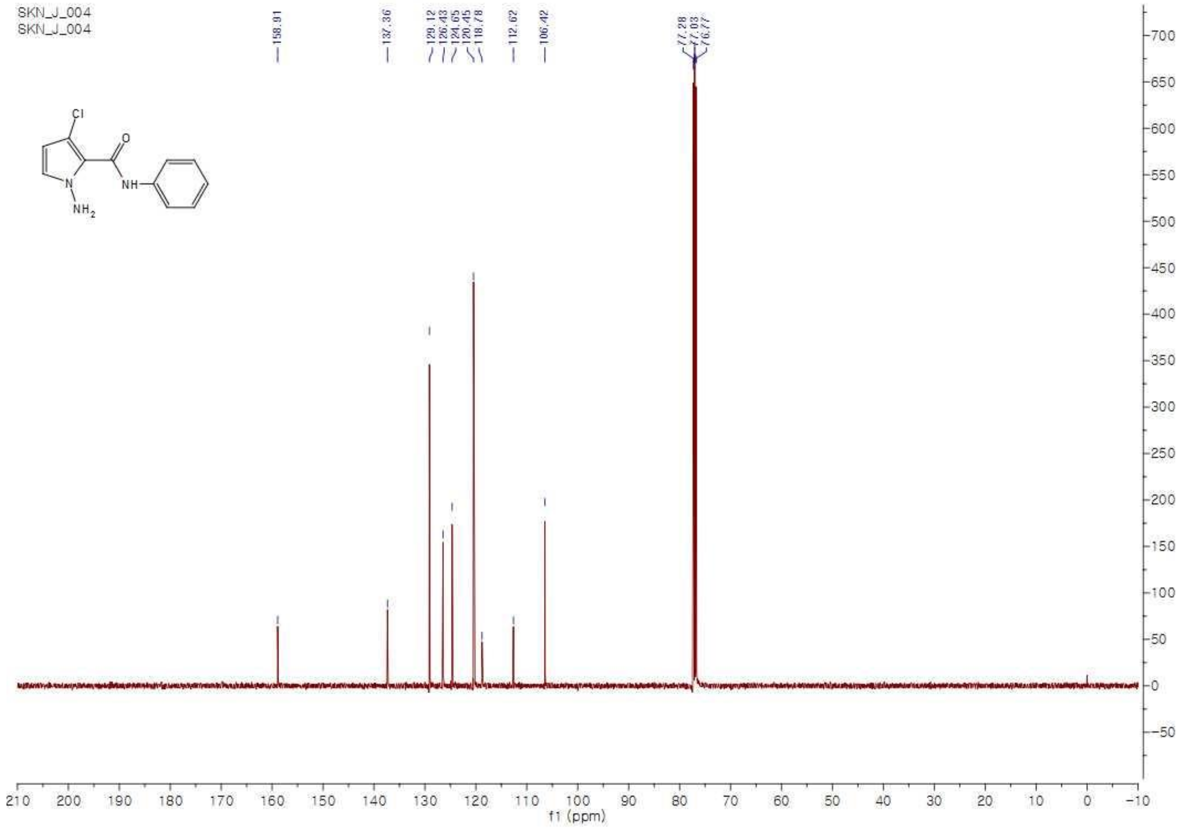

^{1}H and ^{13}C NMR of compound **10a**

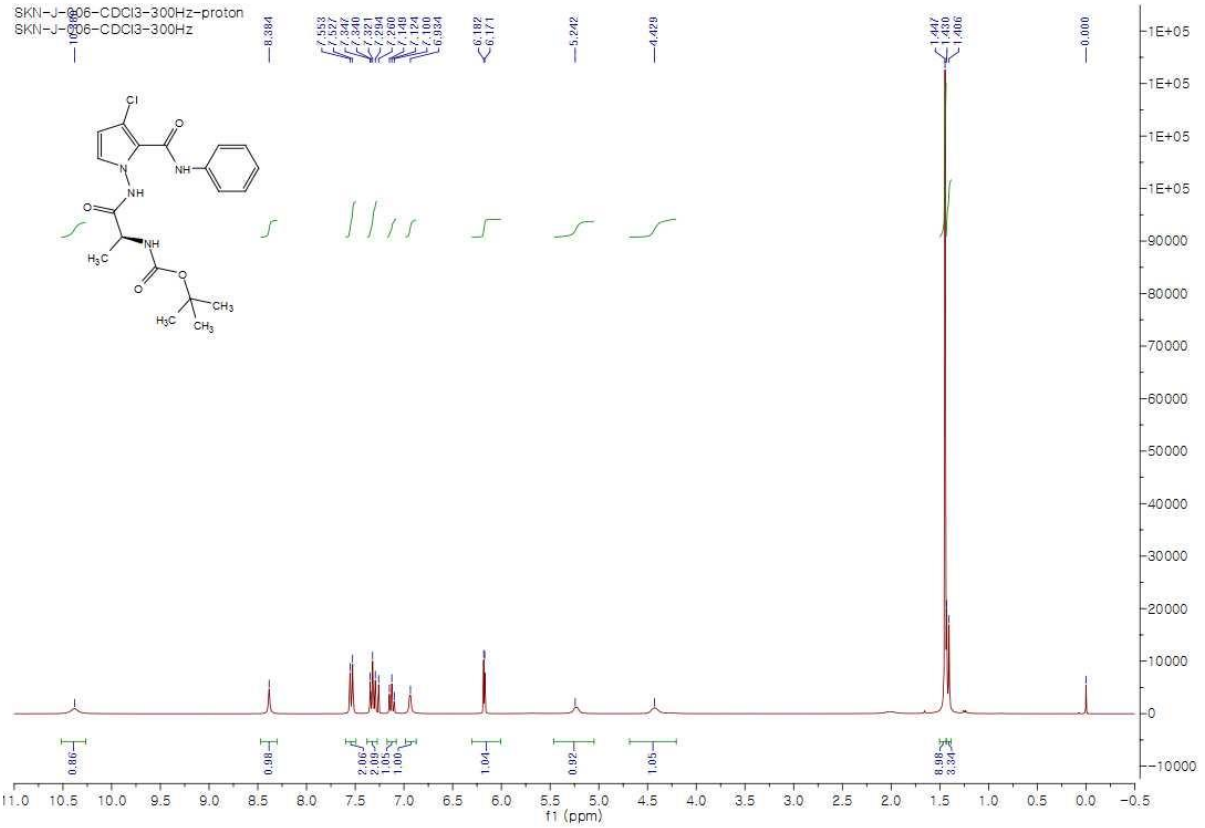

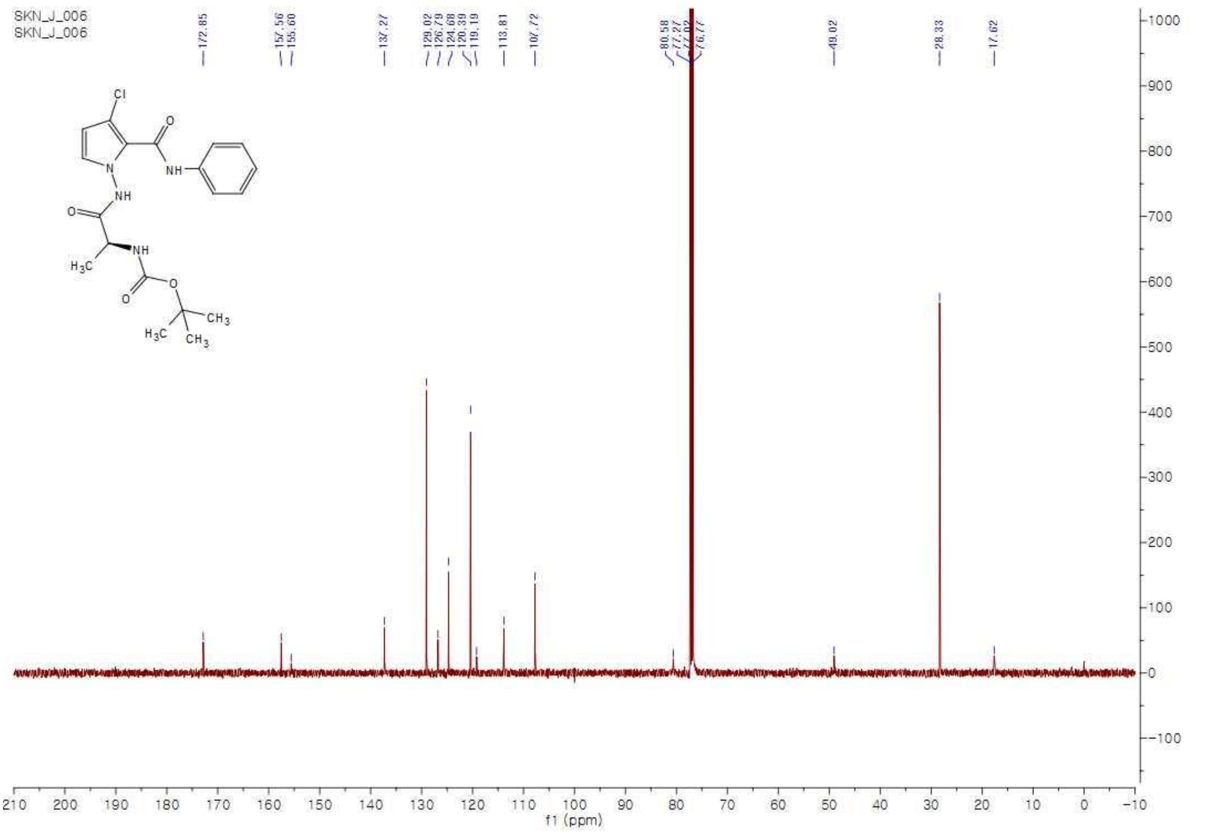

^{1}H and ^{13}C NMR of compound **11a**

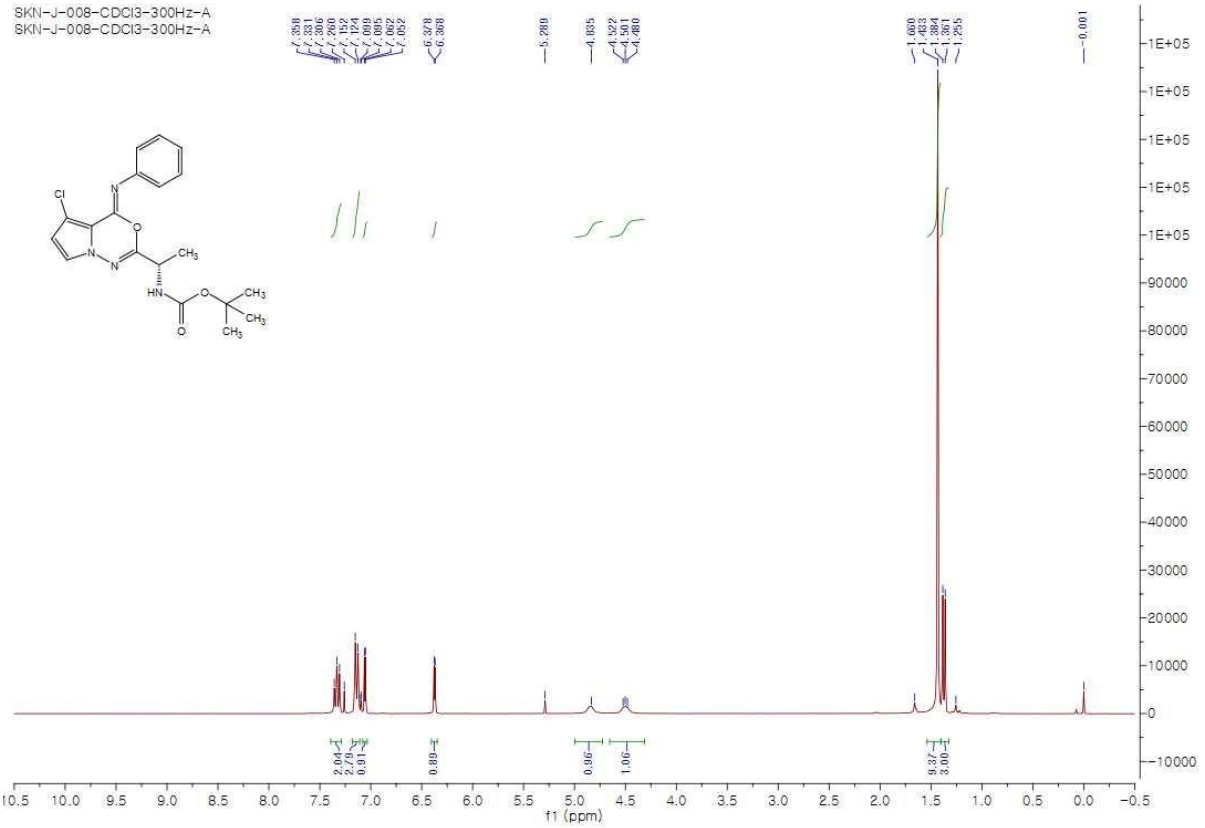

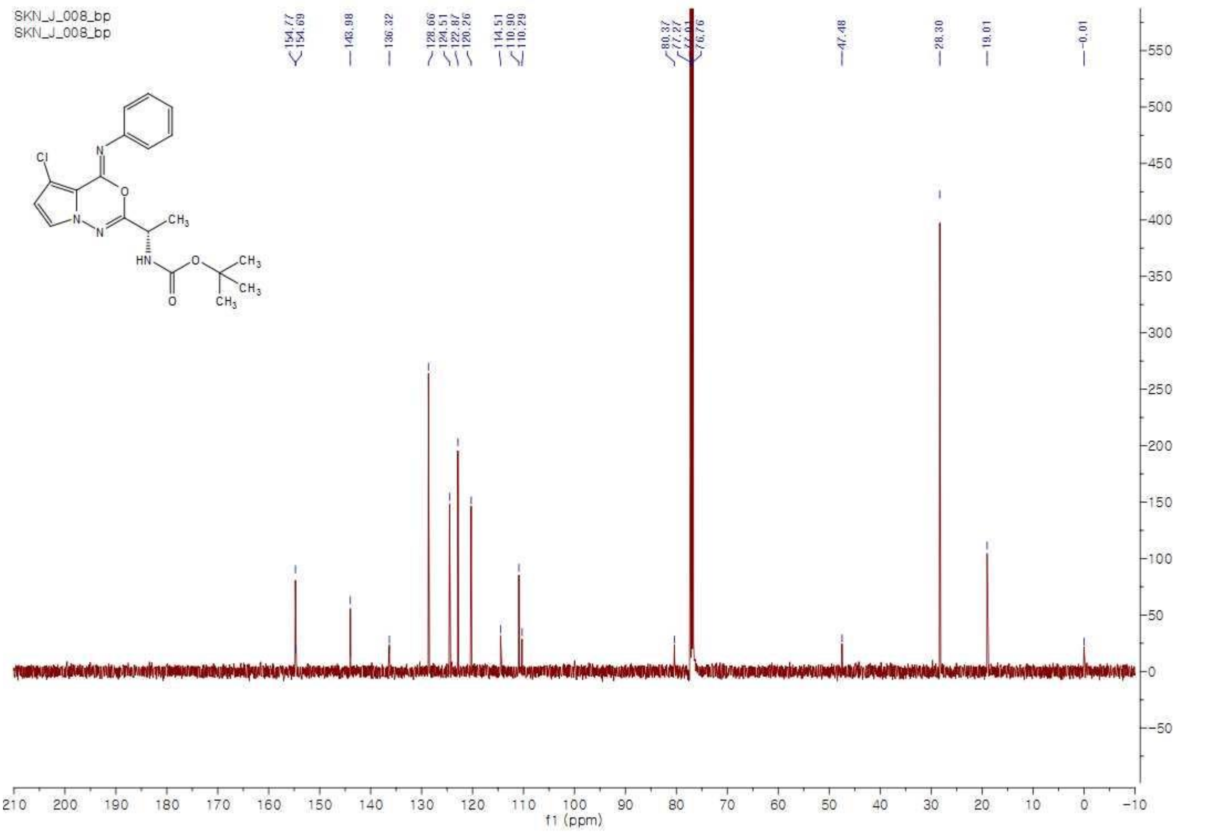

^{1}H and ^{13}C NMR of compound **12a**

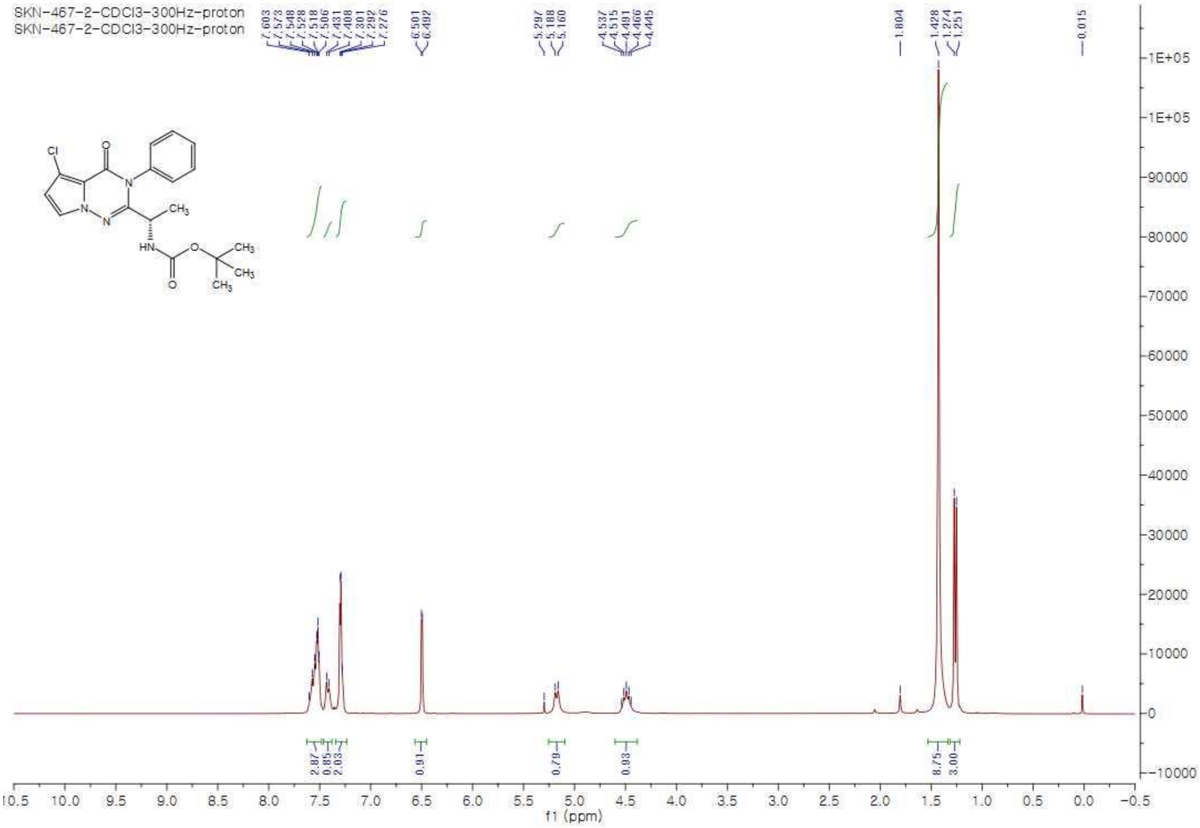

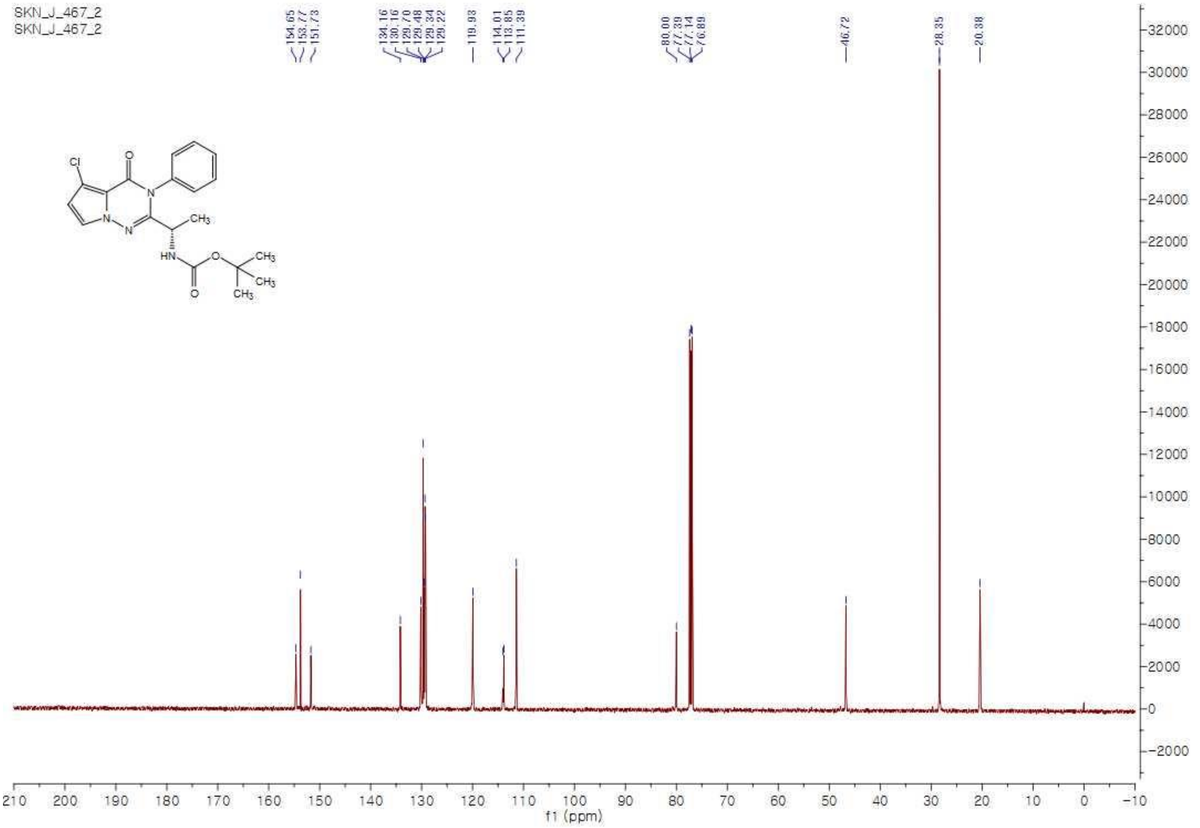

^{1}H and ^{13}C NMR of compound **14b**

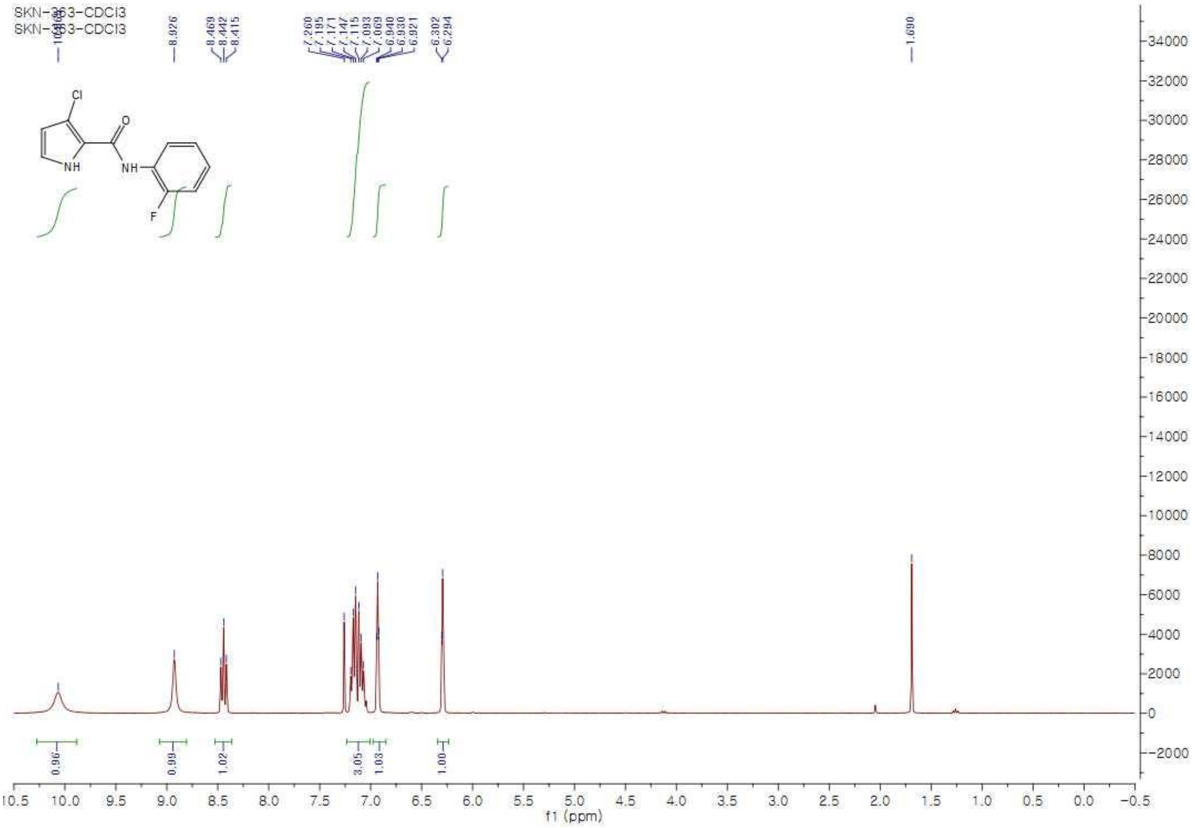

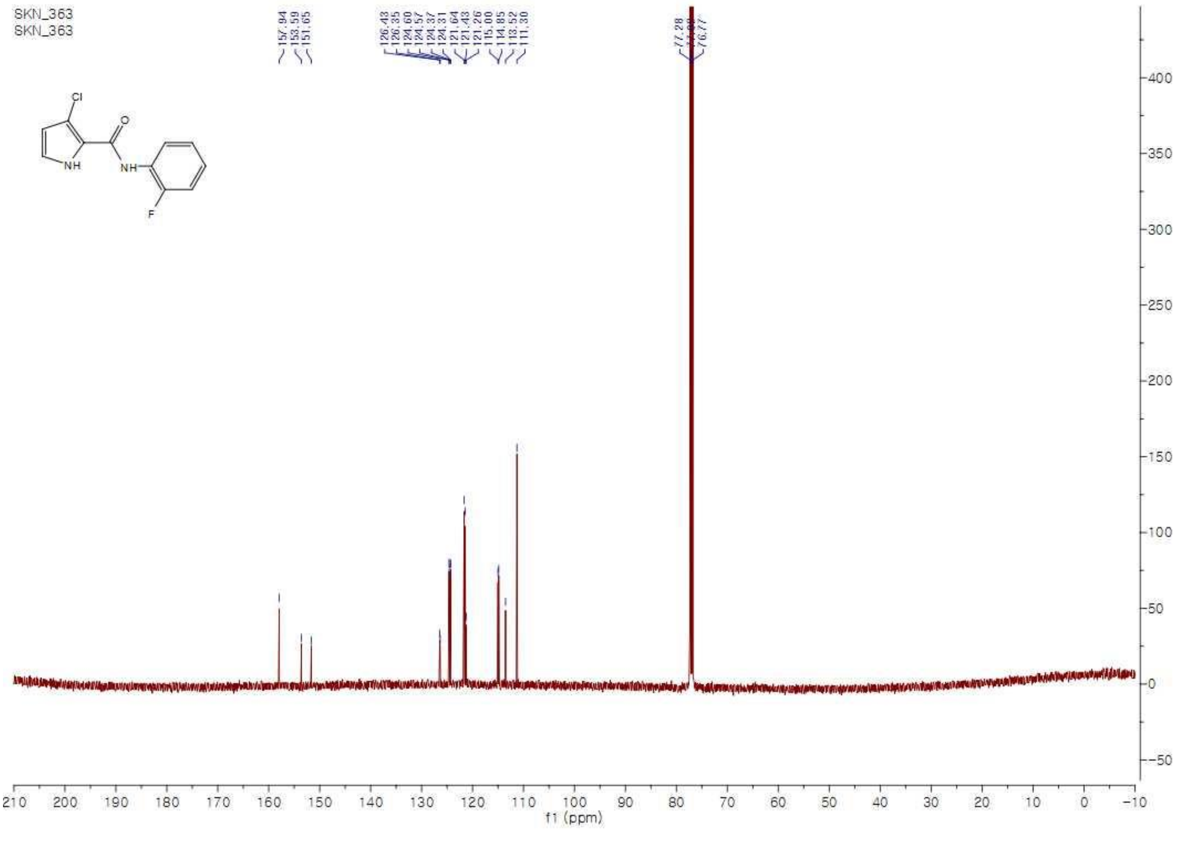

^{1}H and ^{13}C NMR of compound **15b**

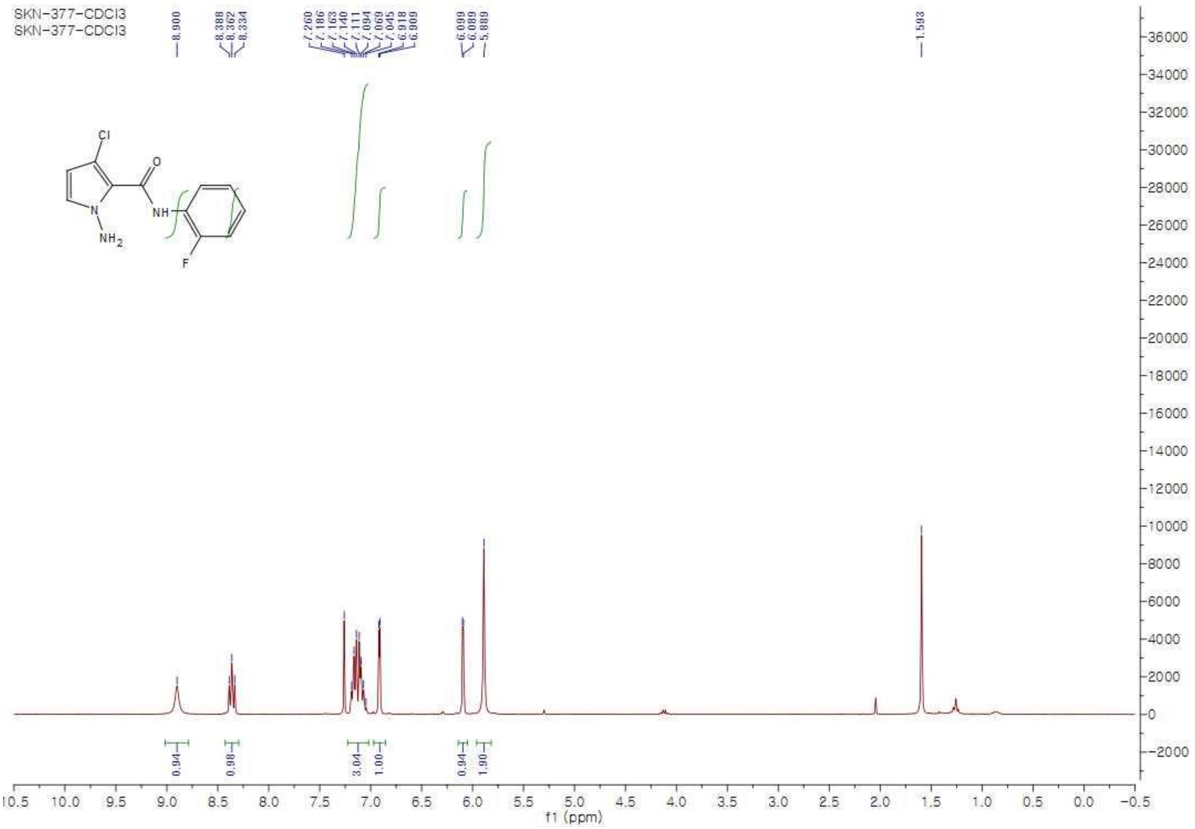

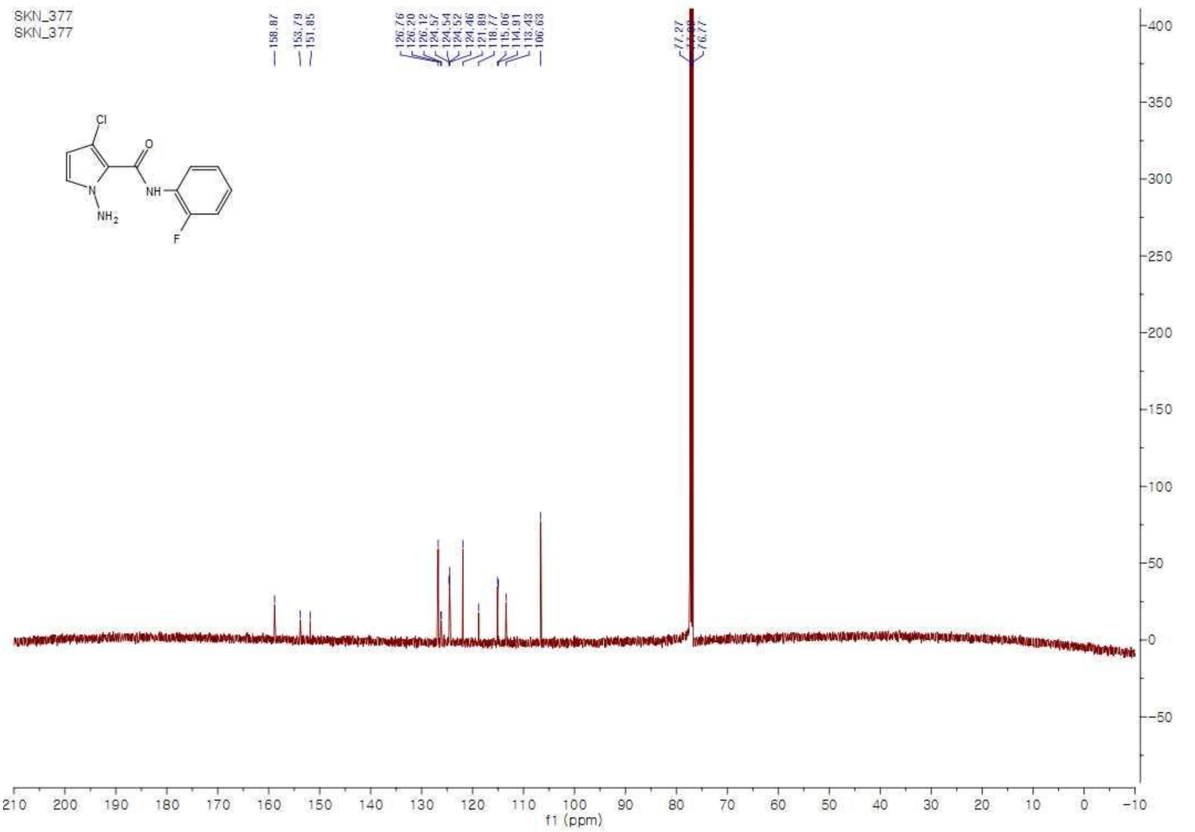

^{1}H and ^{13}C NMR of compound **10b**

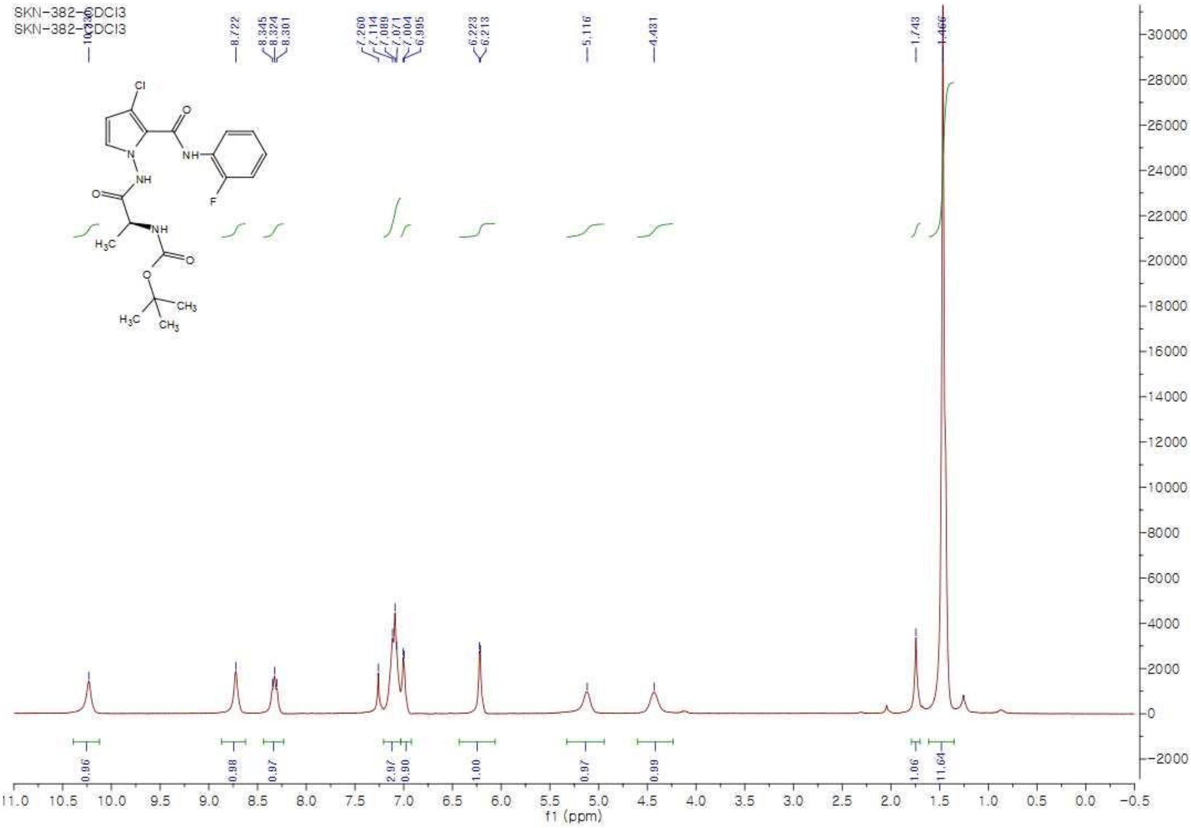

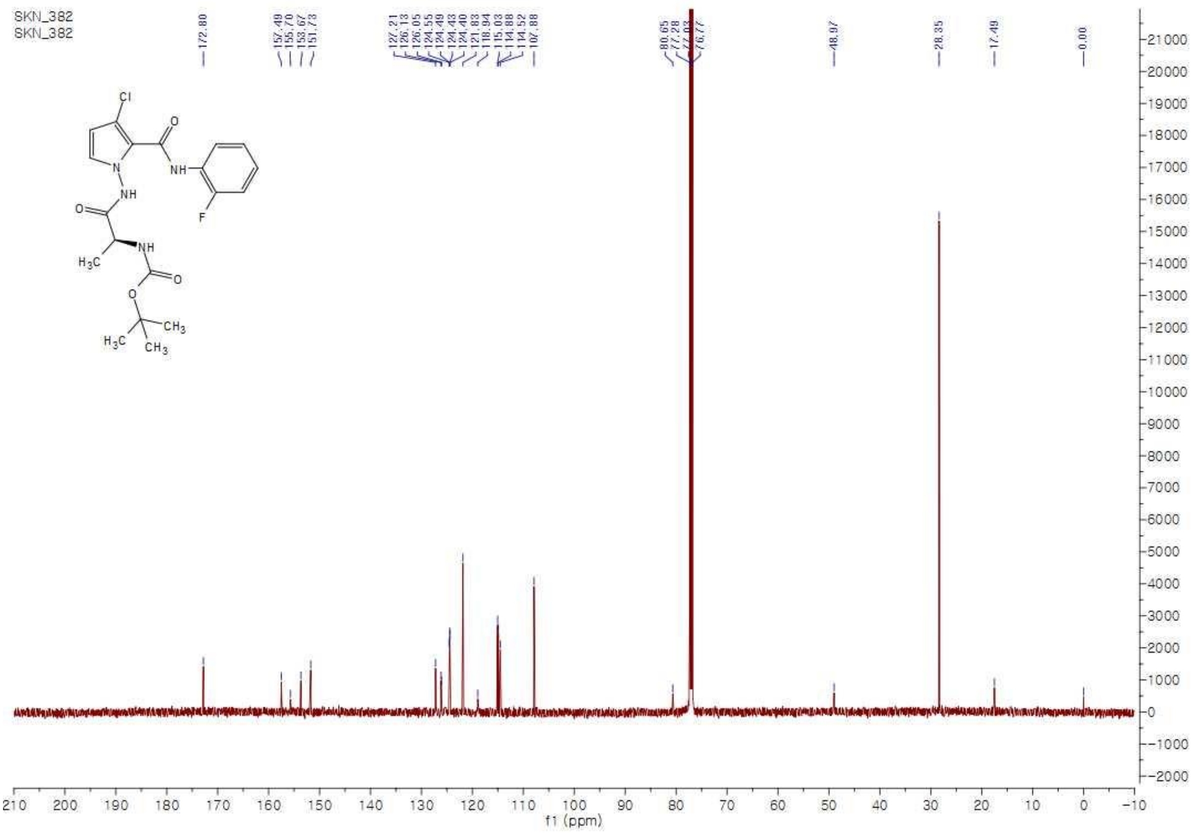

^{1}H and ^{13}C NMR of compound **11b**

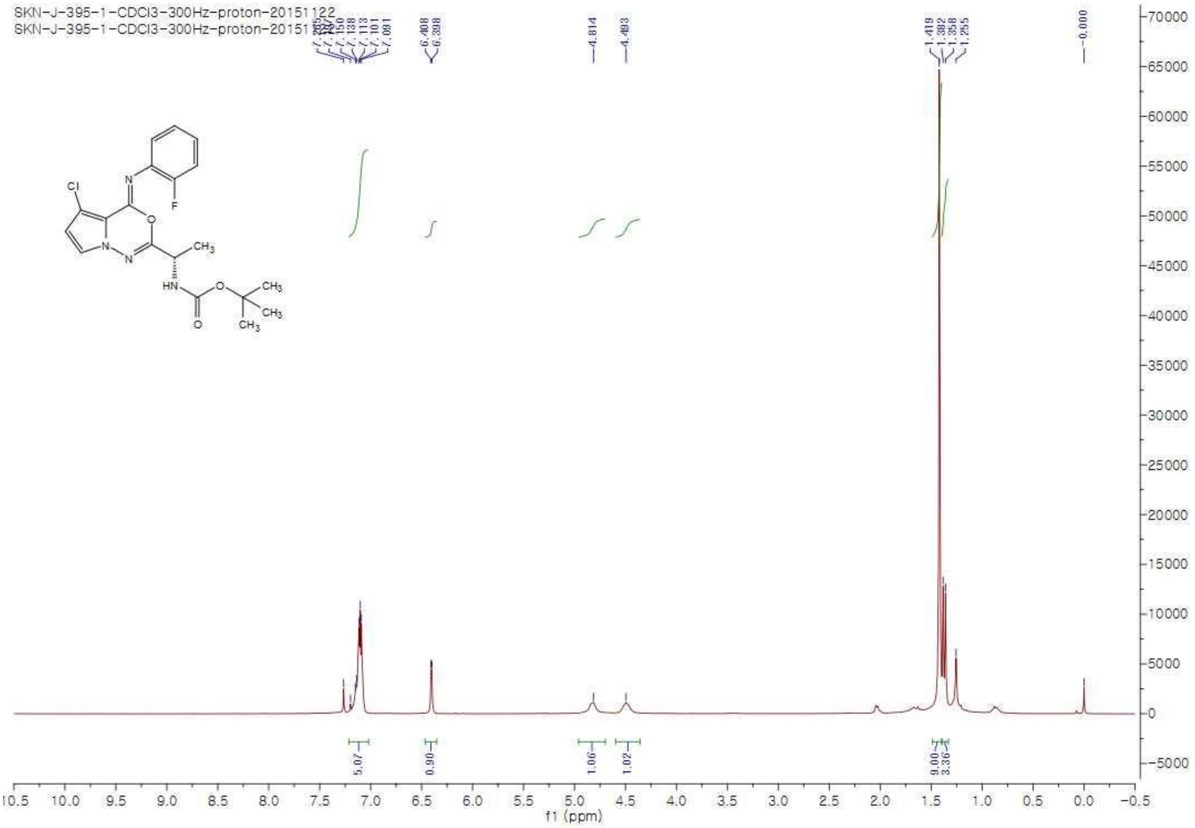

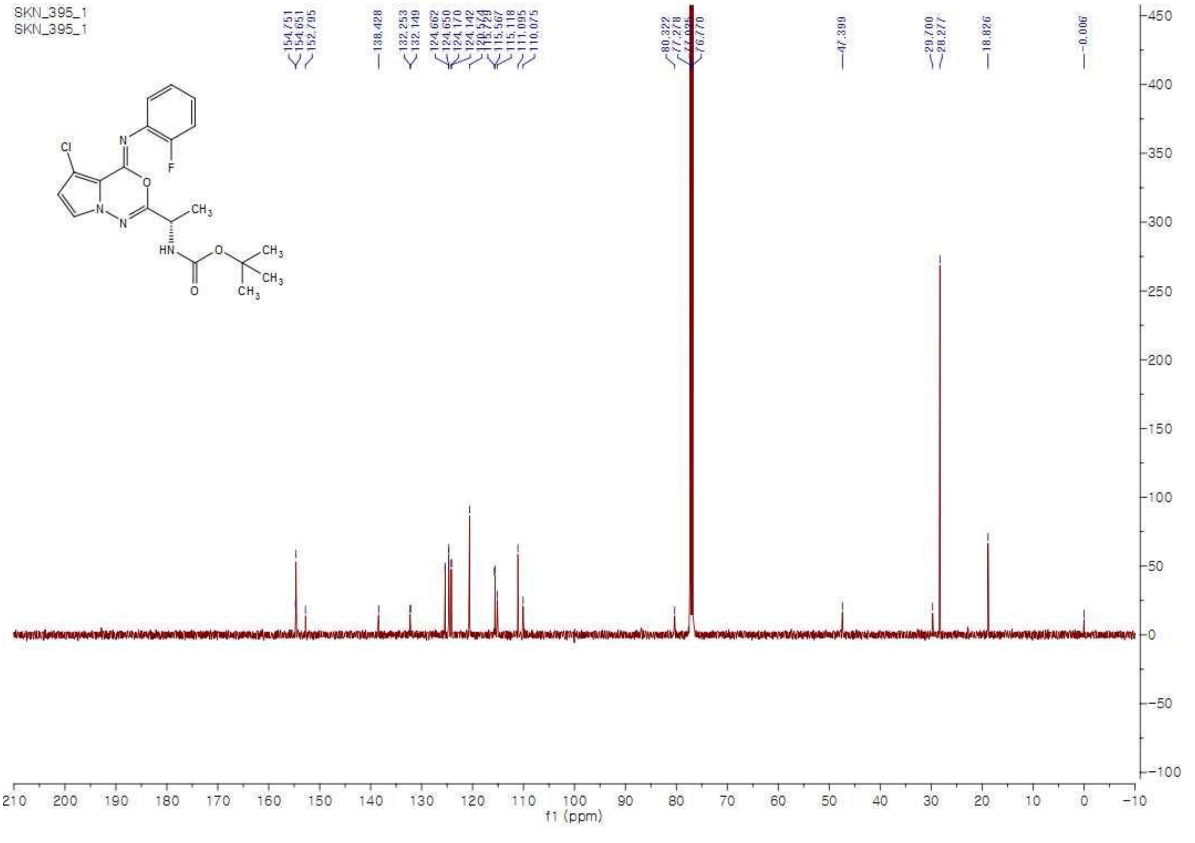

^{1}H and ^{13}C NMR of compound **12b**

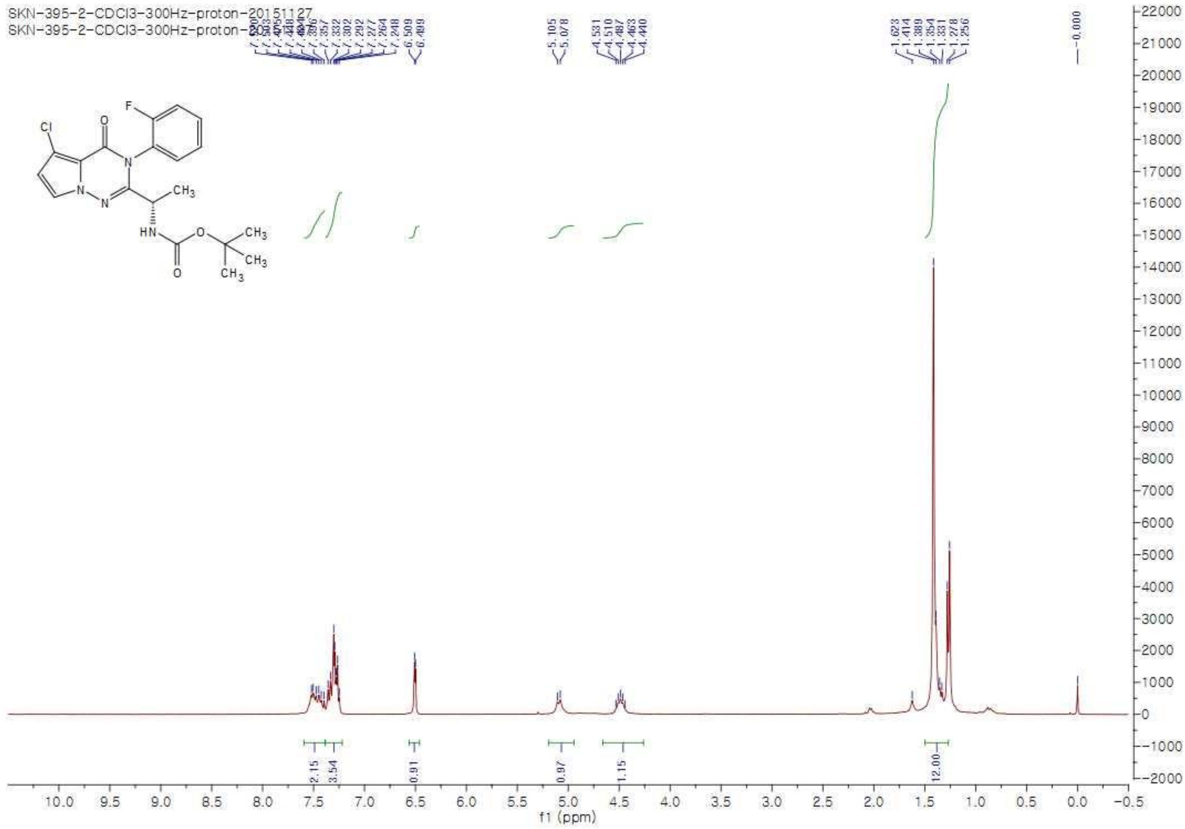

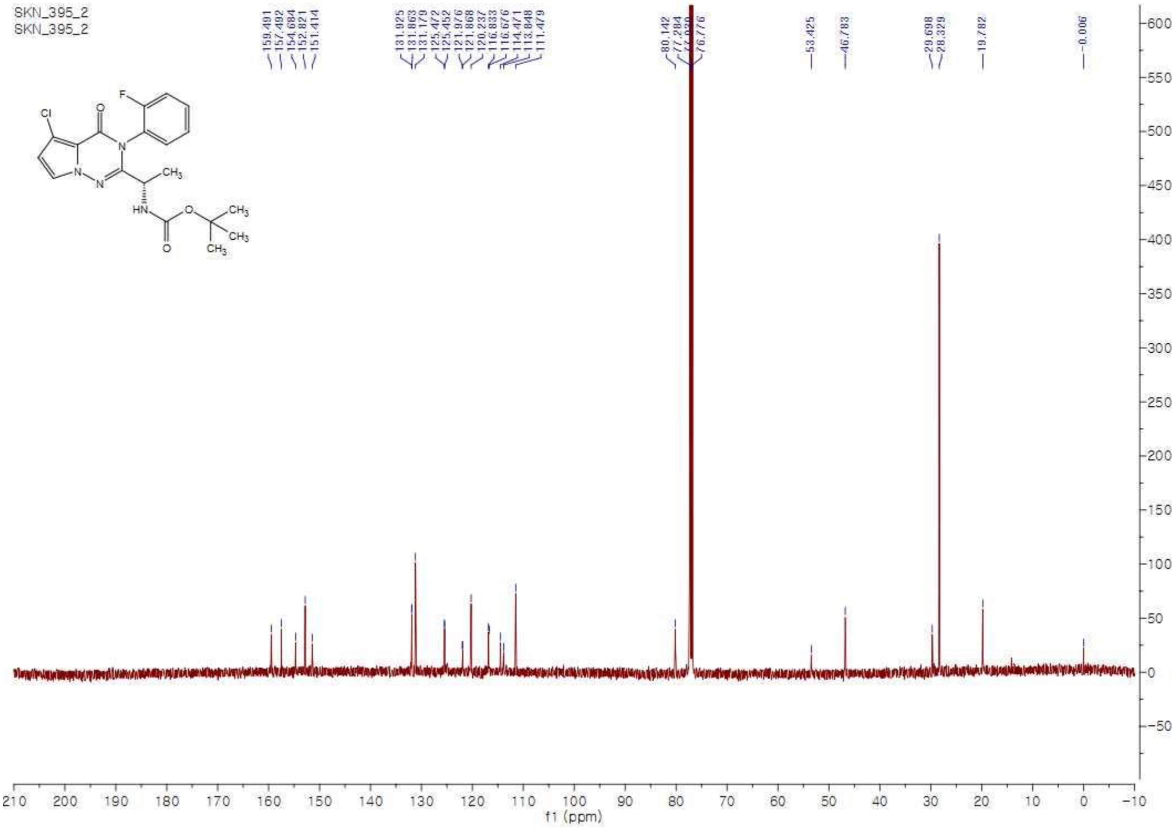

^{19}F NMR of compound **11b** and **12b**

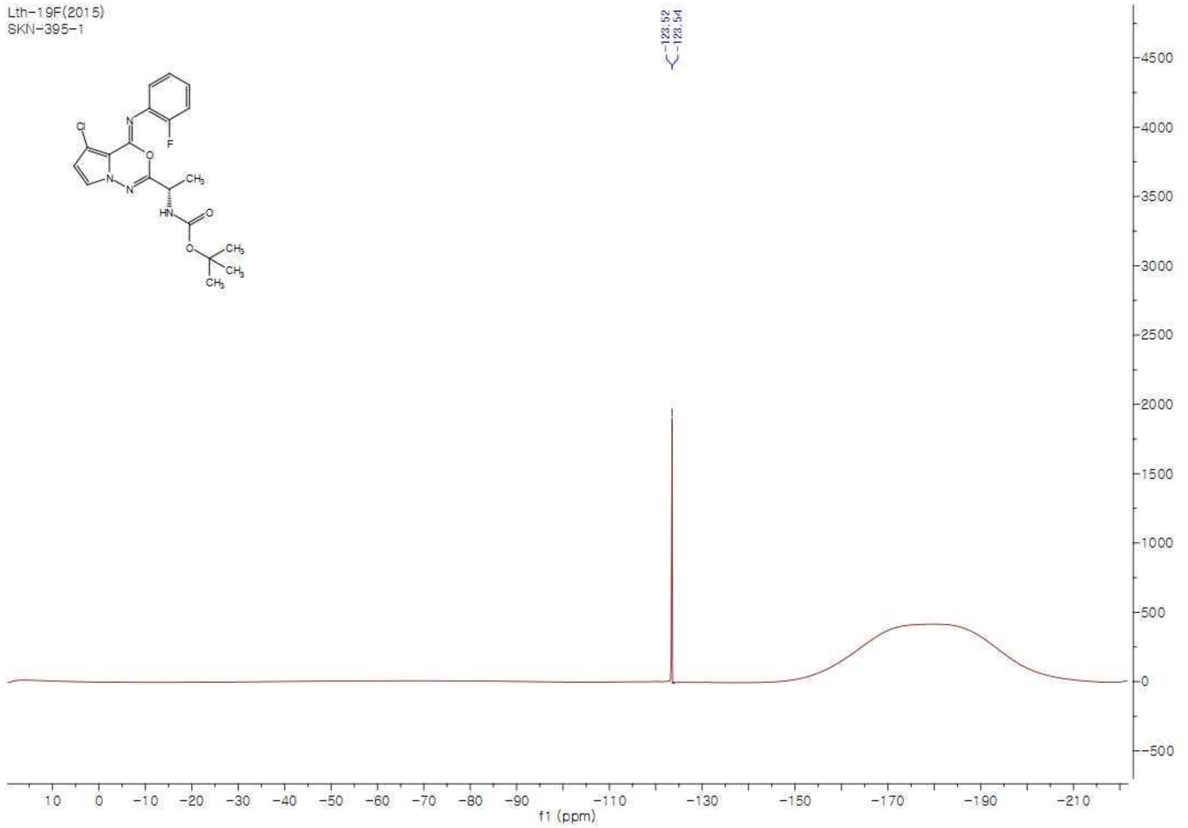

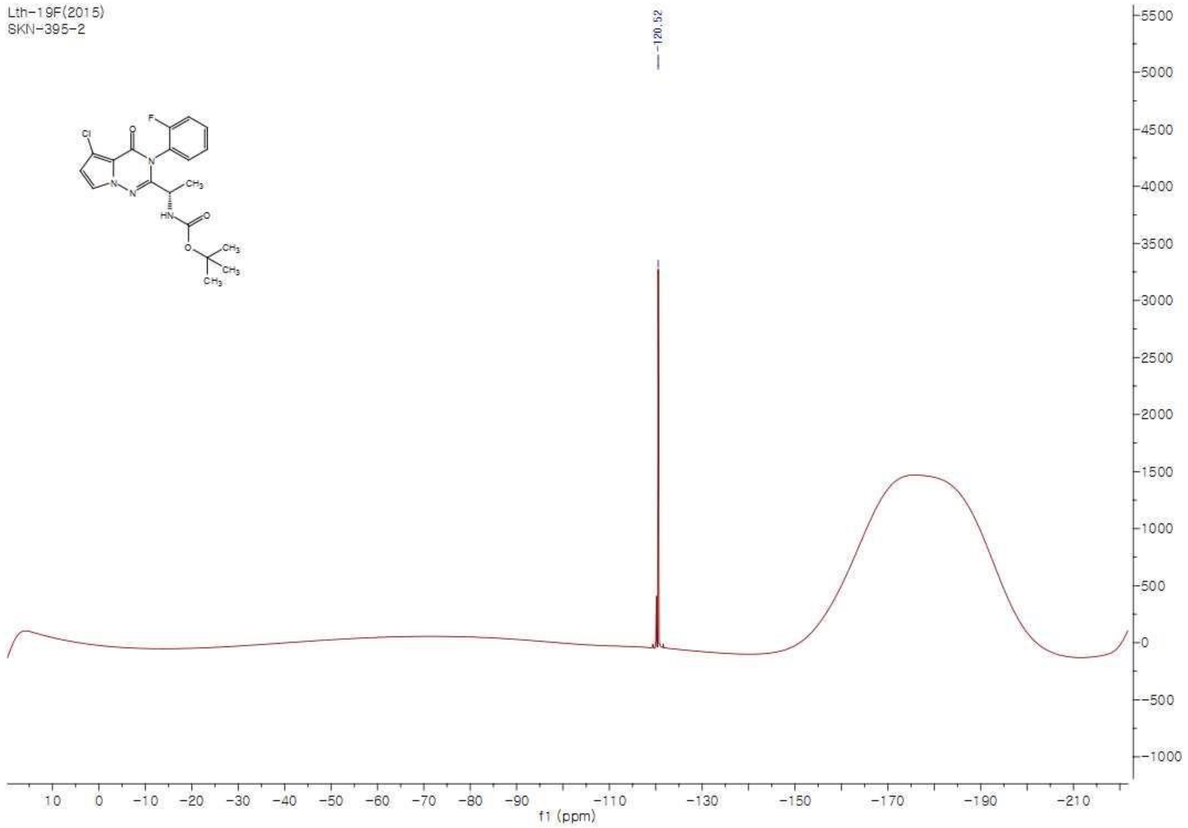

^{1}H and ^{13}C NMR of compound **14c**

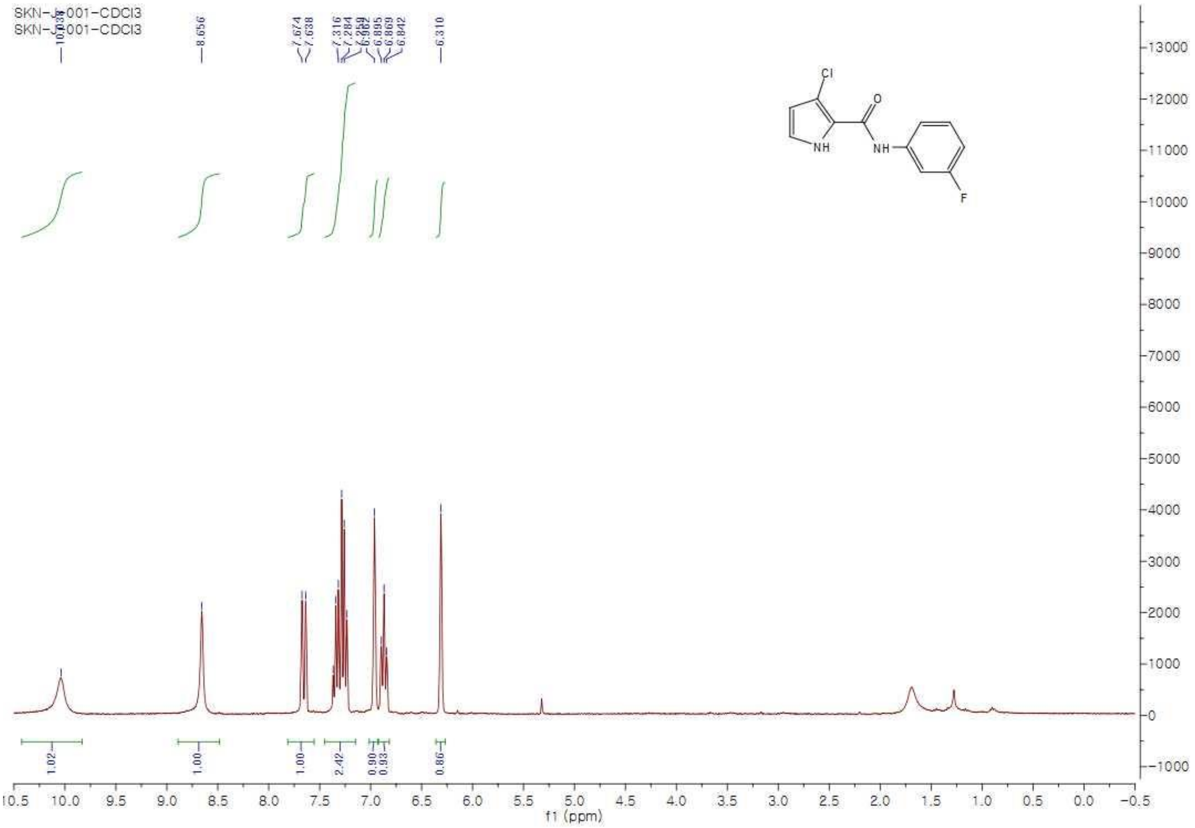

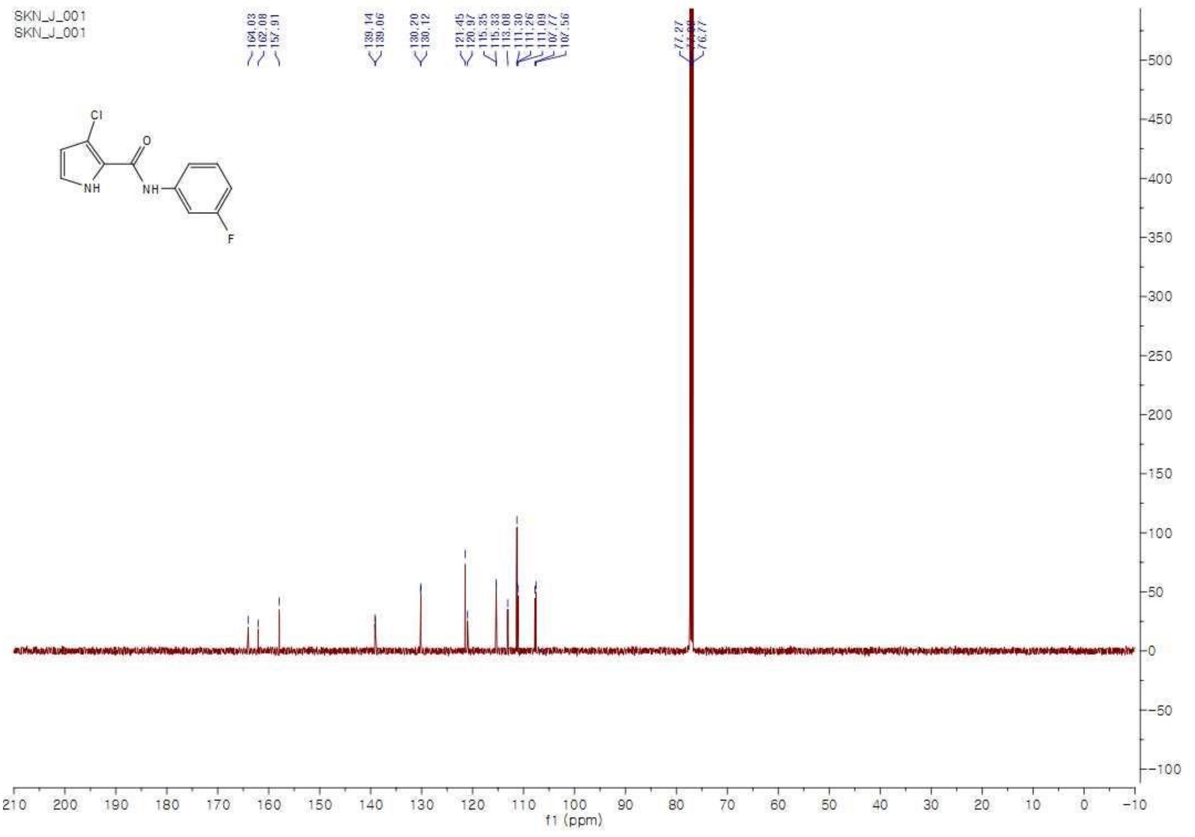

^{1}H and ^{13}C NMR of compound **15c**

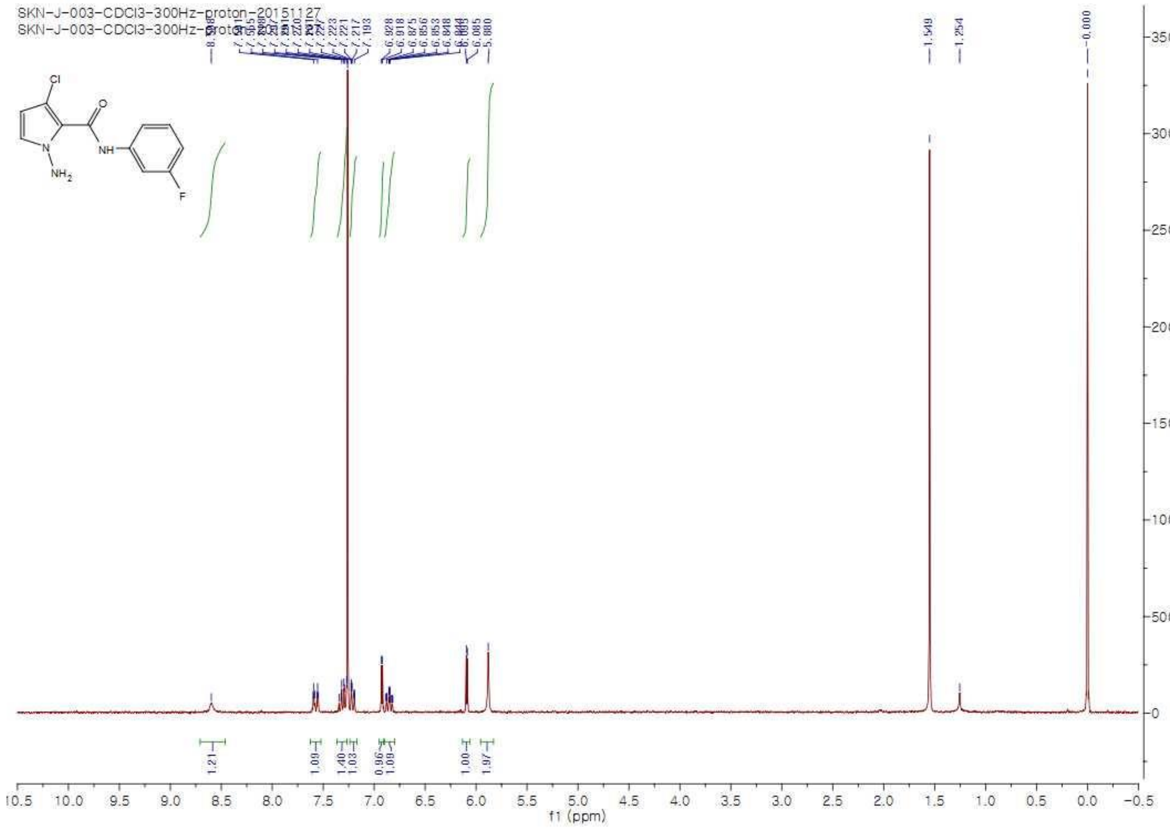

^{1}H and ^{13}C NMR of compound **10c**

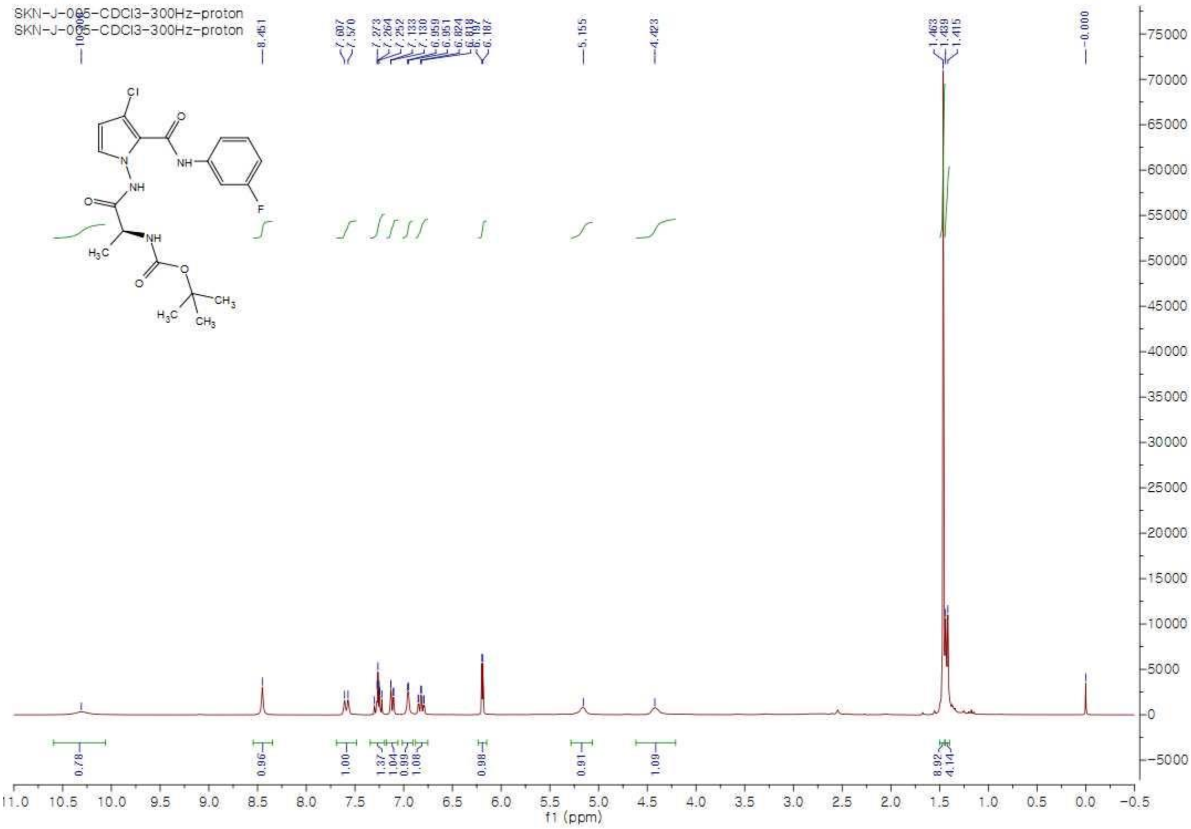

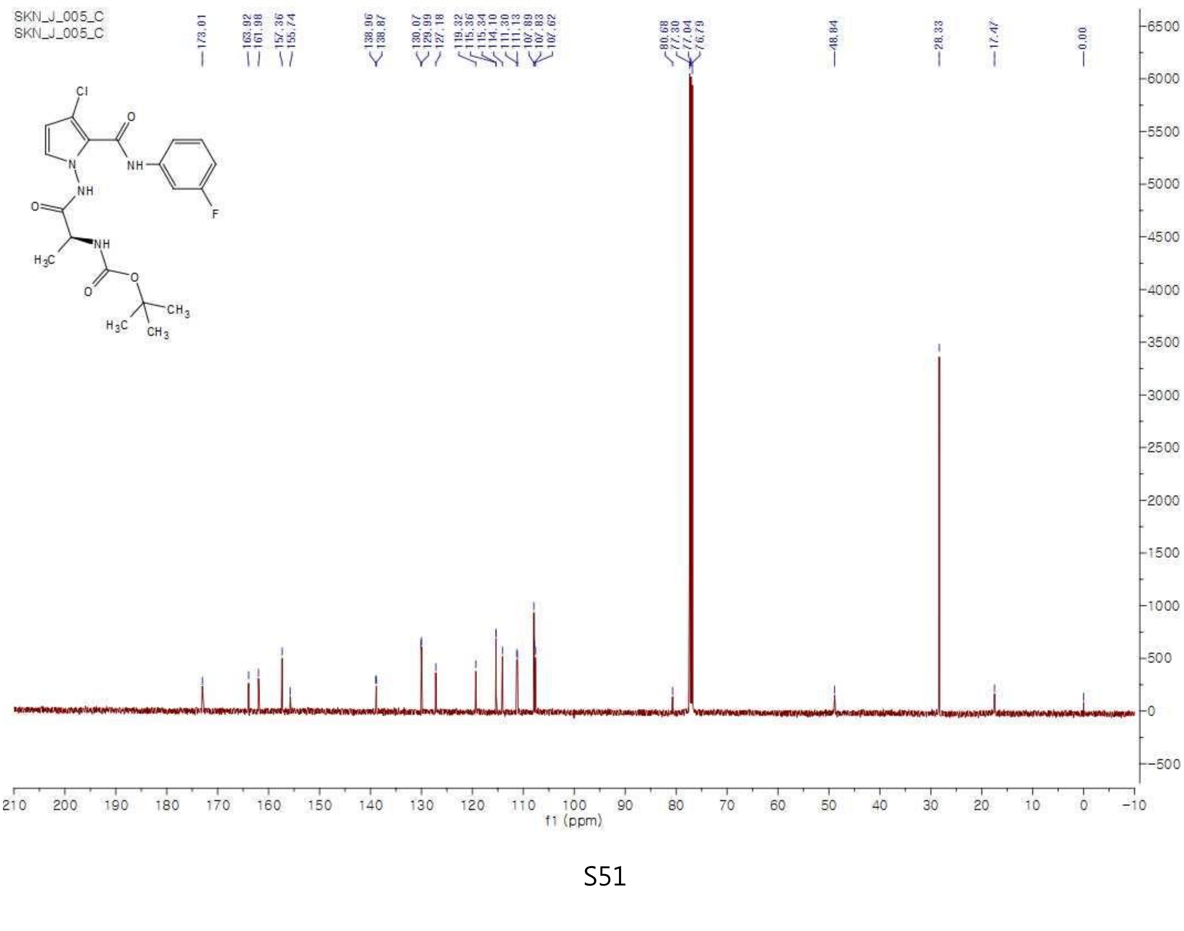

^{1}H and ^{13}C NMR of compound **11c**

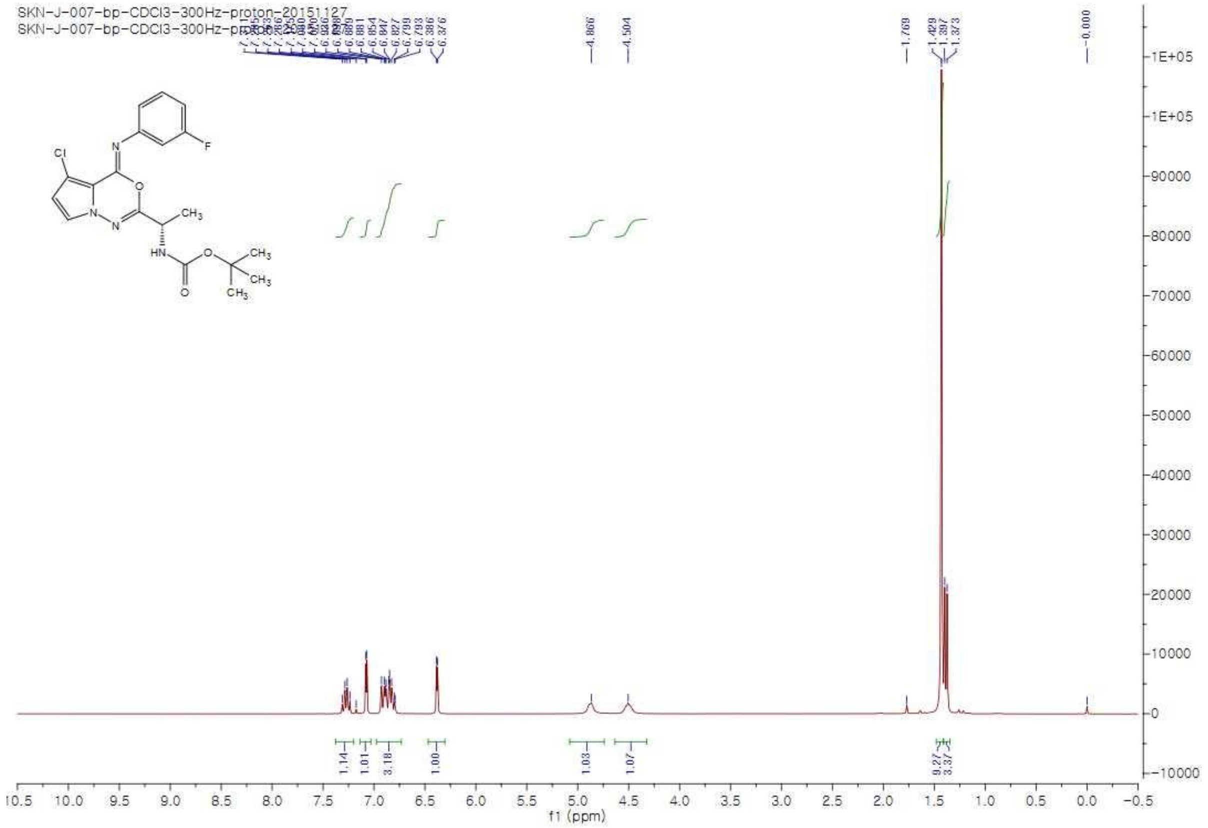

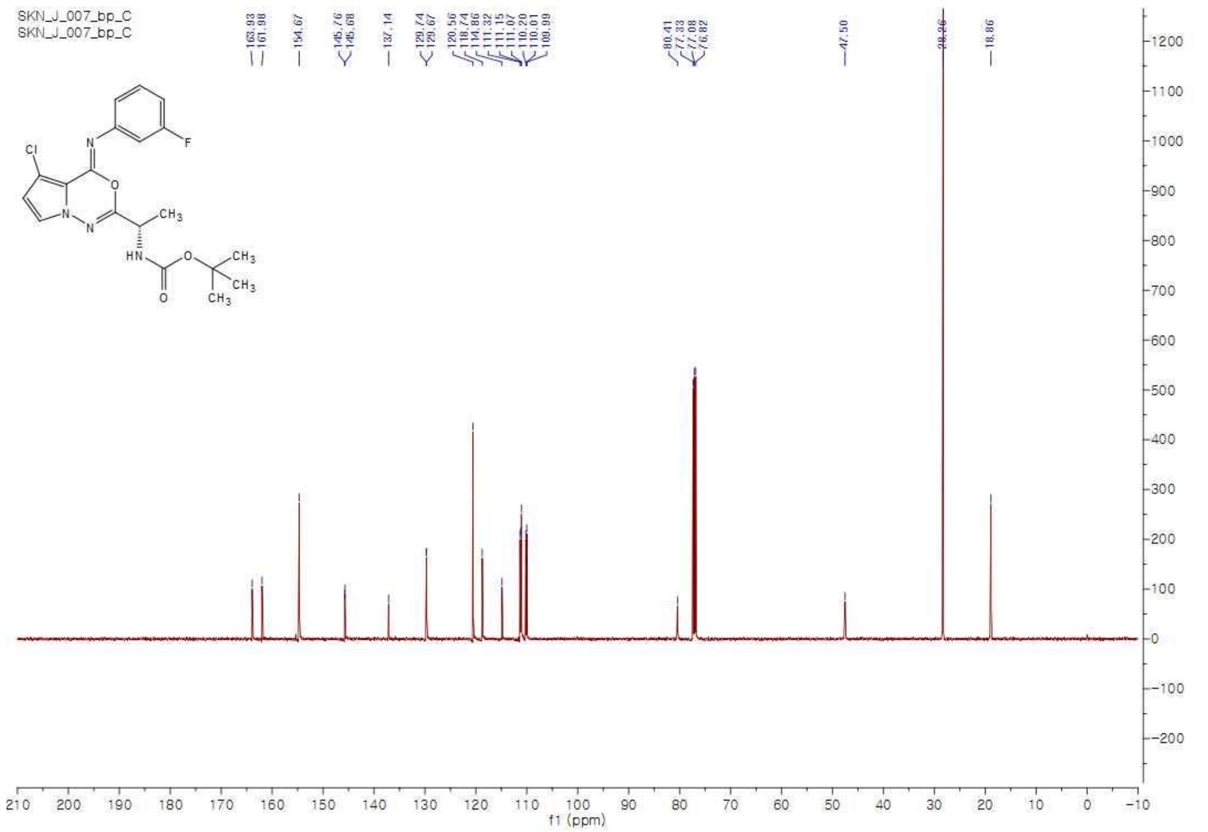

^1H and ^{13}C NMR of compound **12c**

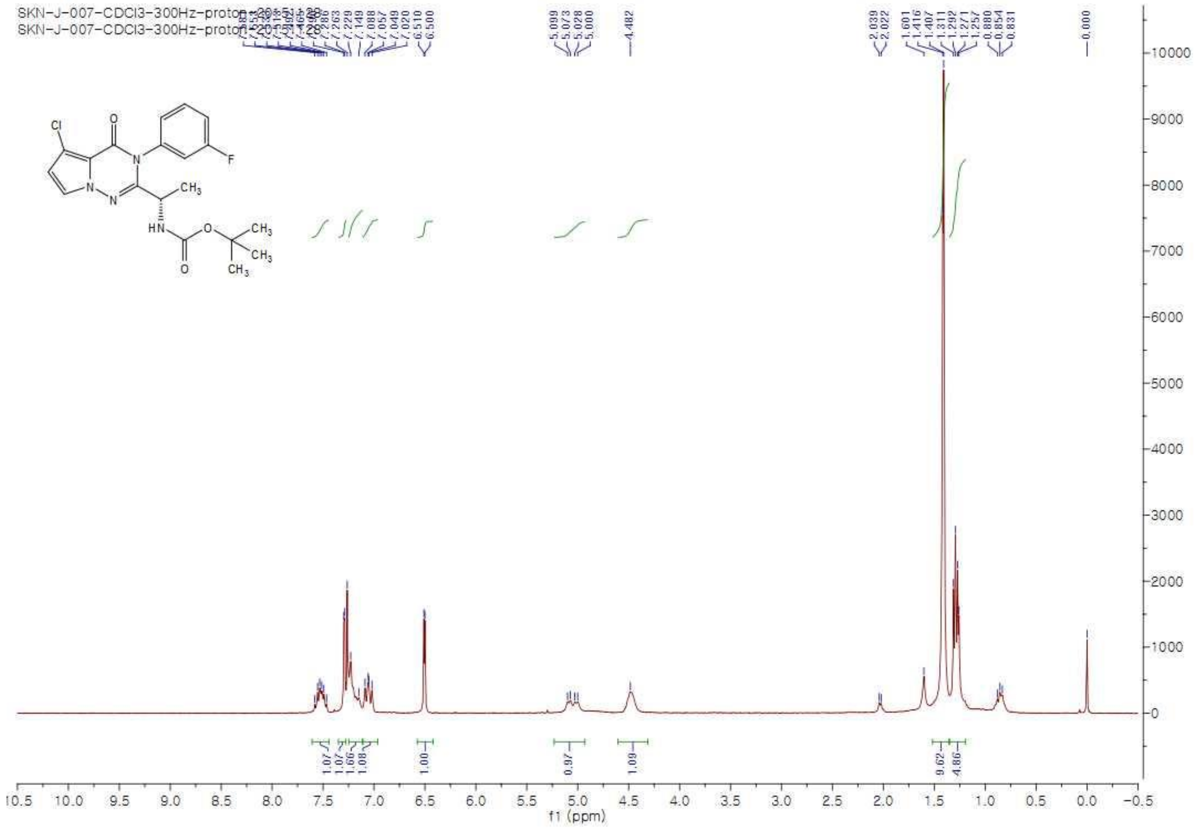

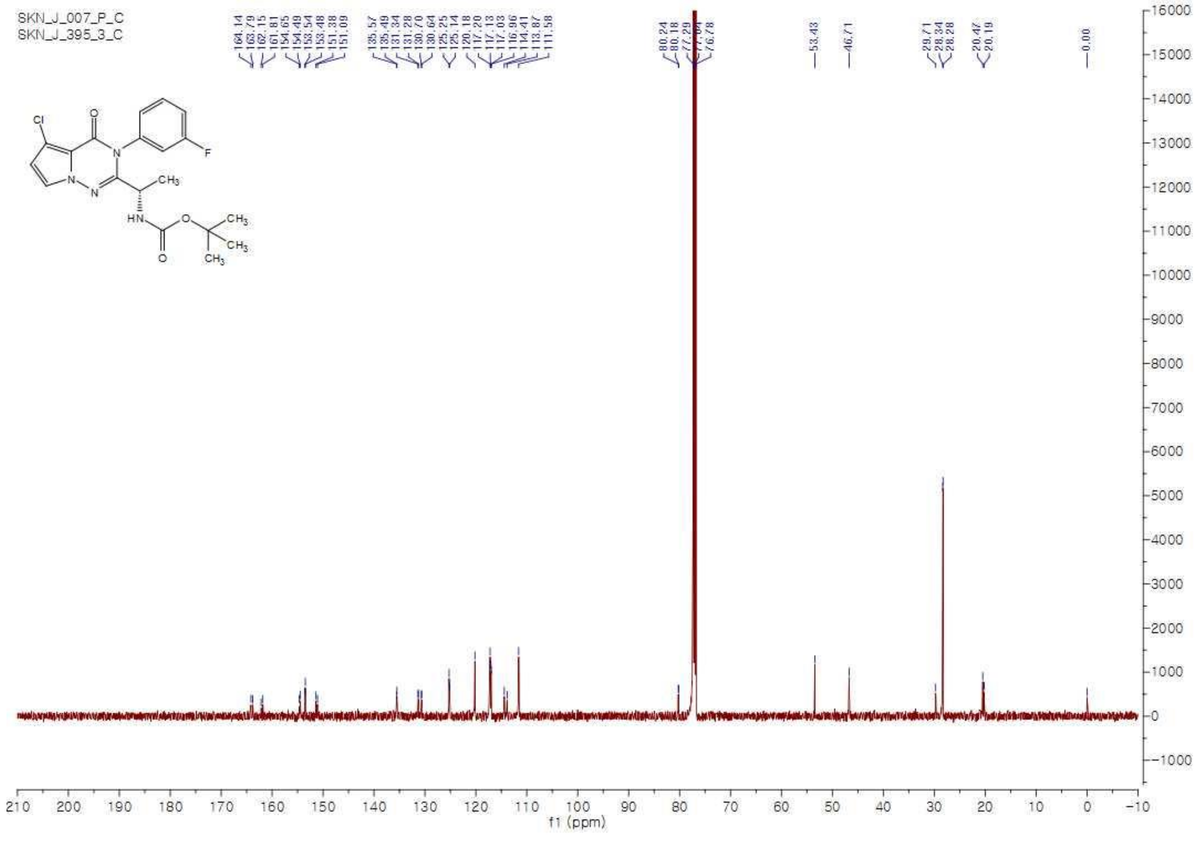

[19]F of **11c** and **12c**

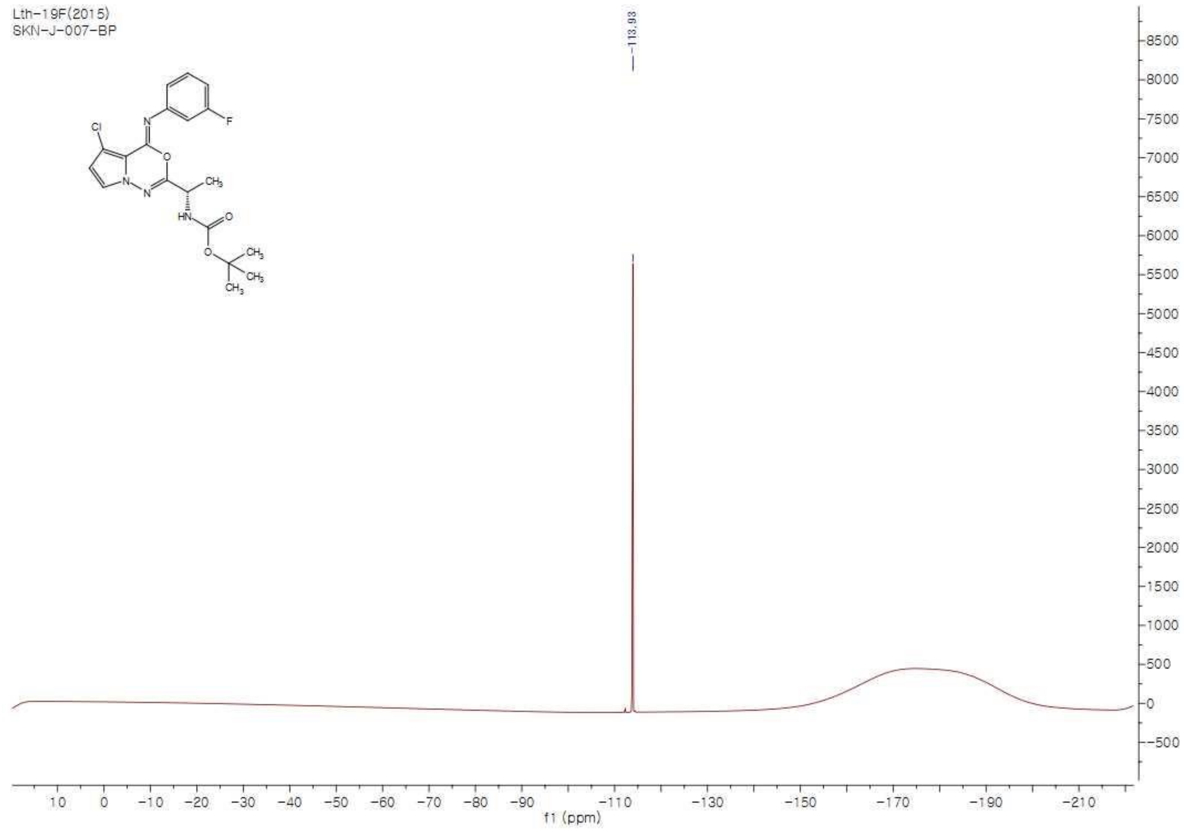

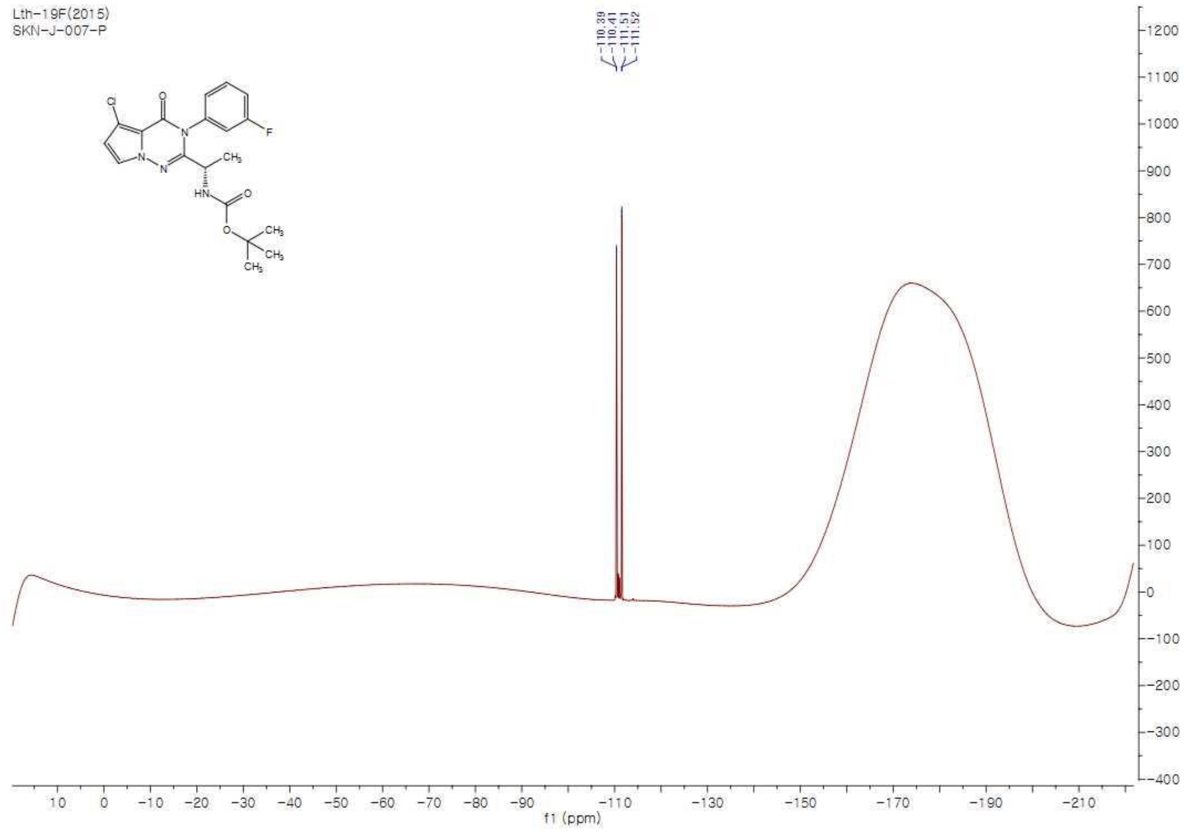

^{1}H and ^{13}C NMR of compound **14d**

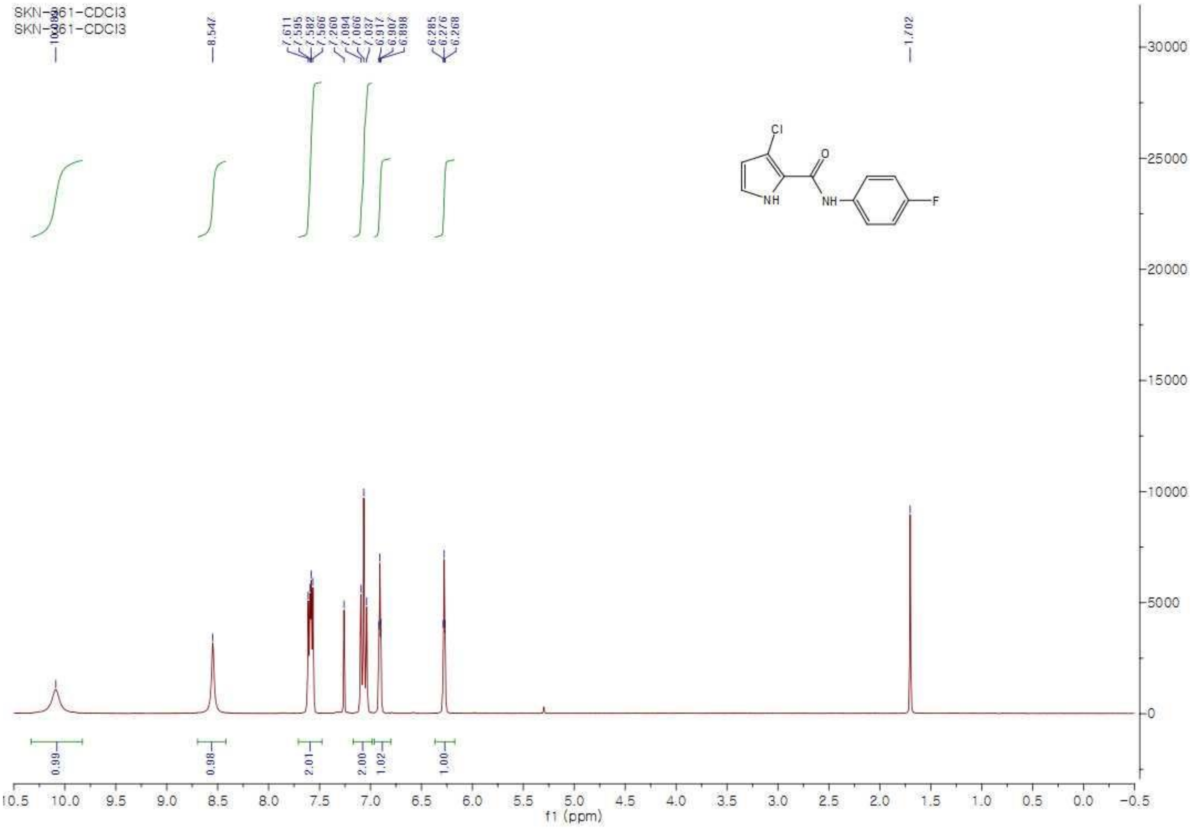

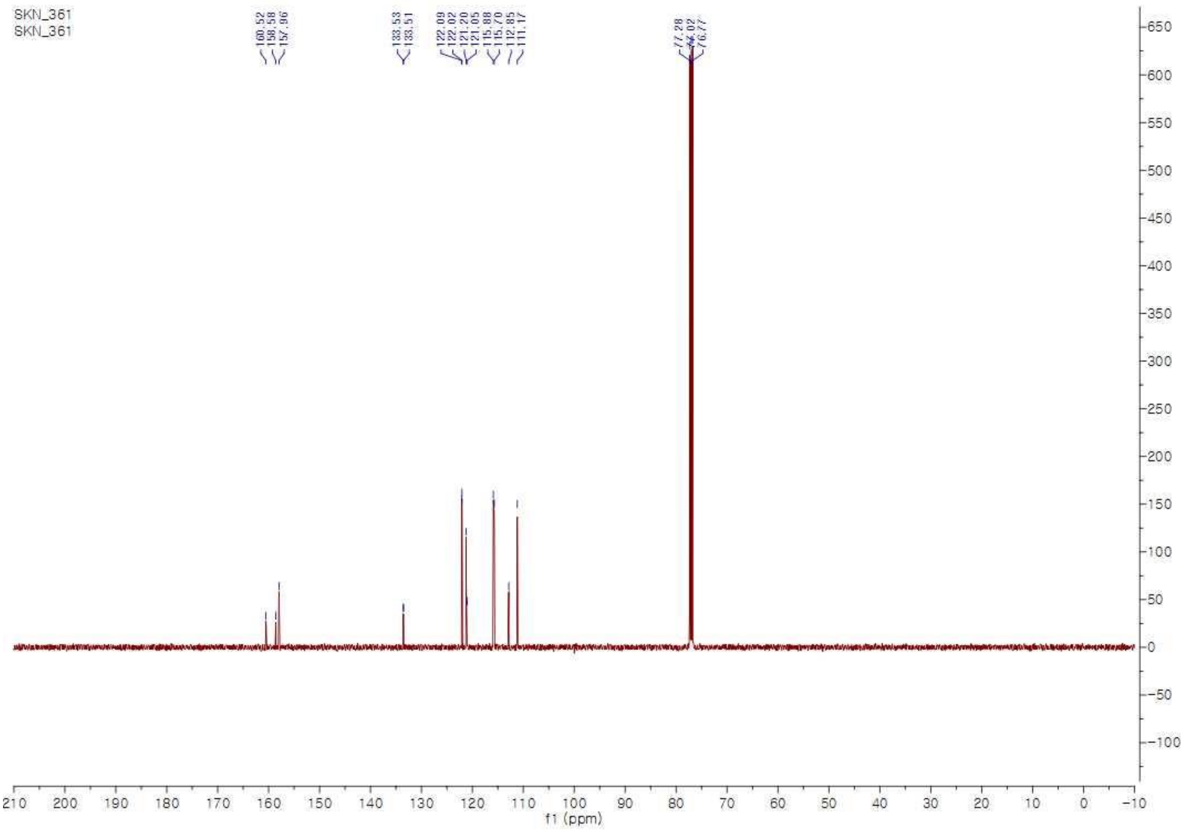

^{1}H and ^{13}C NMR of compound **15d**

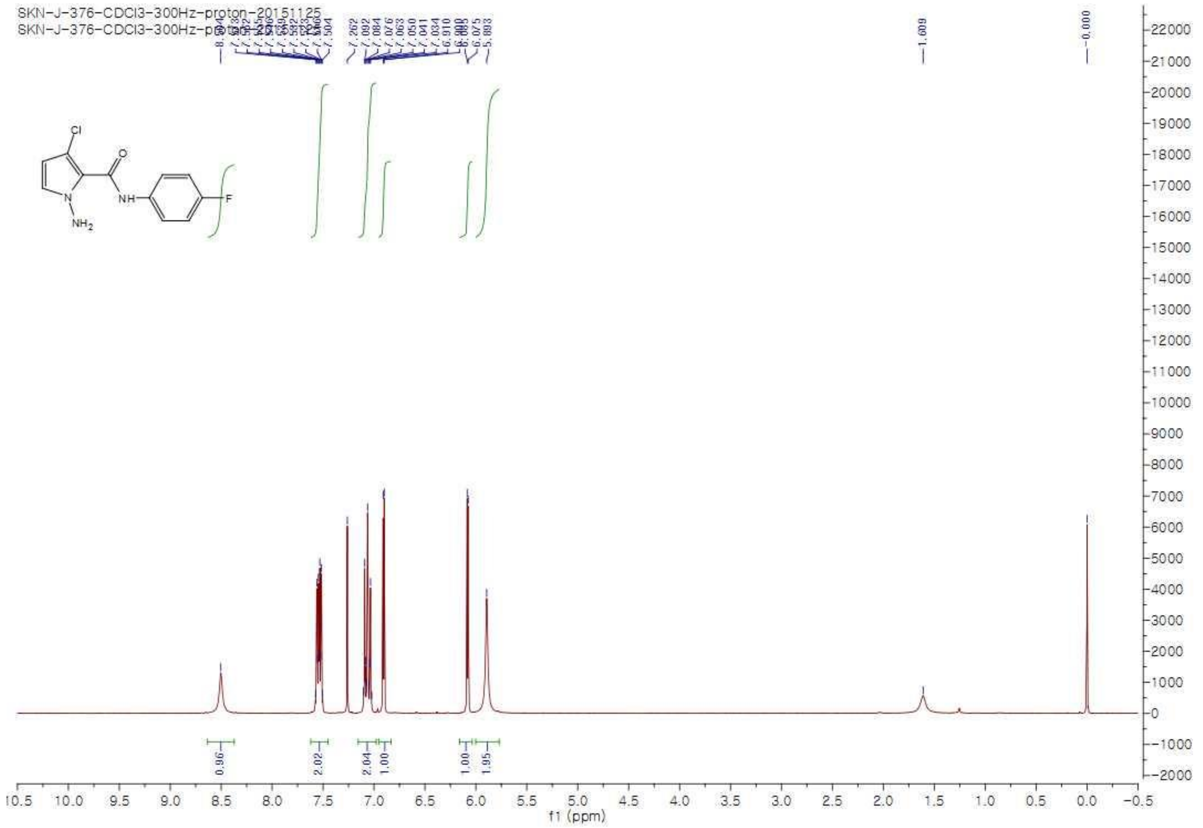

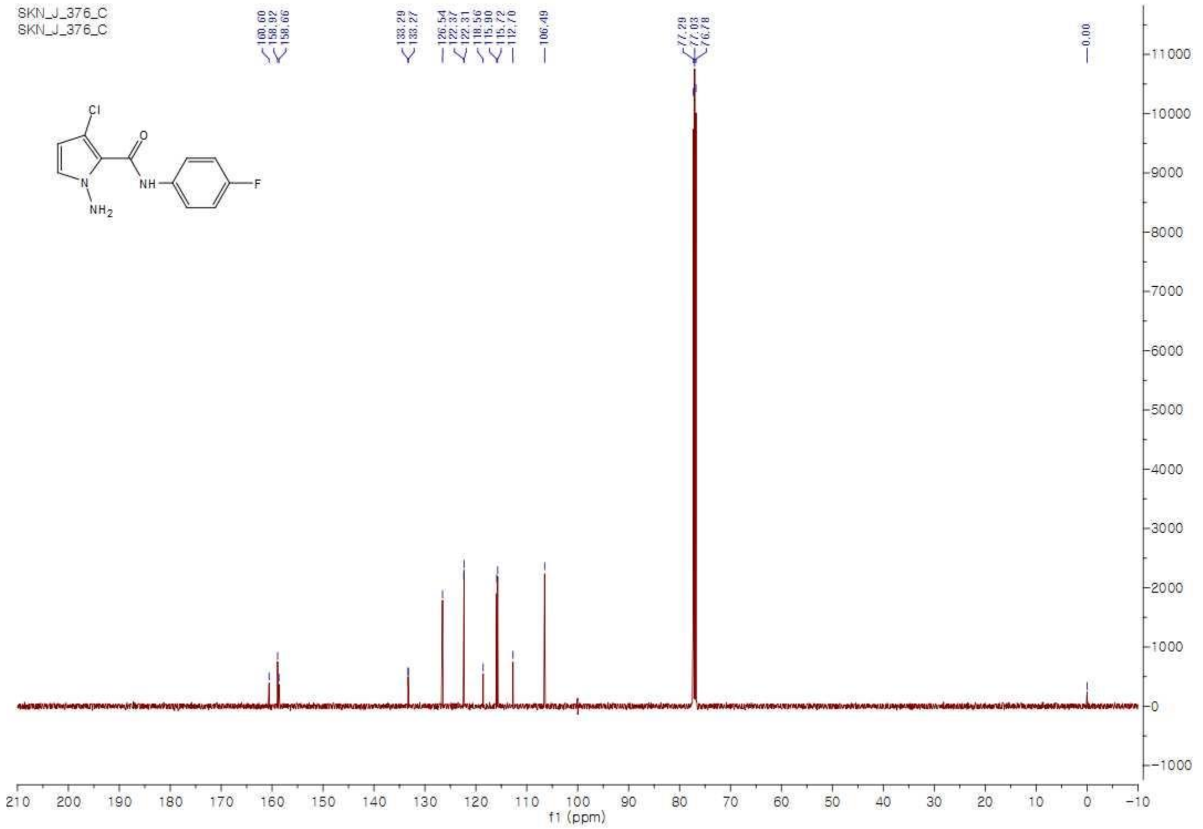

^{1}H and ^{13}C NMR of compound **10d**

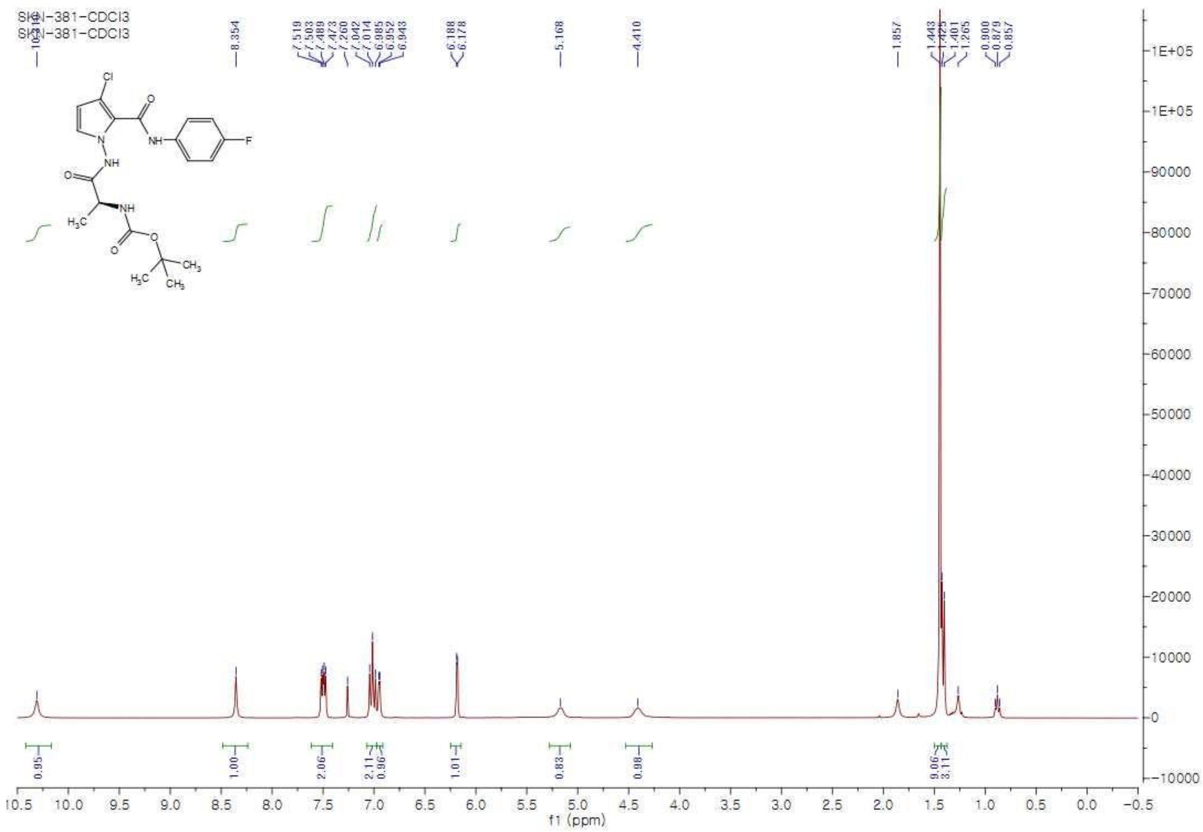

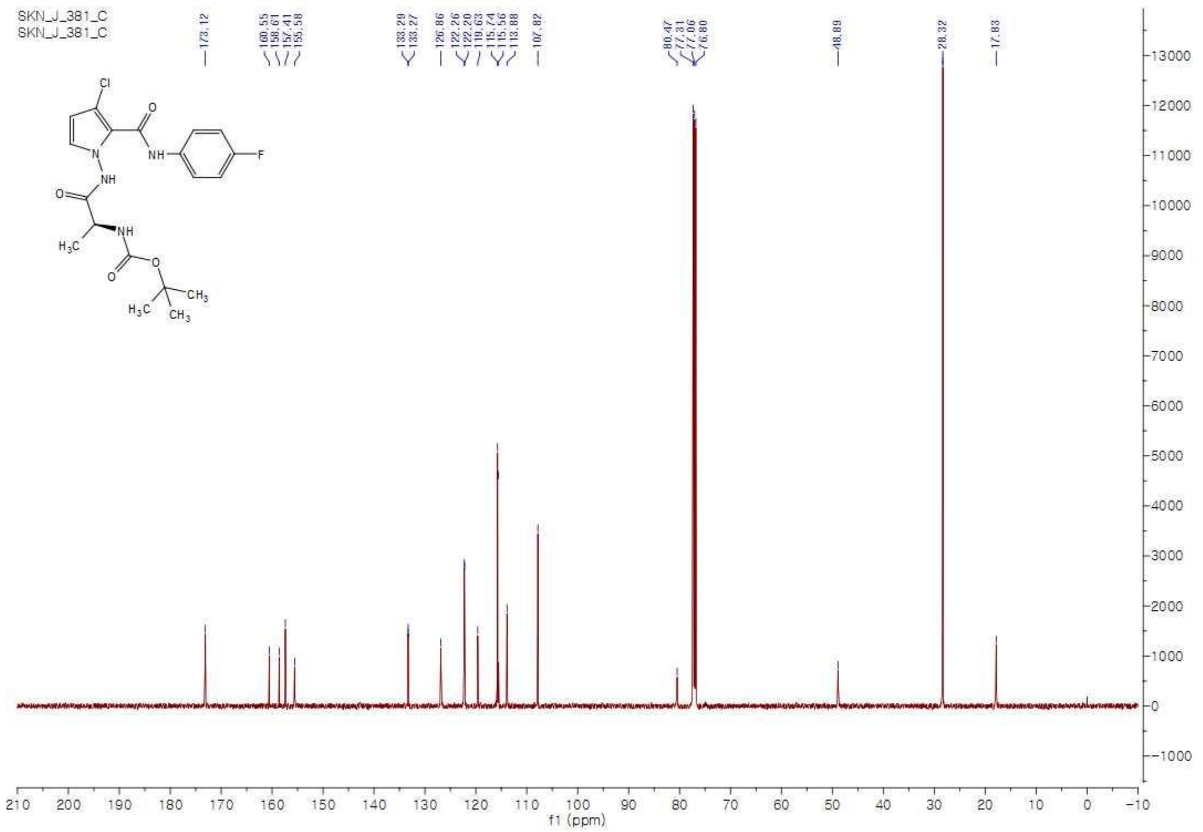

^{1}H and ^{13}C NMR of compound **11d**

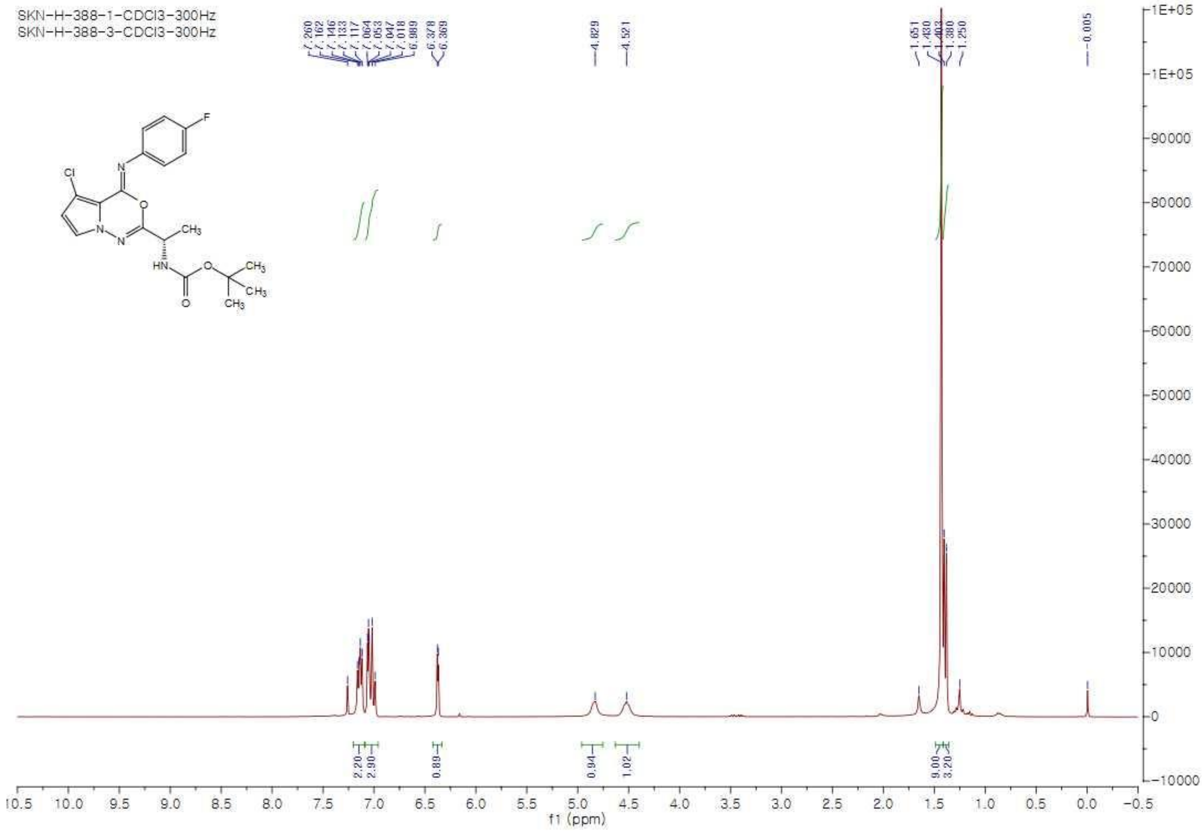

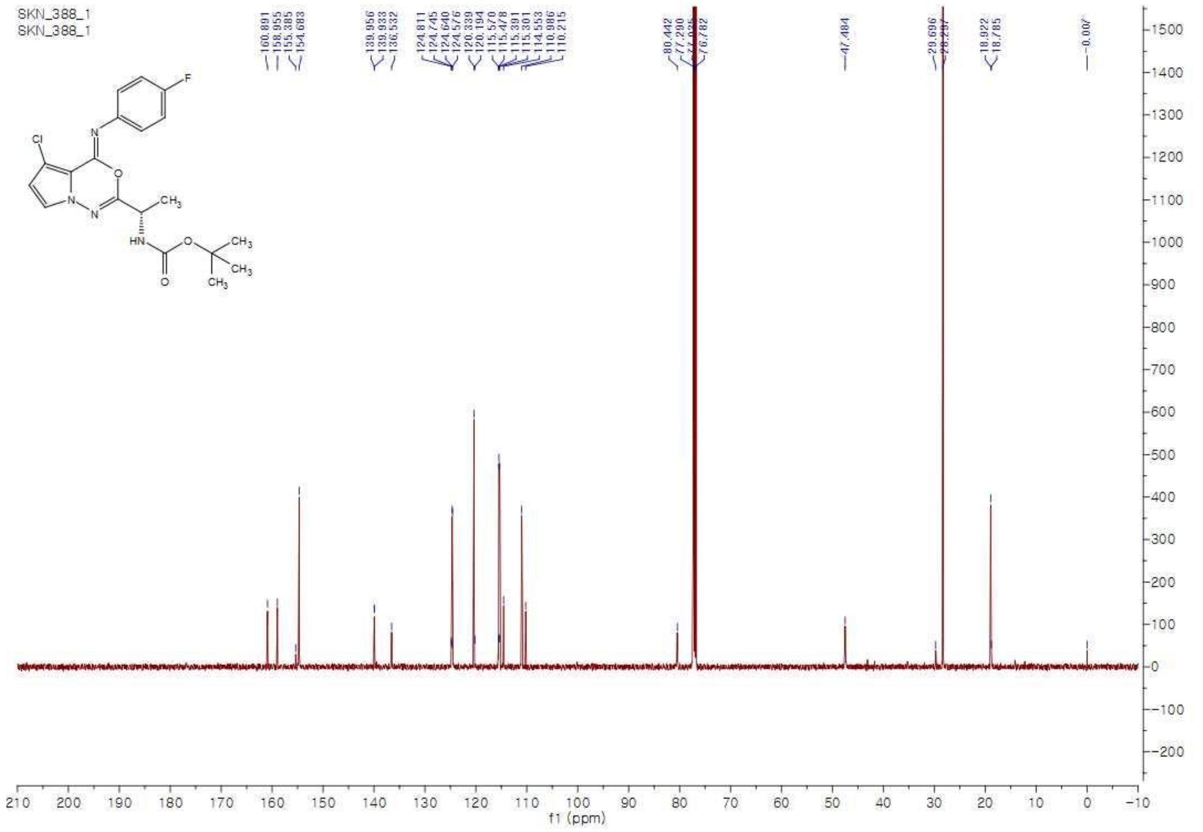

^{1}H and ^{13}C NMR of compound **12d**

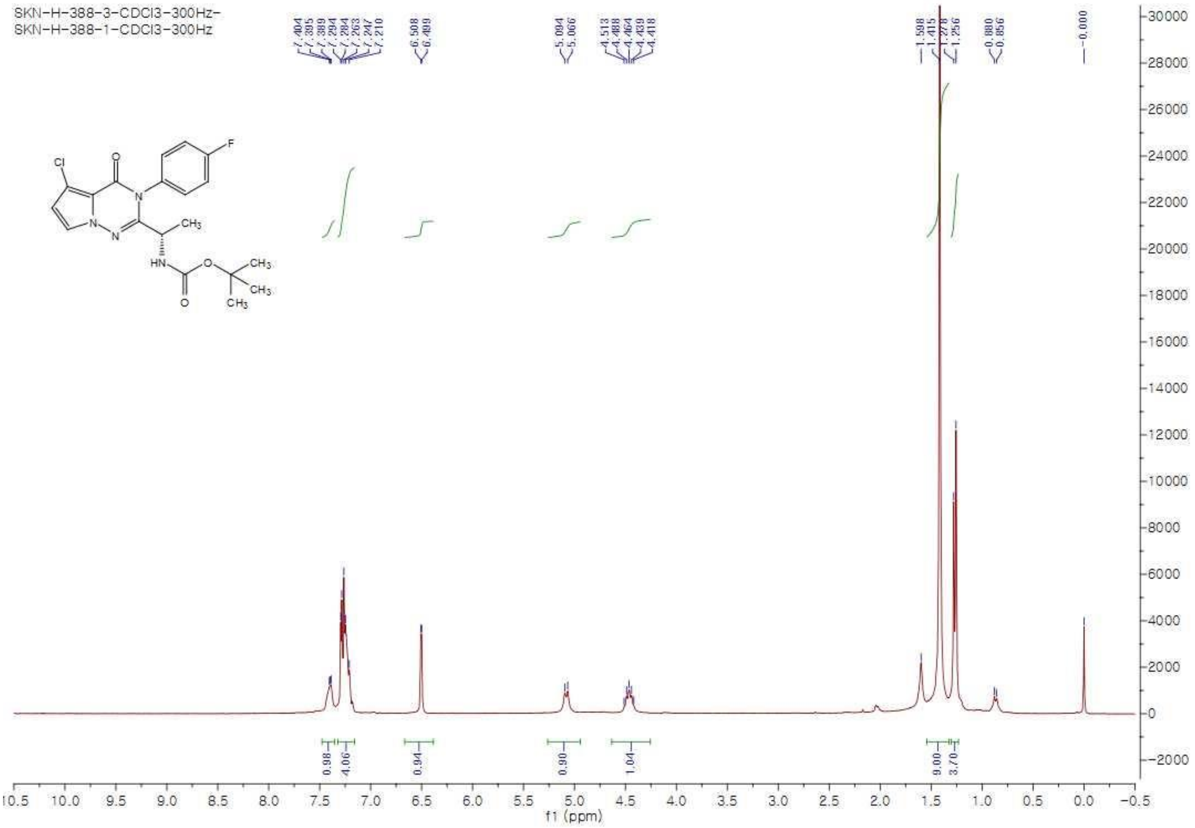

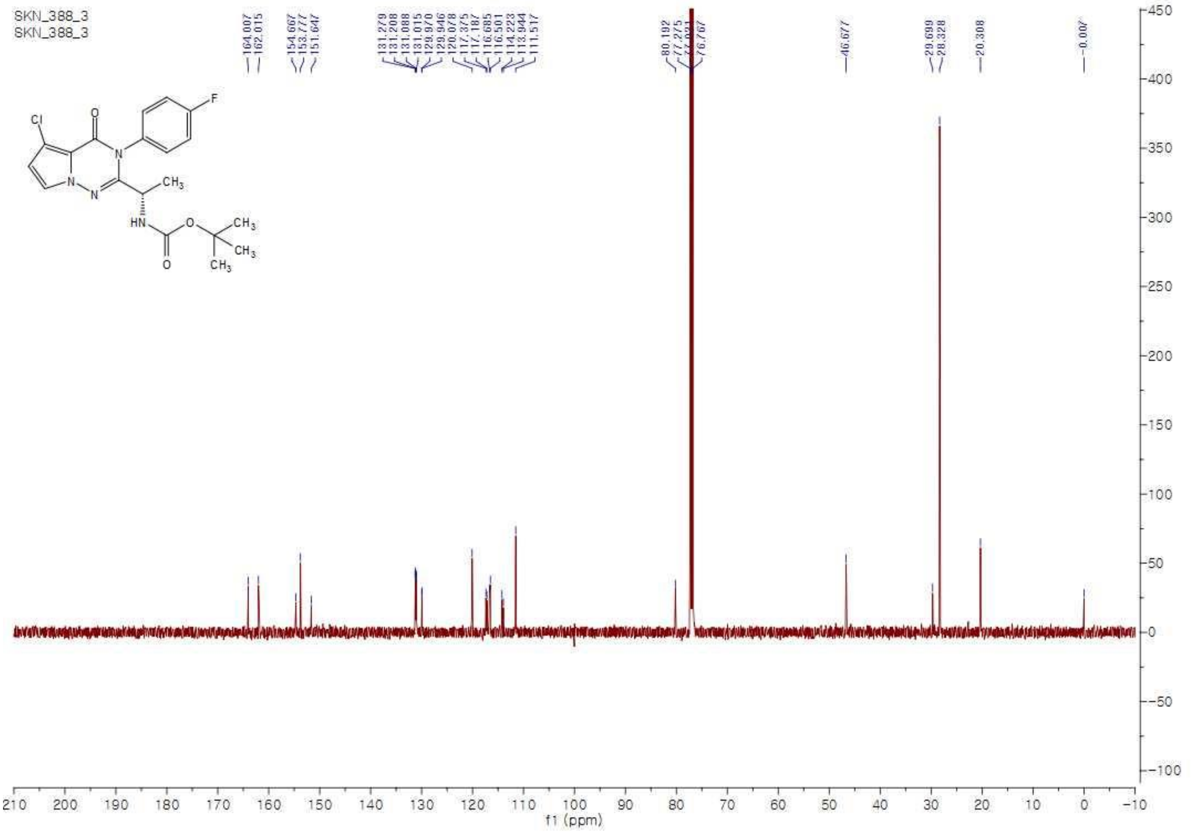

^{19}F NMR of compound **11d** and **12d**

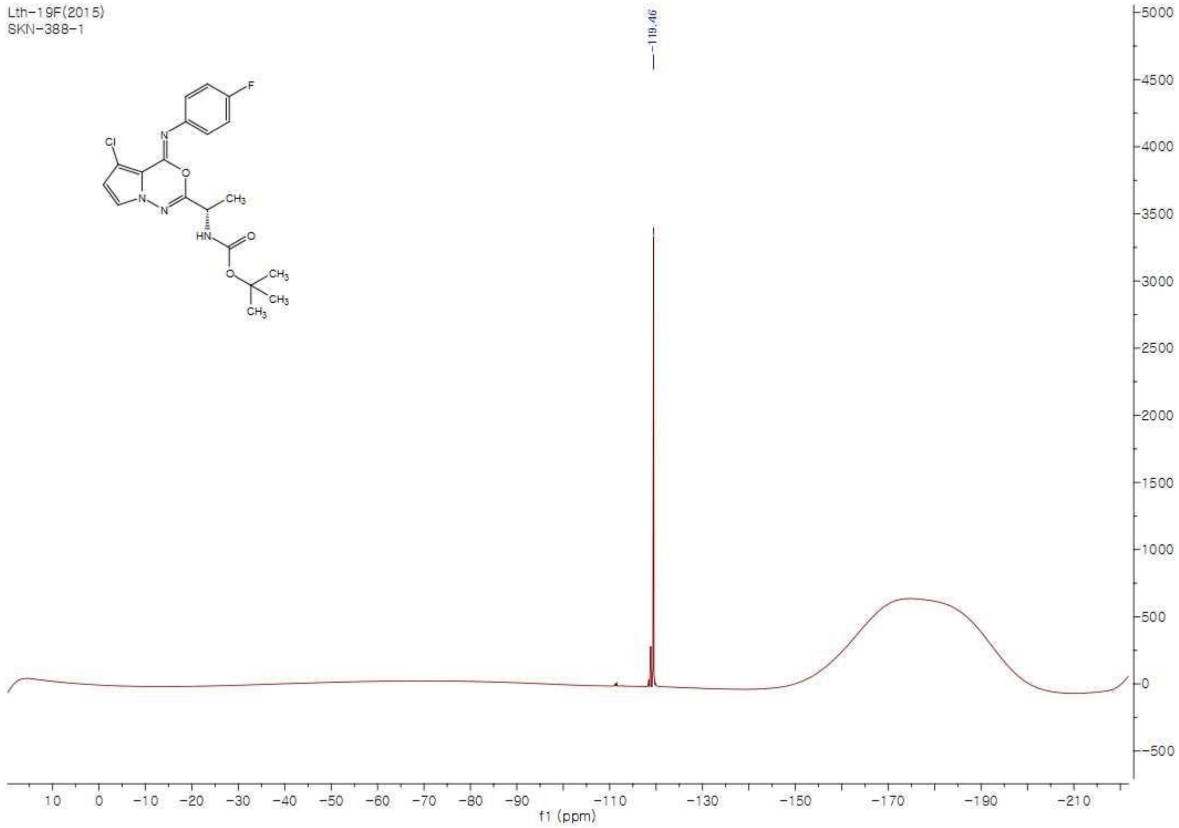

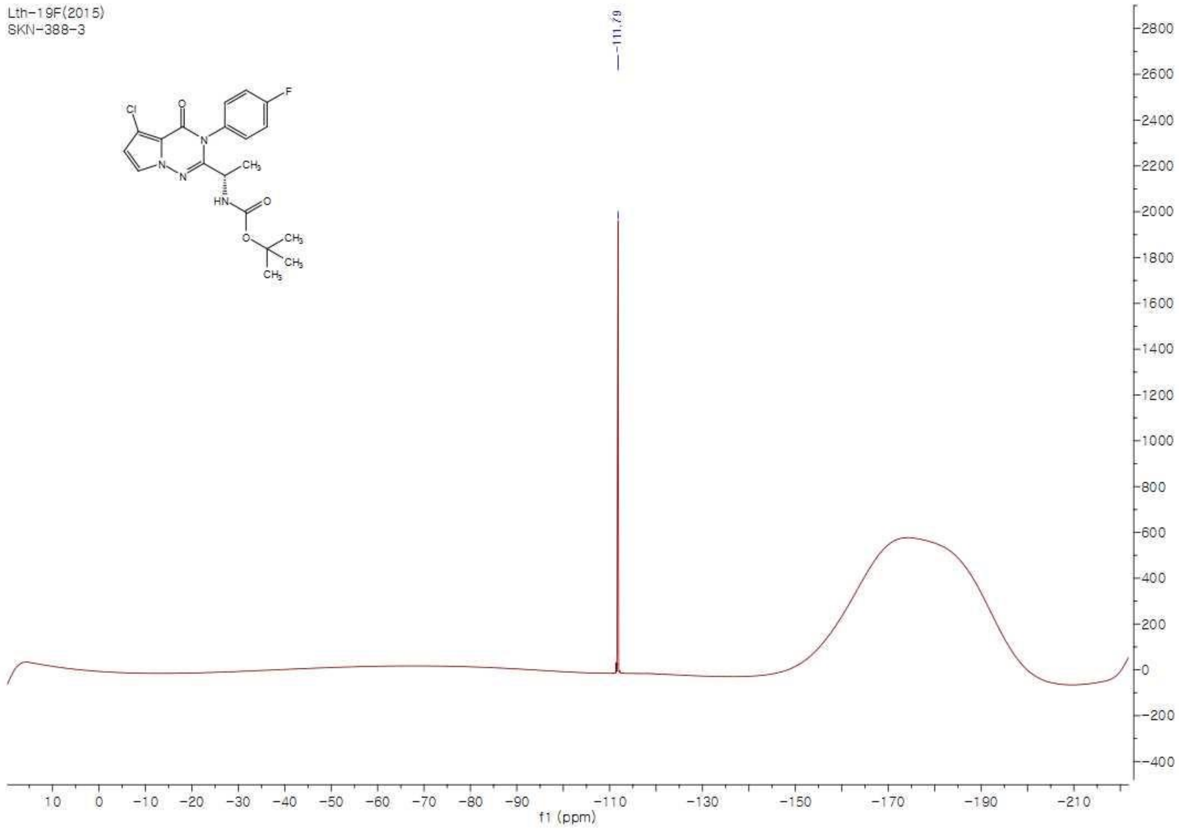

^{1}H and ^{13}C NMR of compound **14e**

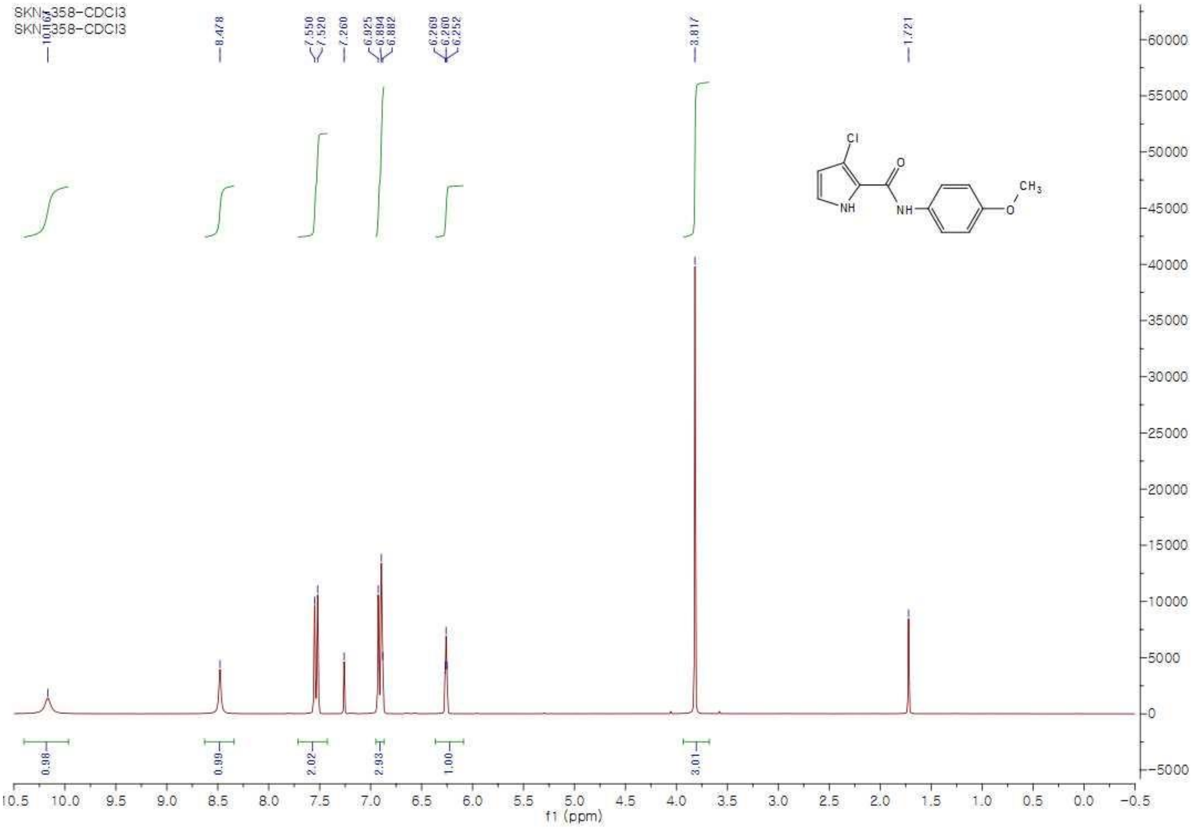

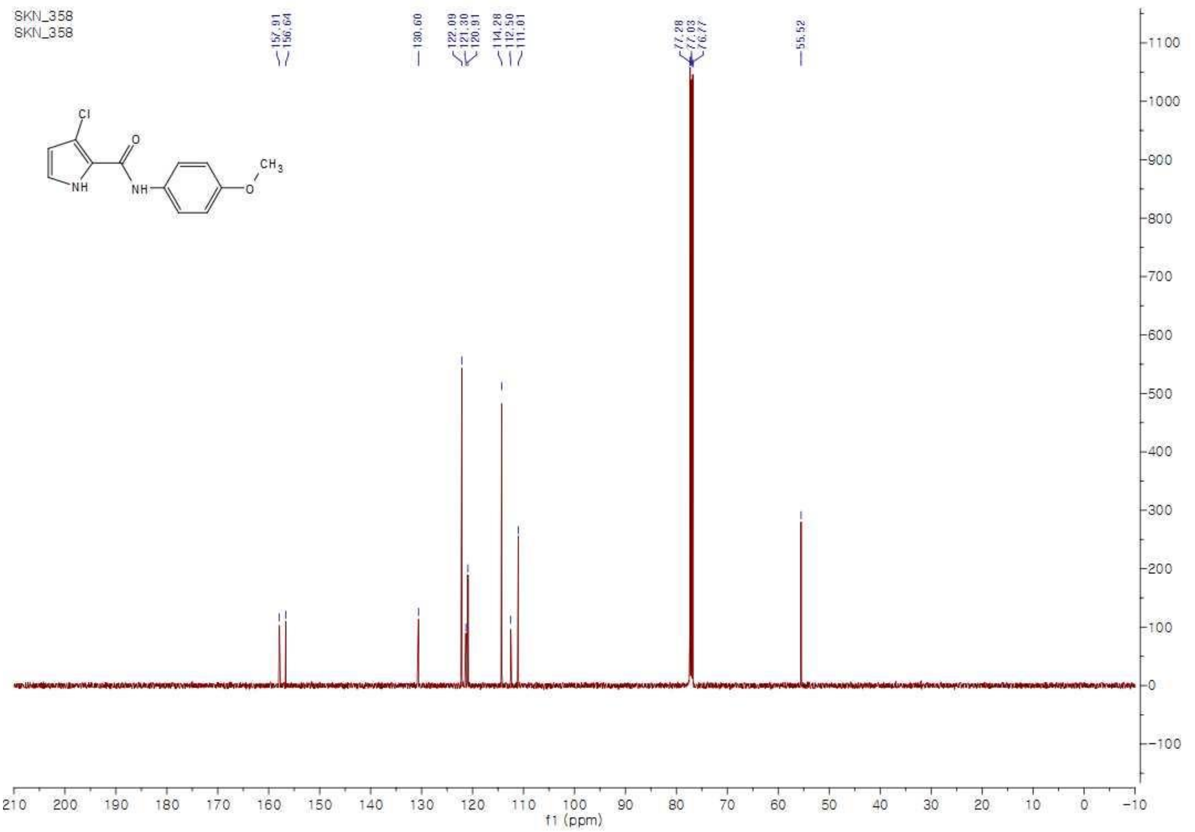

^{1}H and ^{13}C NMR of compound **15e**

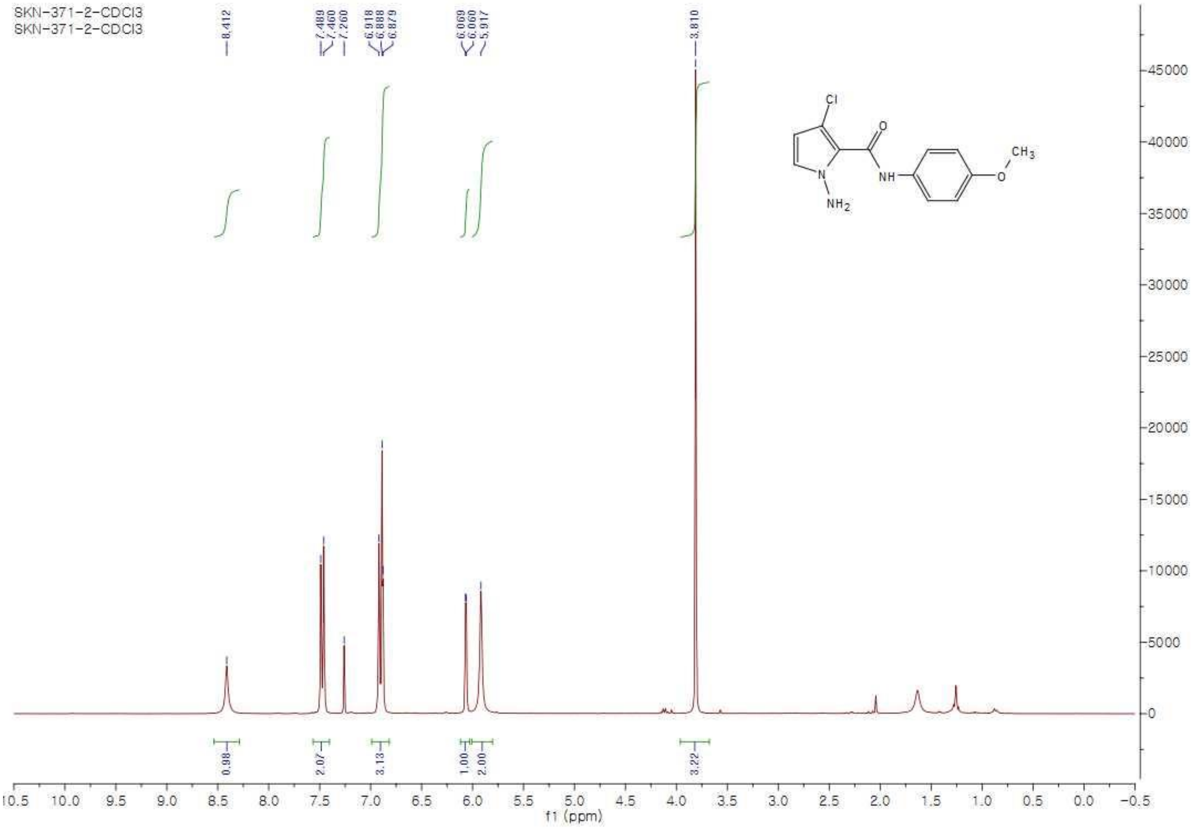

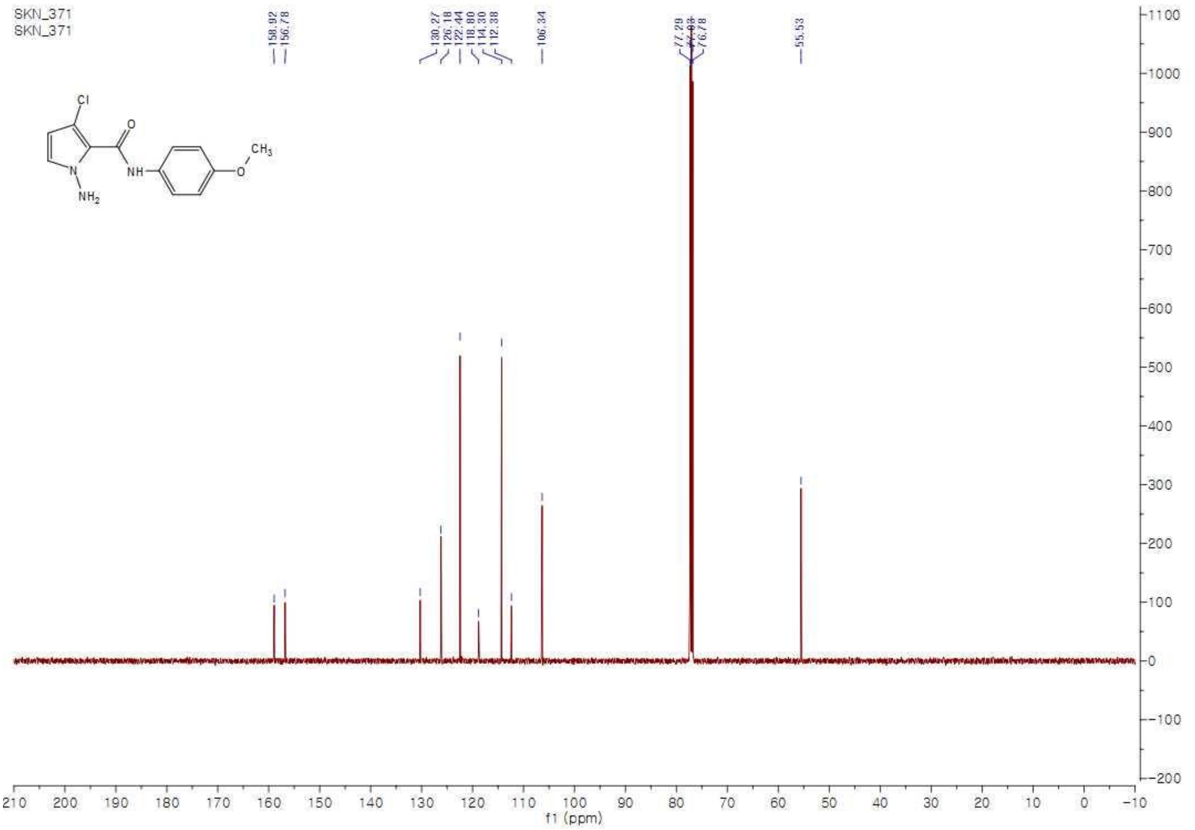

^{1}H and ^{13}C NMR of compound **10e**

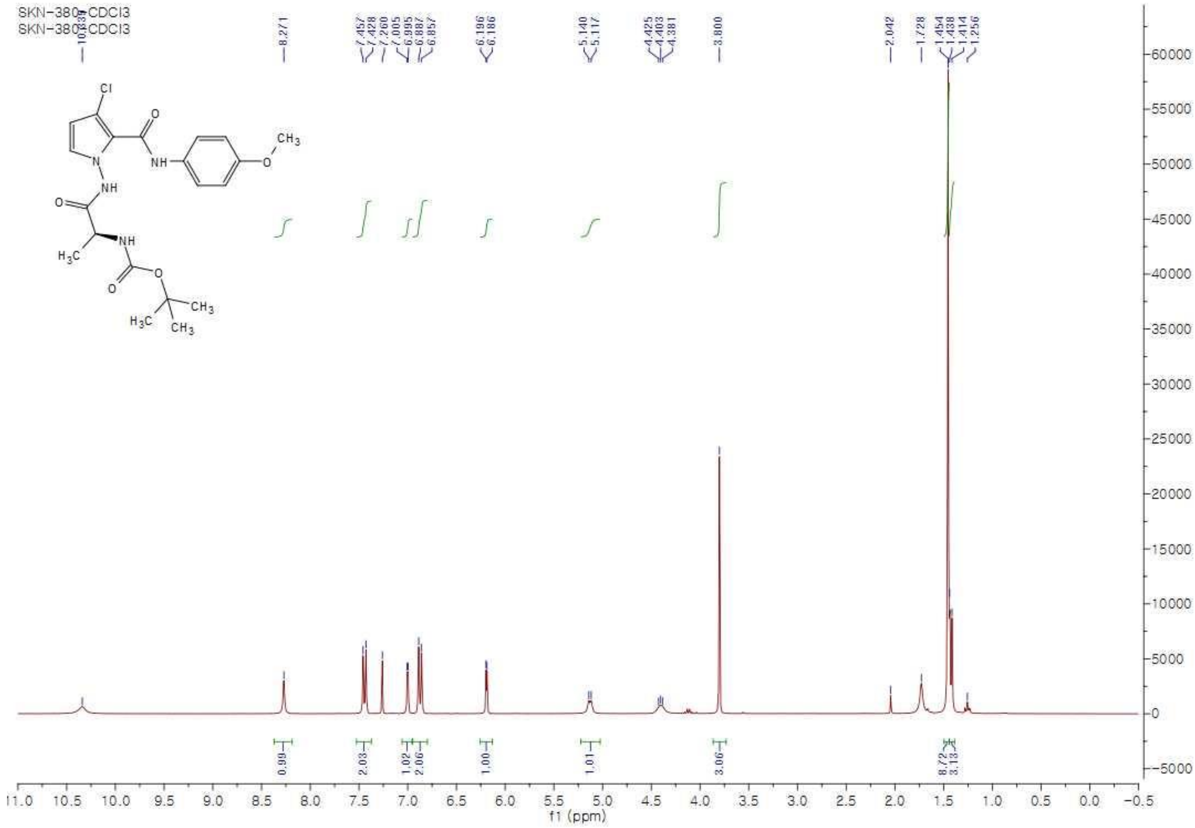

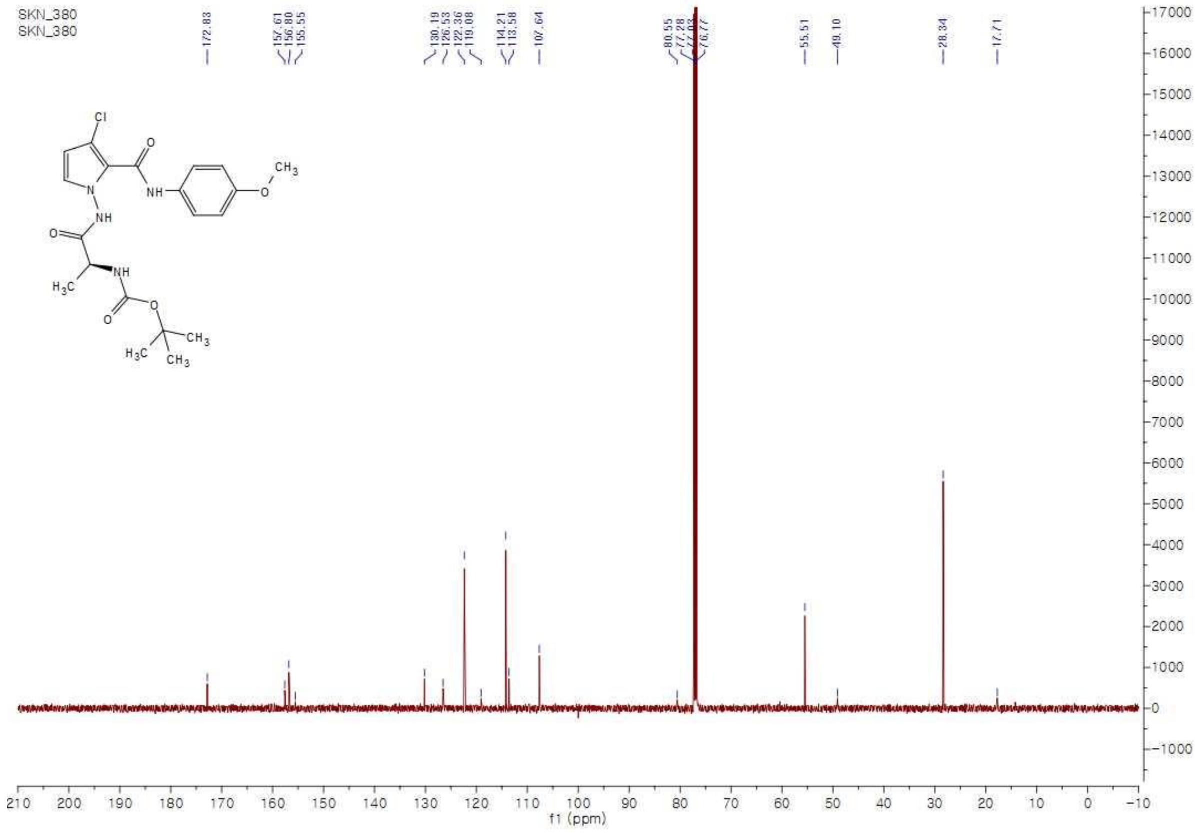

^{1}H and ^{13}C NMR of compound **11e**

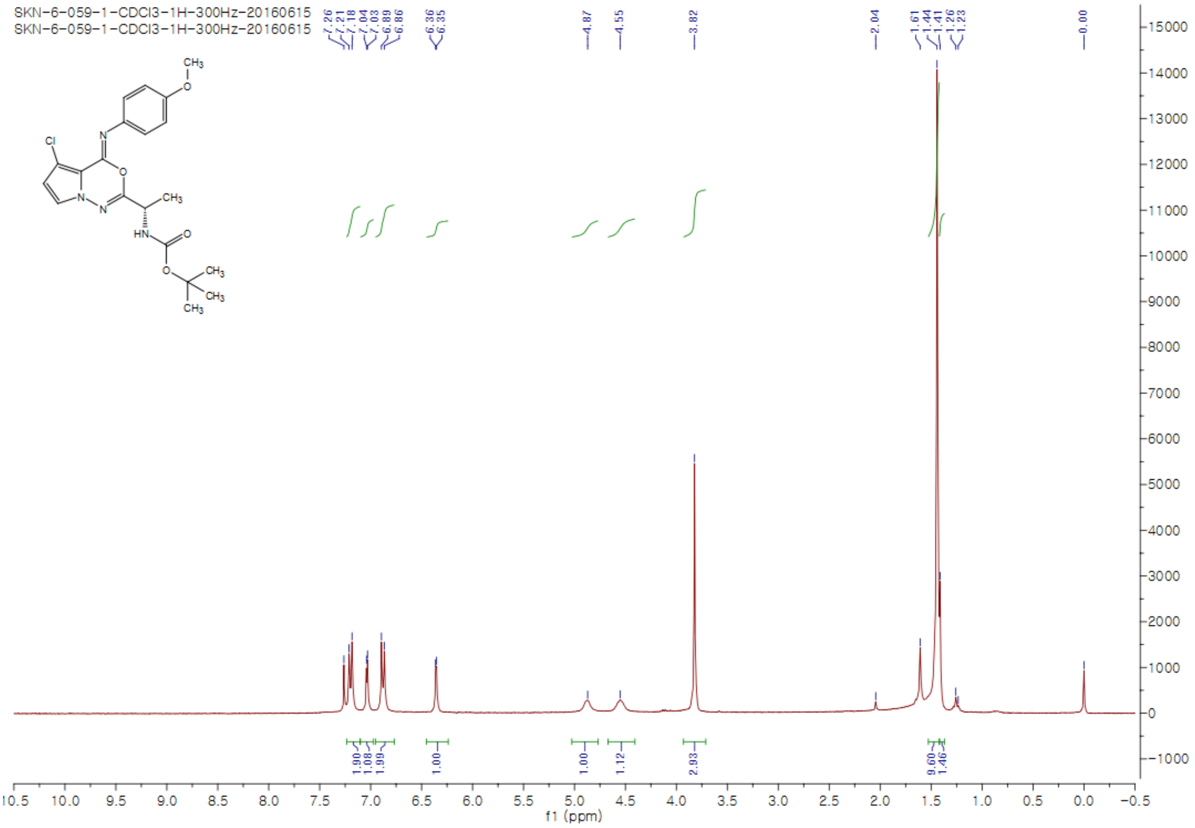

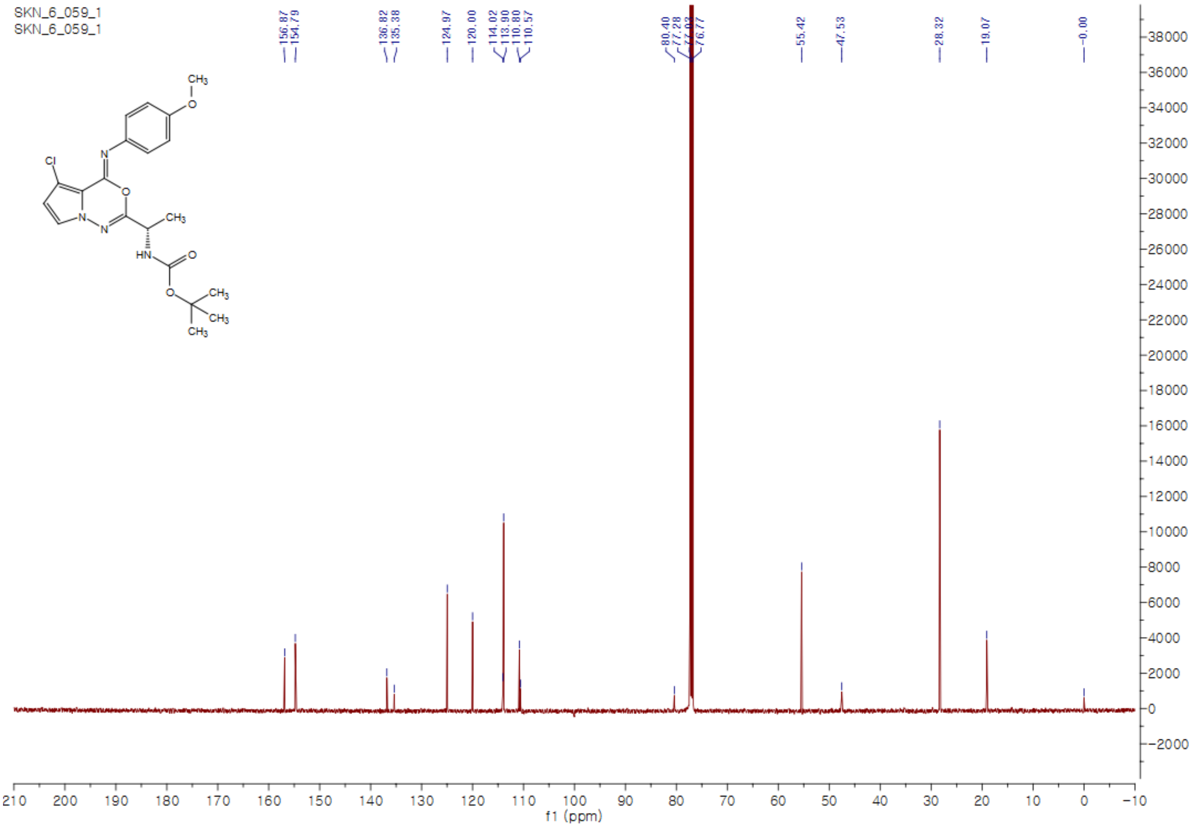

^{1}H and ^{13}C NMR of compound **12e**

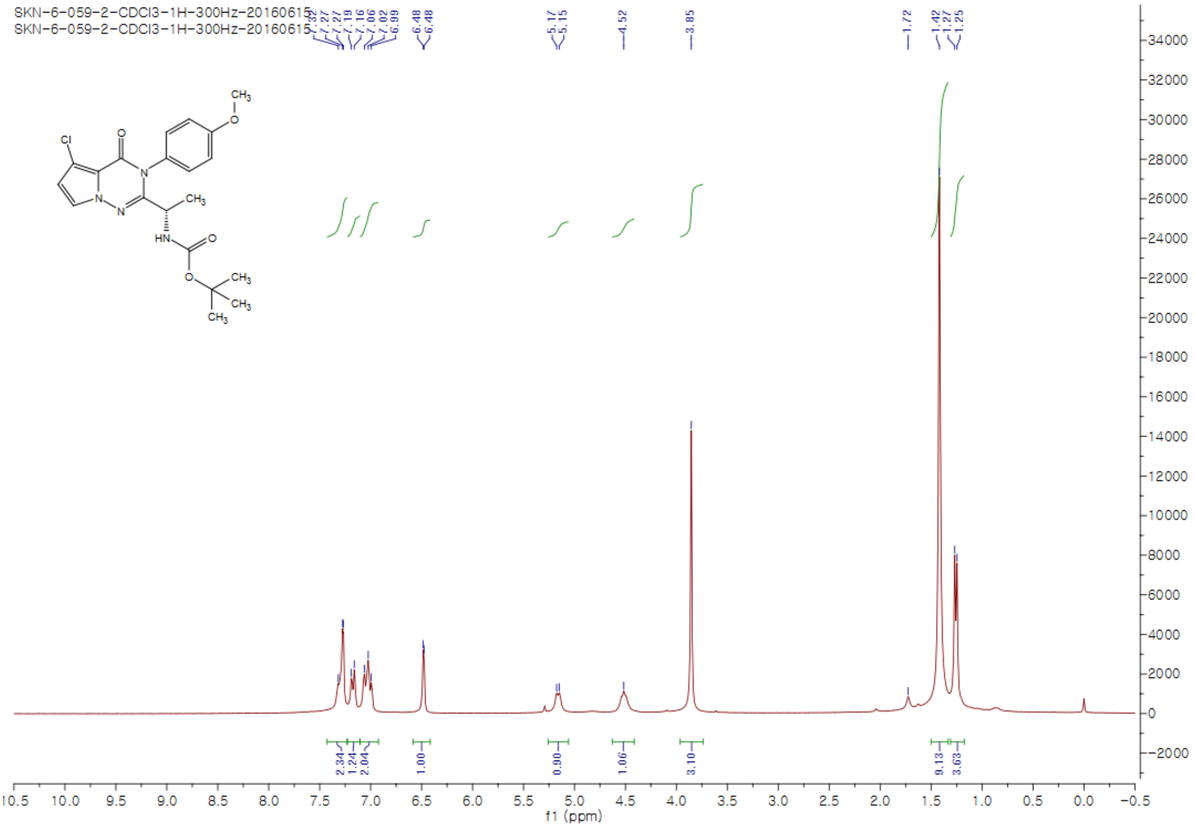

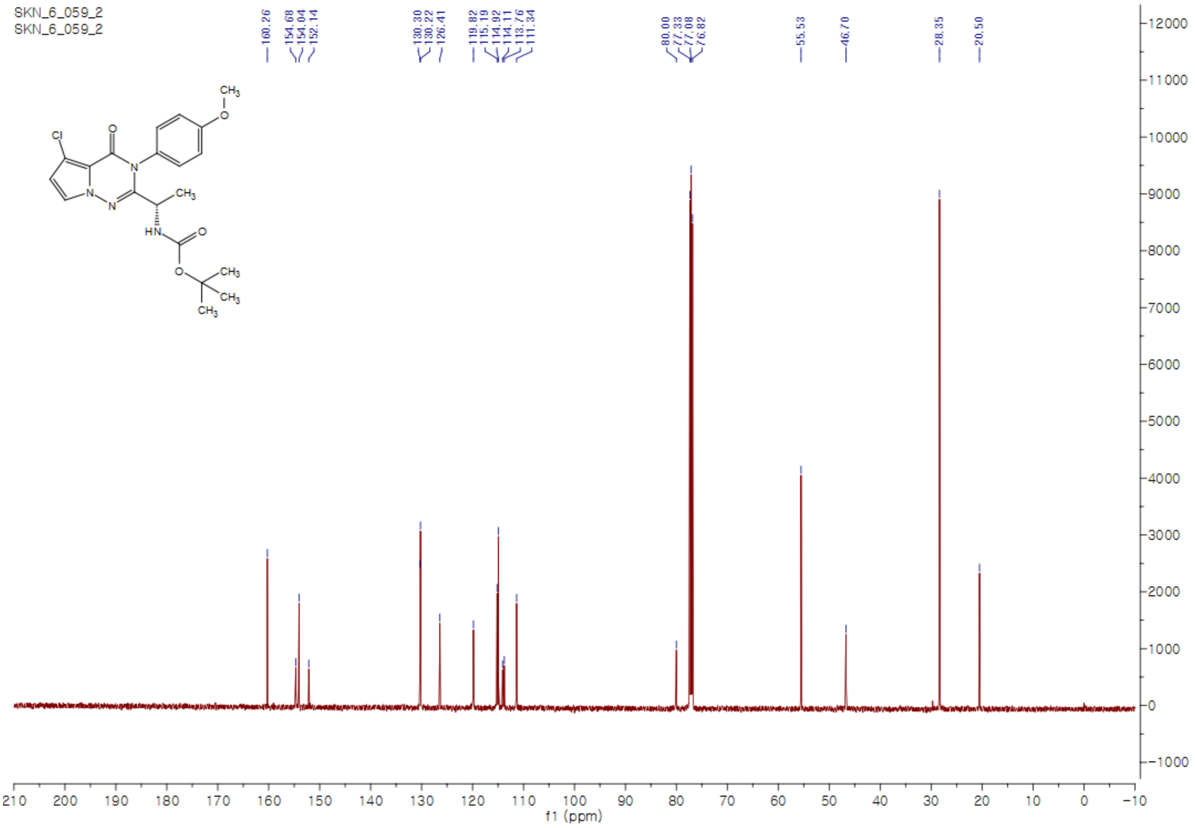

^{1}H and ^{13}C NMR of compound **14f**

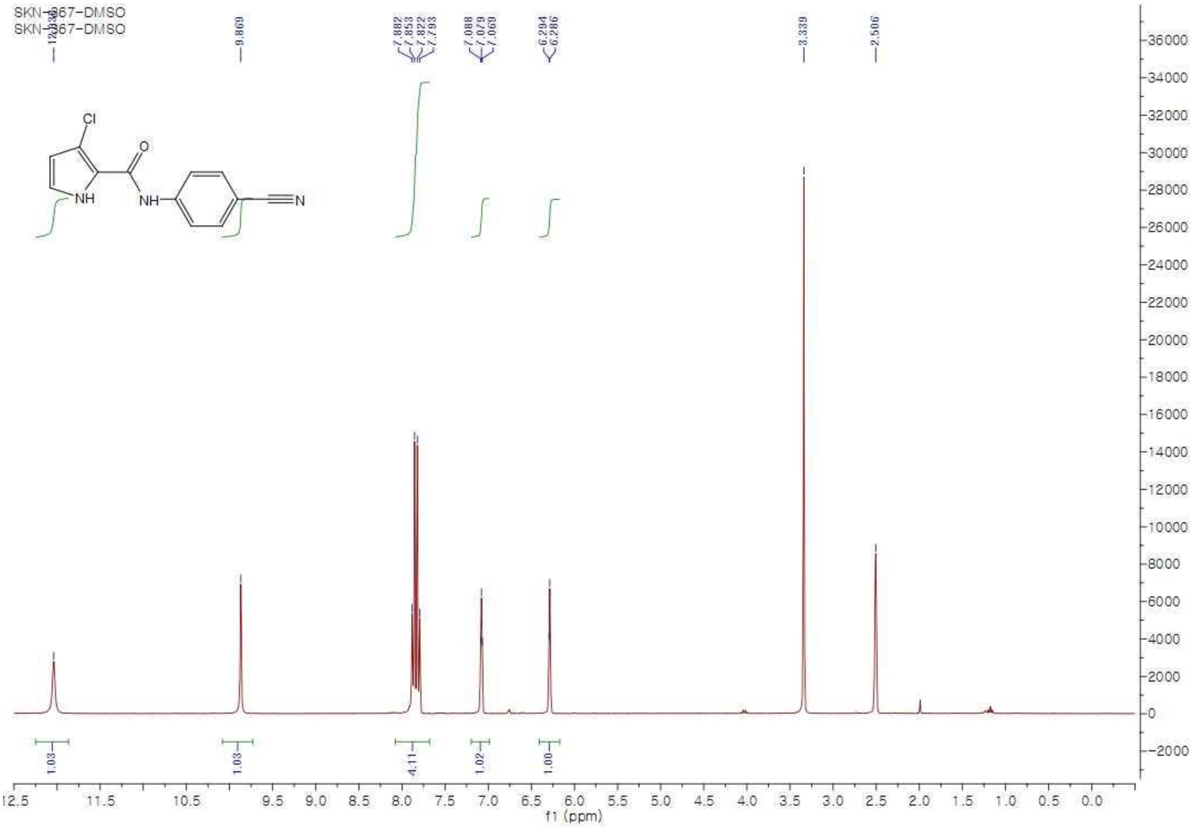

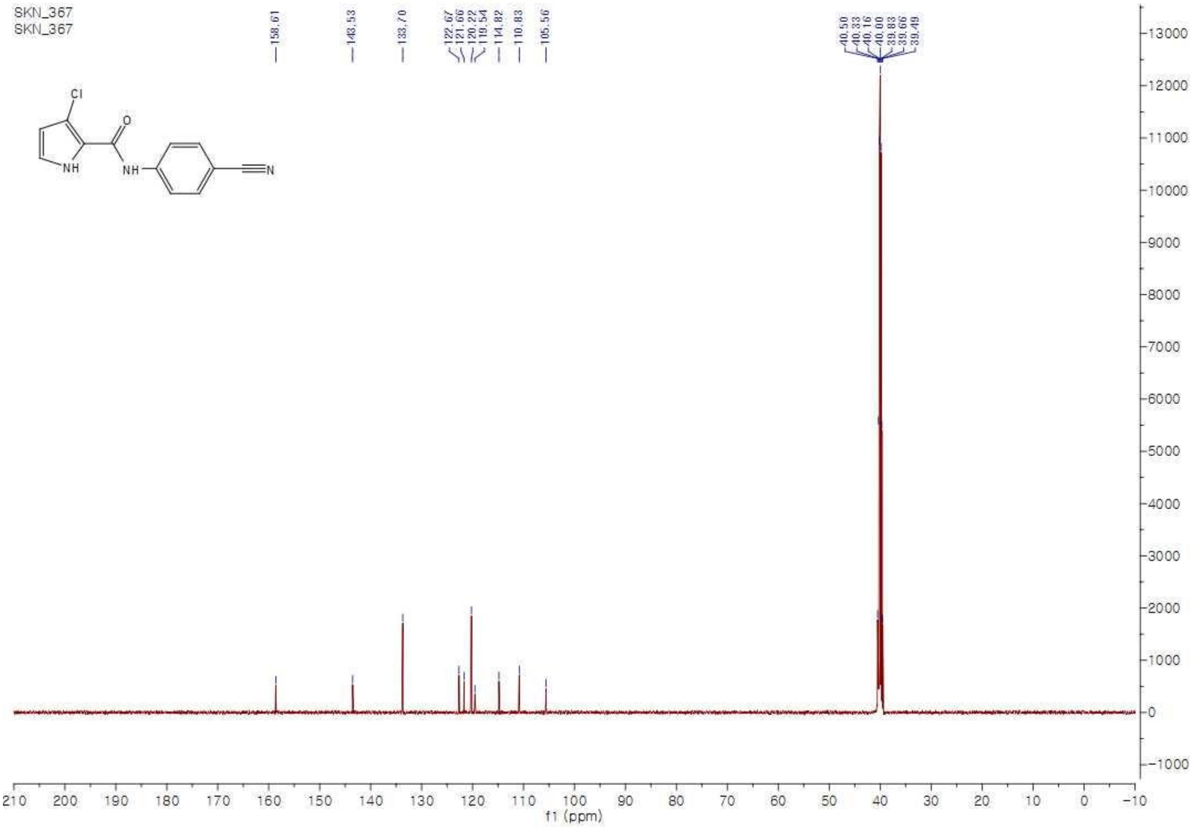

^{1}H and ^{13}C NMR of compound **15f**

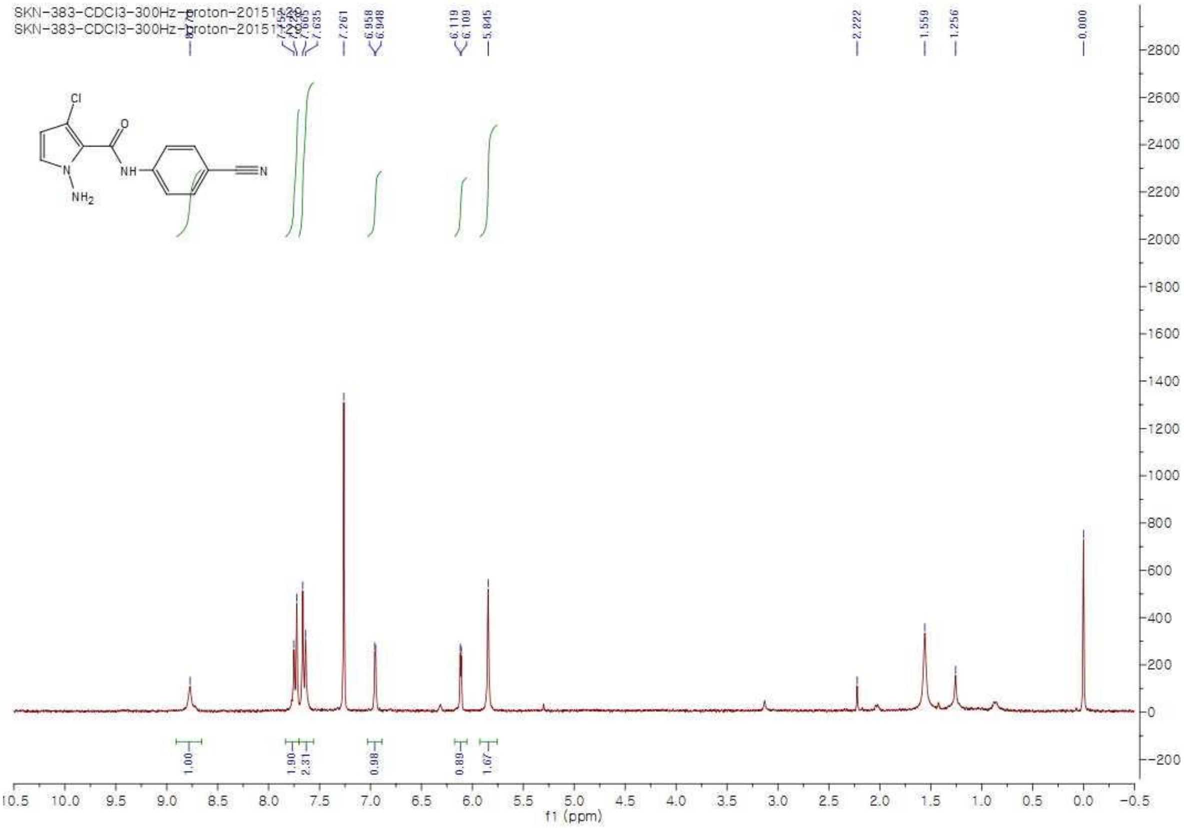

^{1}H and ^{13}C NMR of compound **10f**

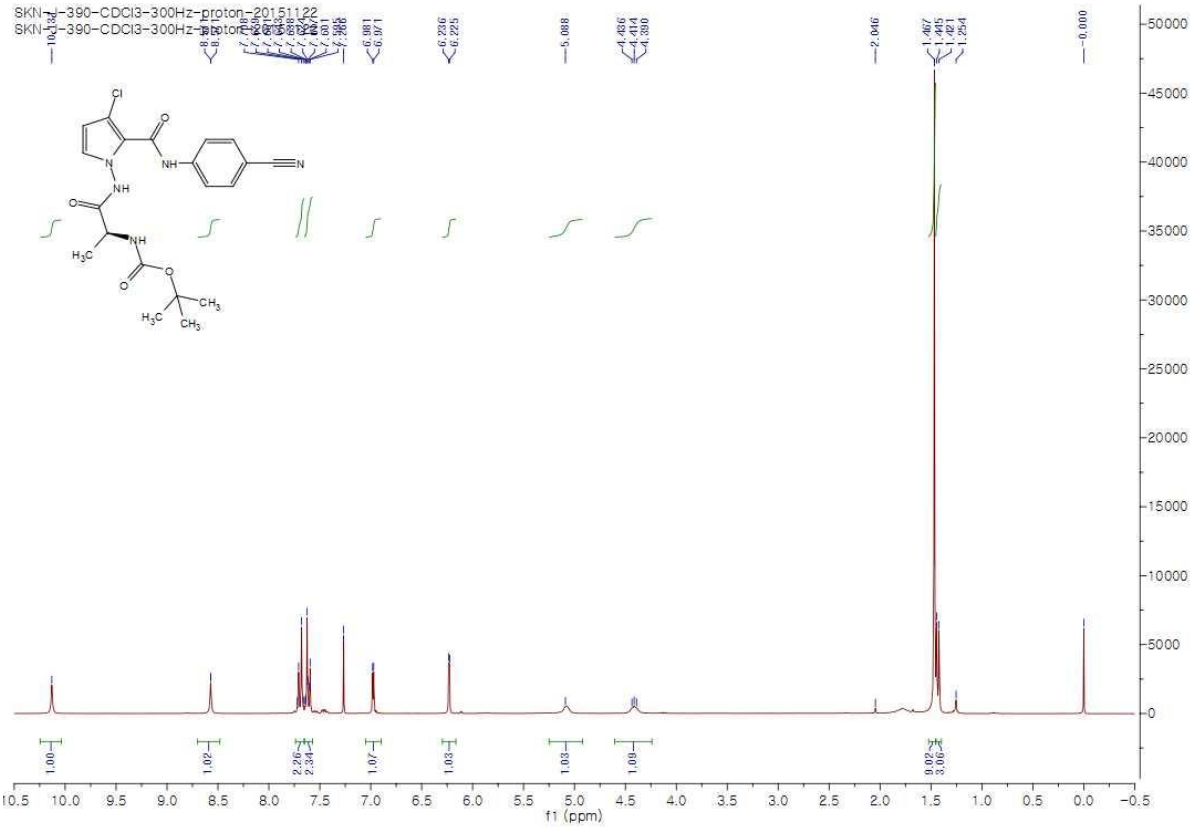

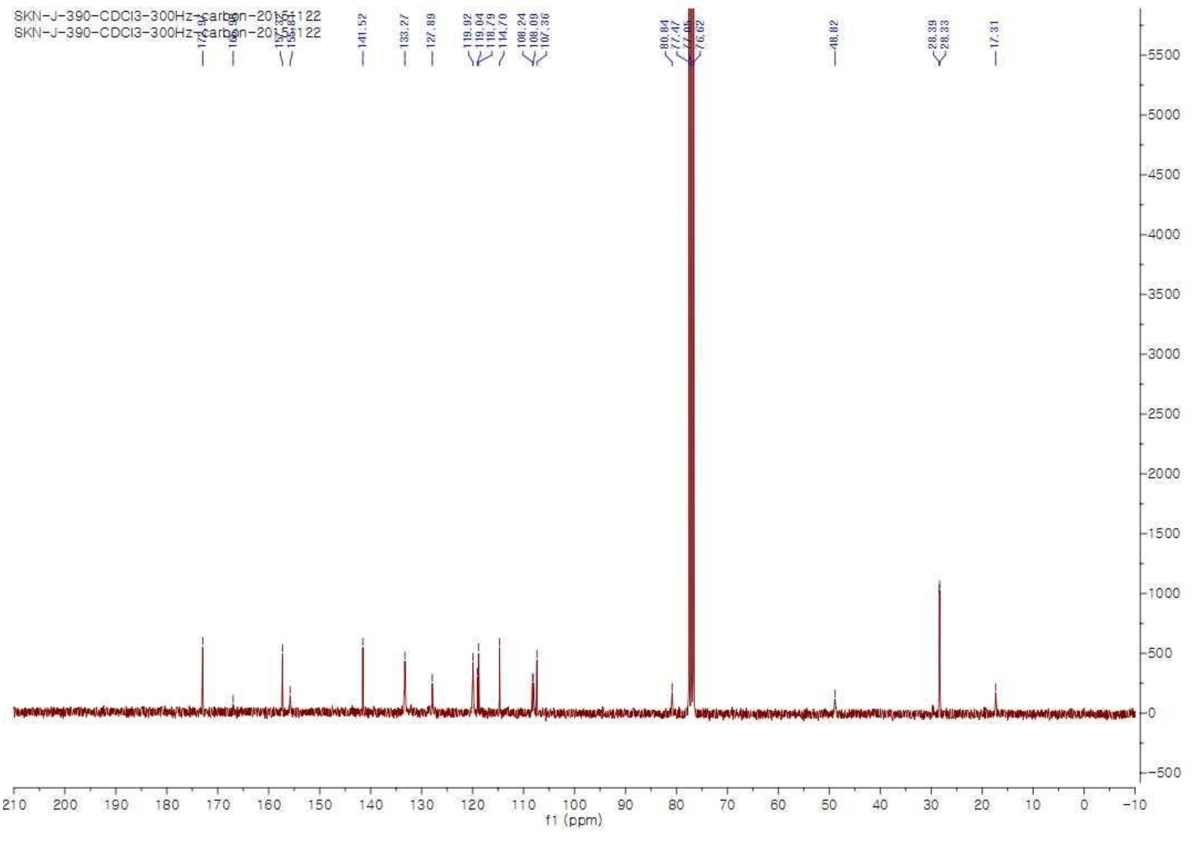

^{1}H and ^{13}C NMR of compound **11f**

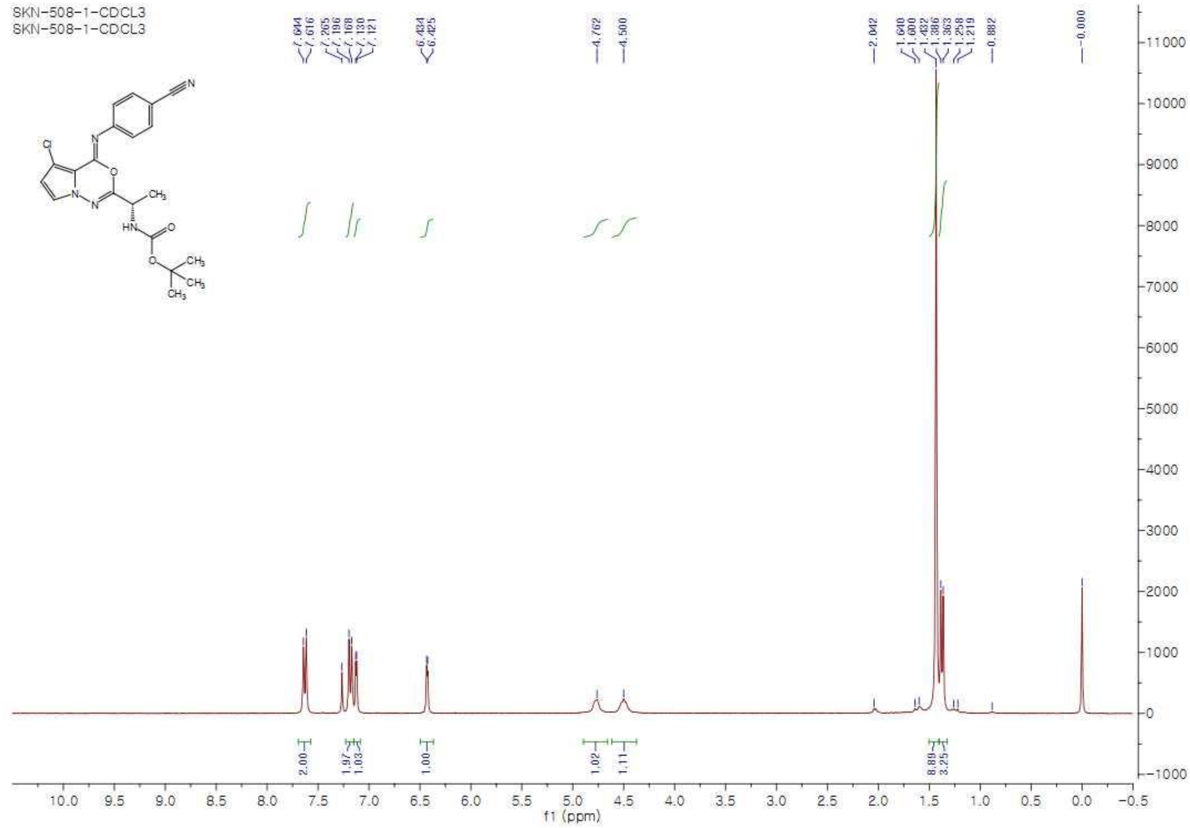

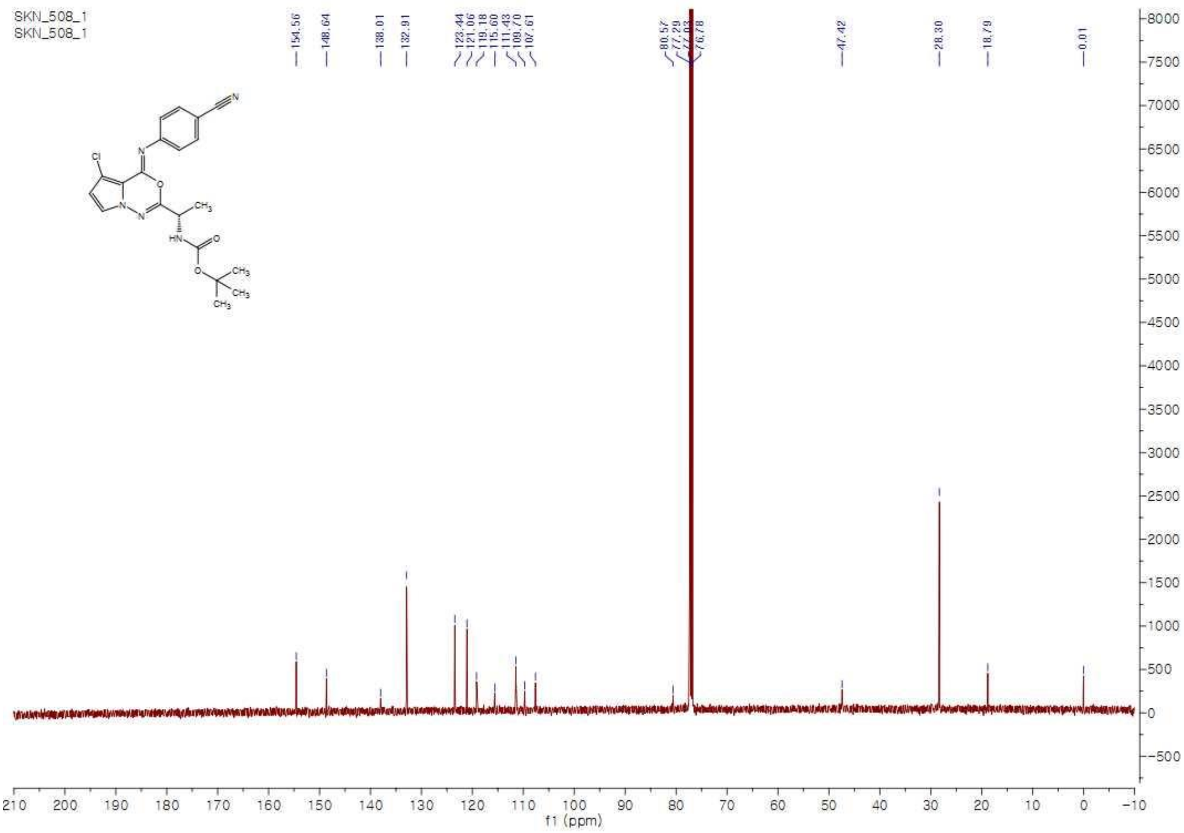

^{1}H and ^{13}C NMR of compound **12f**

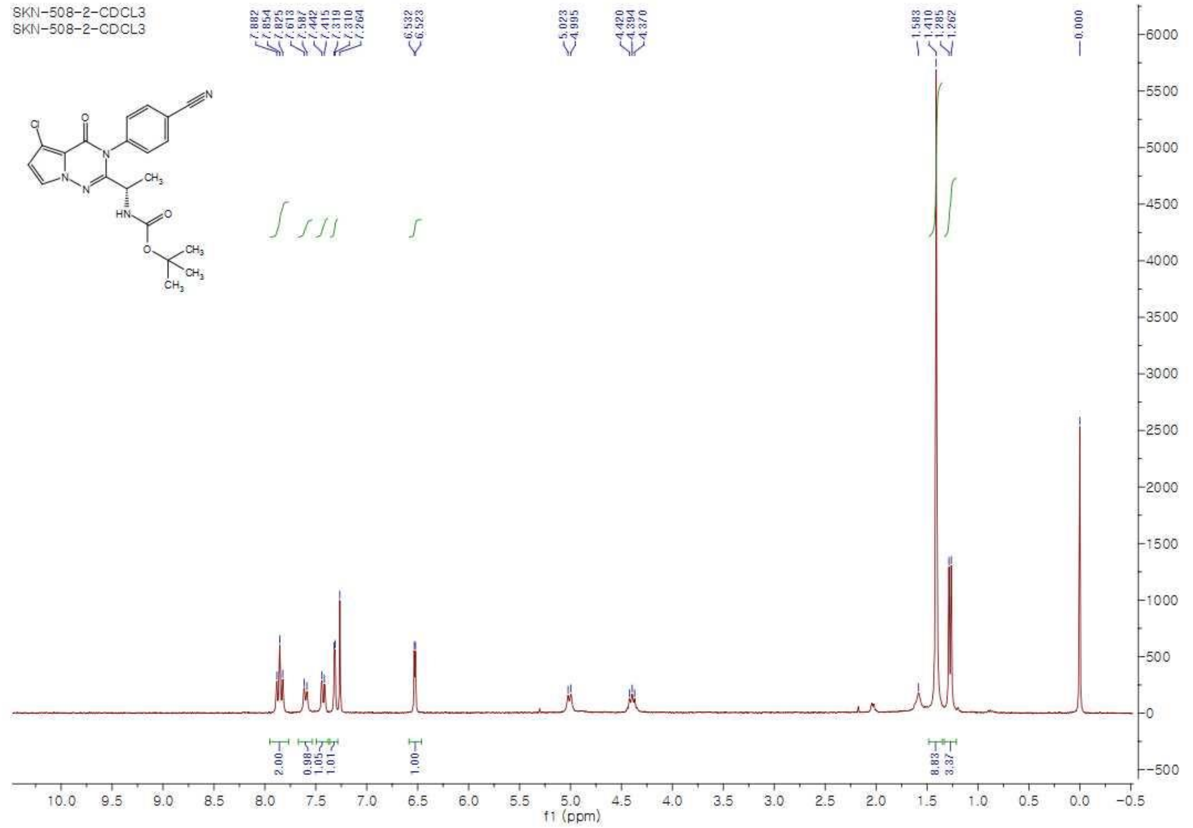

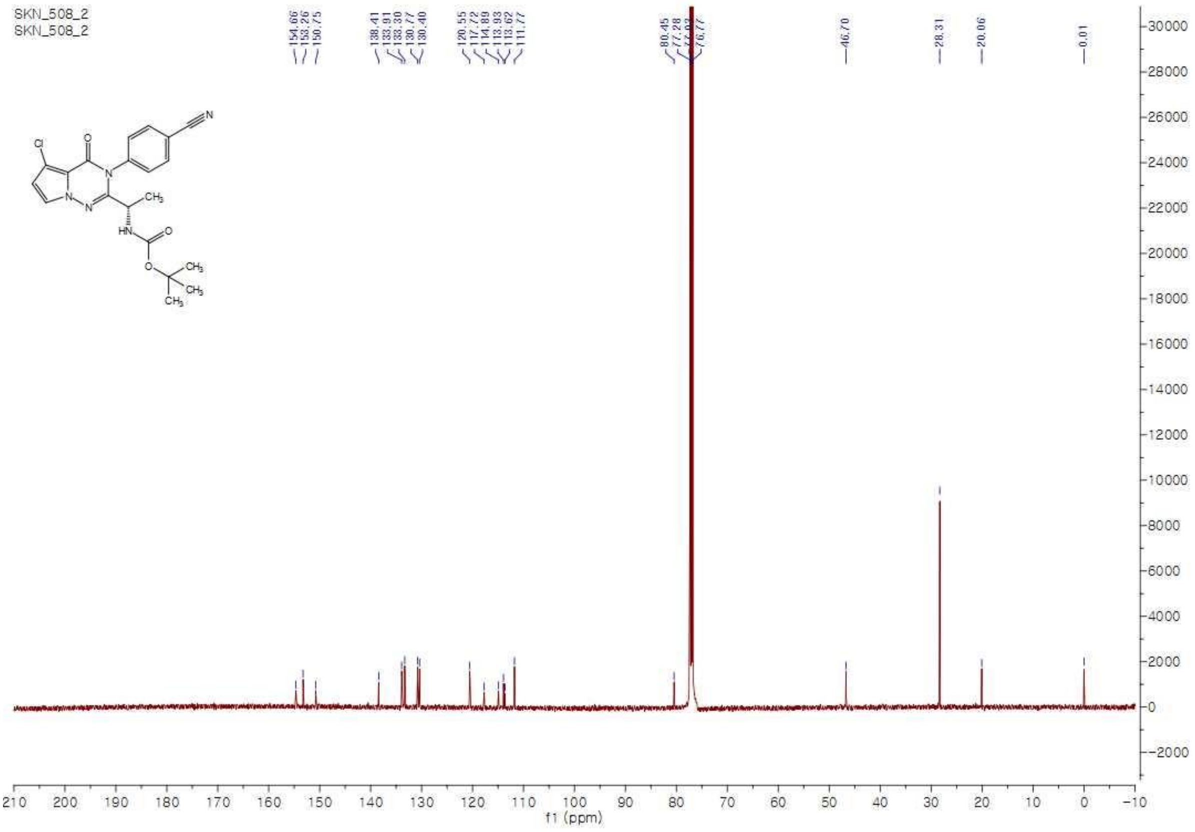

^{1}H and ^{13}C NMR of compound **14g**

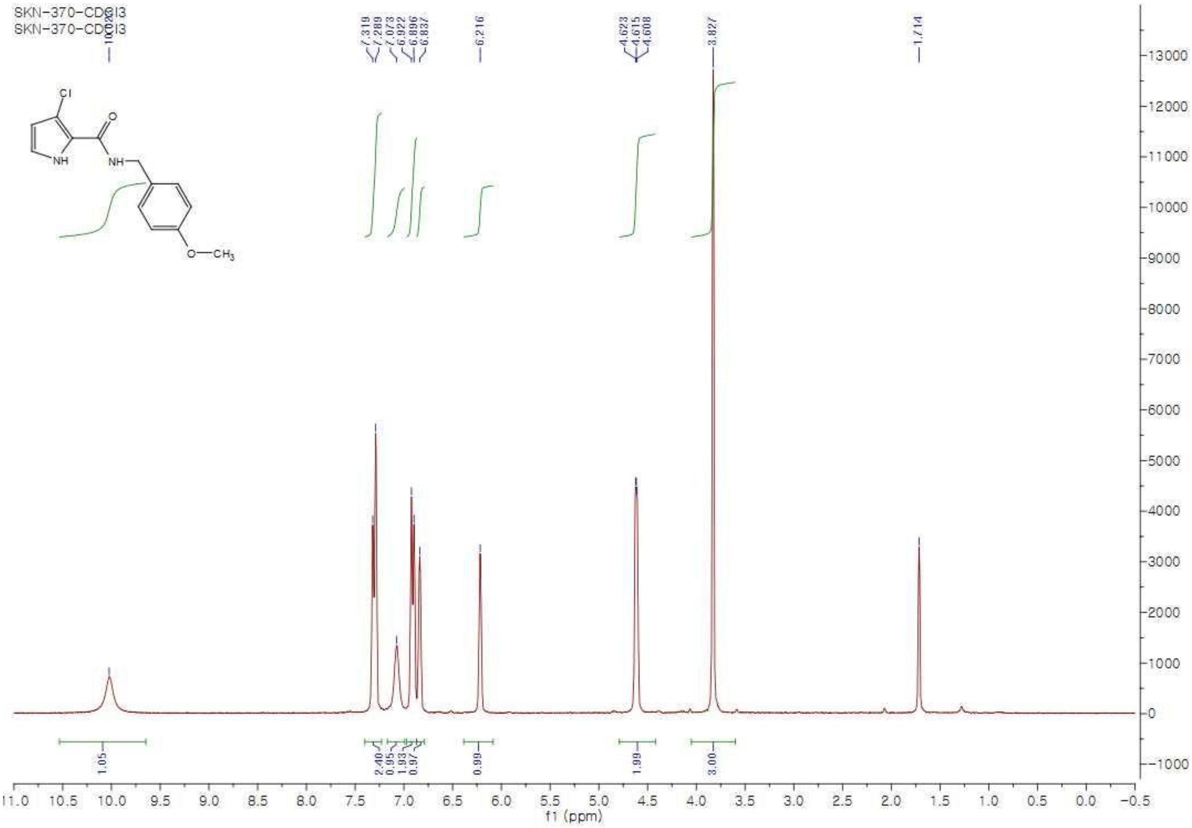

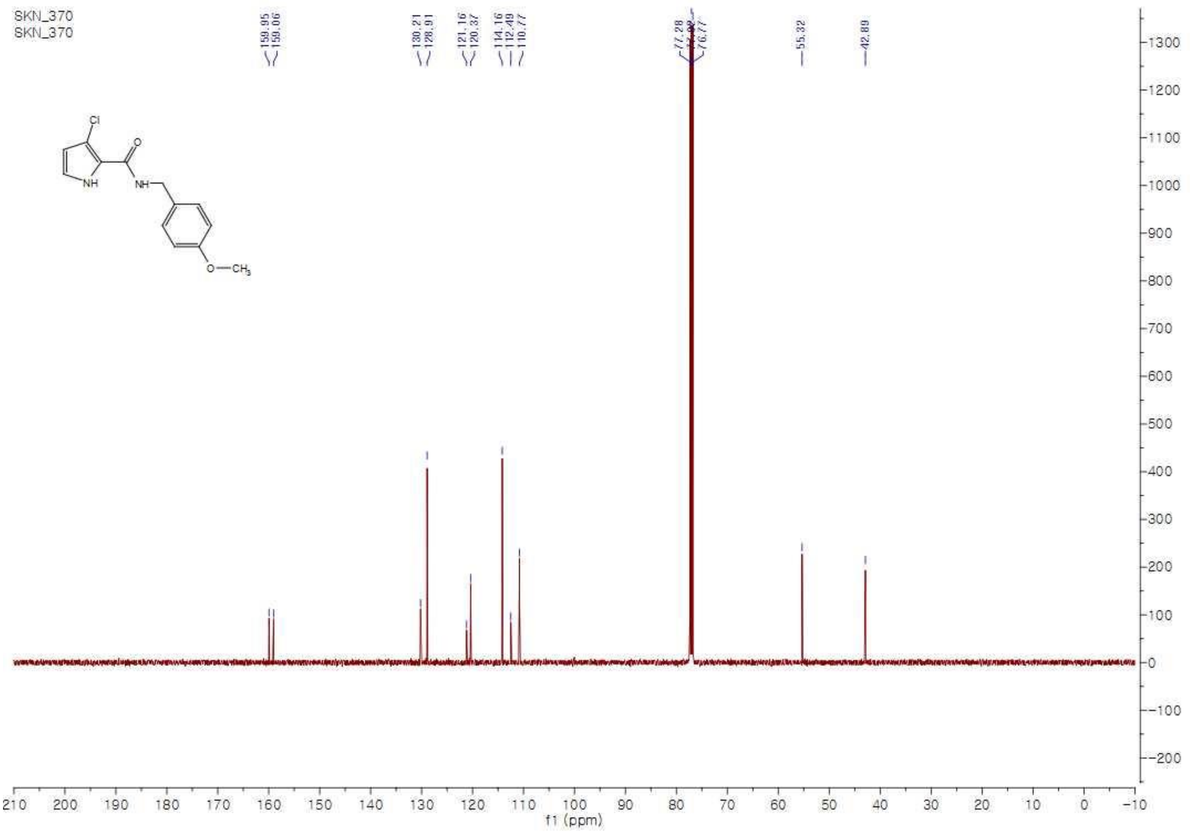

^{1}H and ^{13}C NMR of compound **15g**

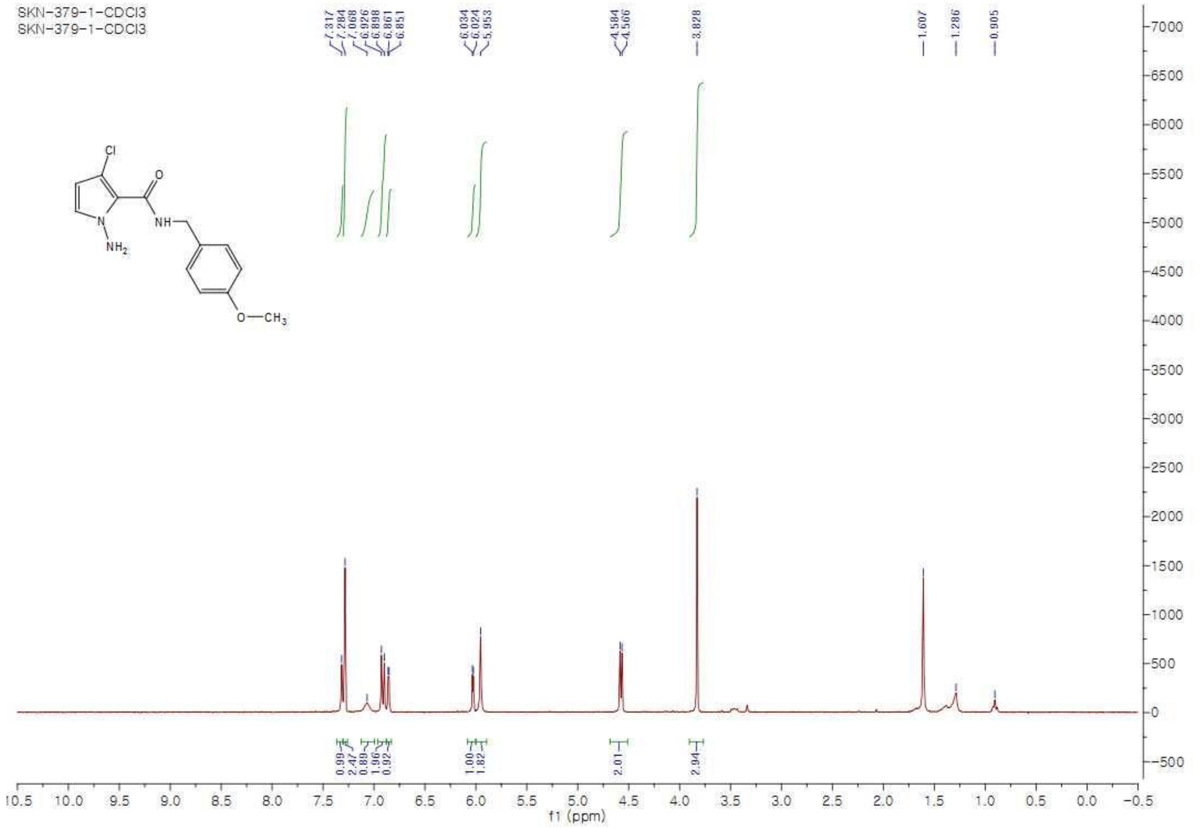

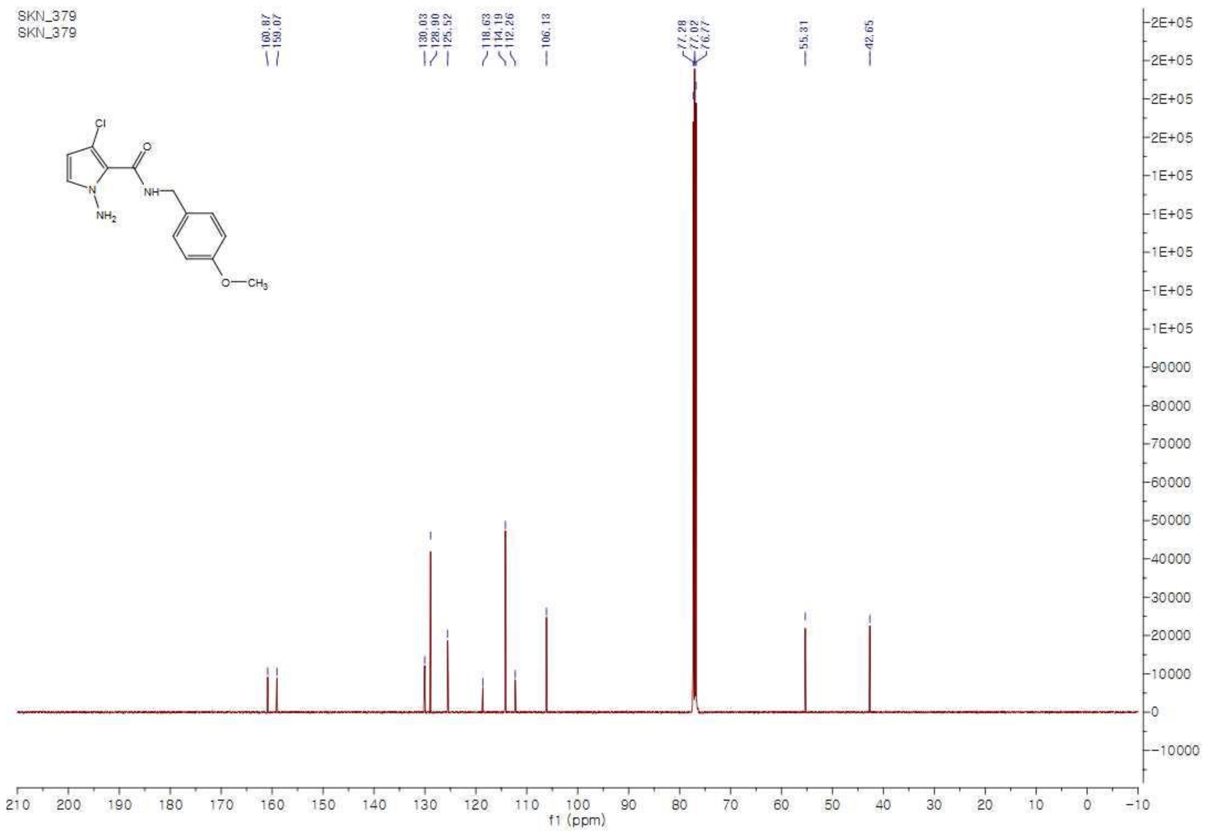

^{1}H and ^{13}C NMR of compound **10g**

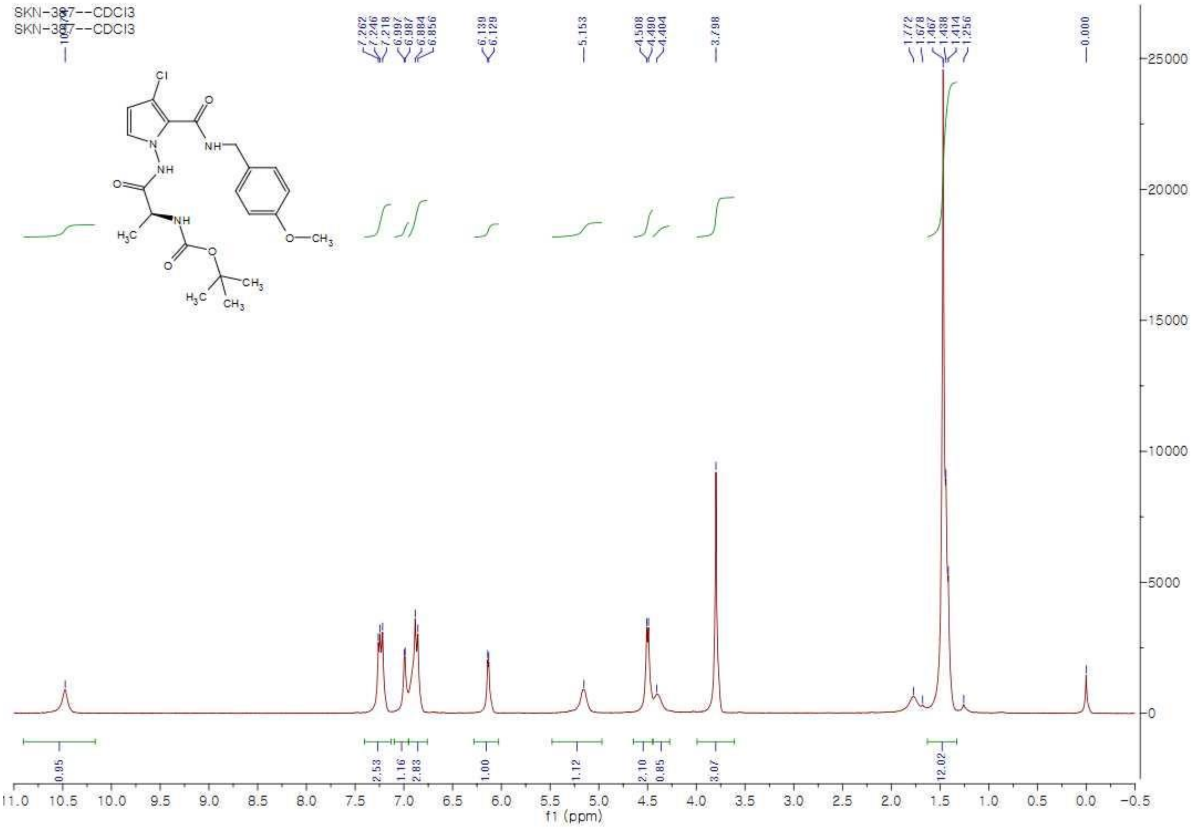

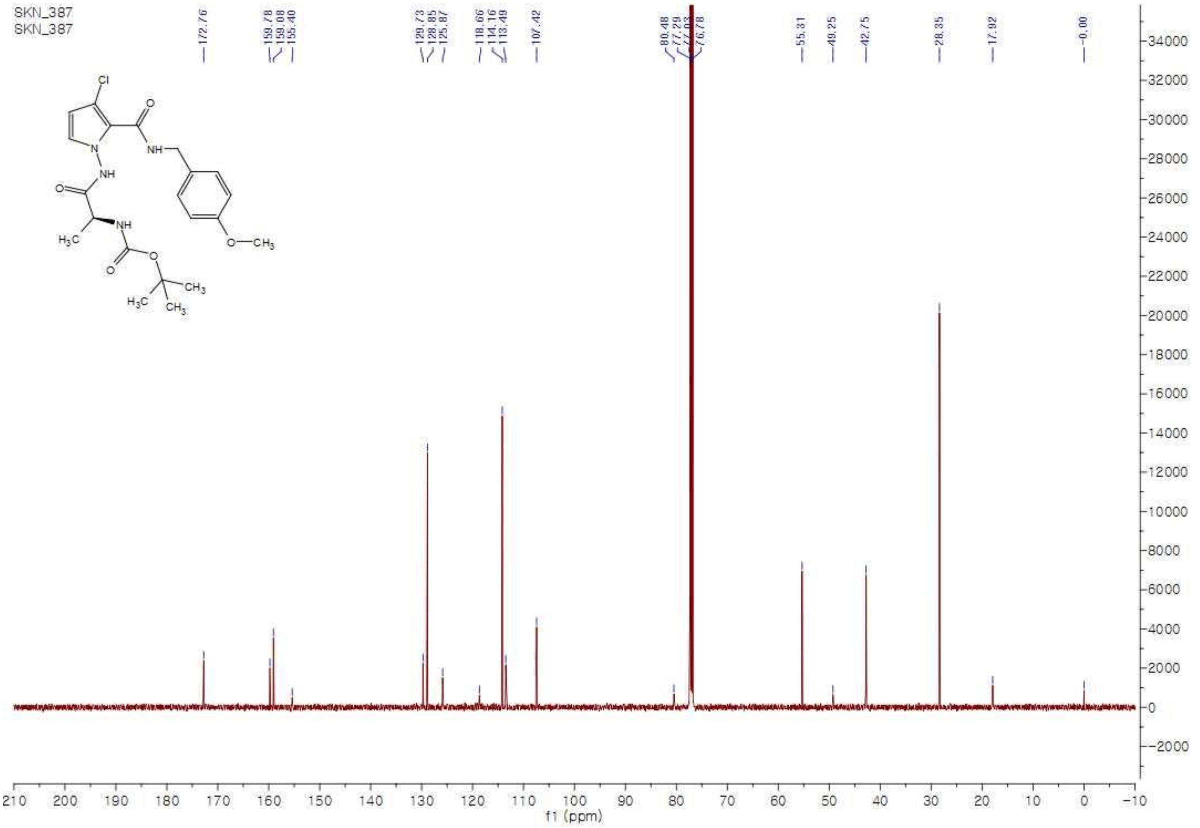

^{1}H and ^{13}C NMR of compound **12g**

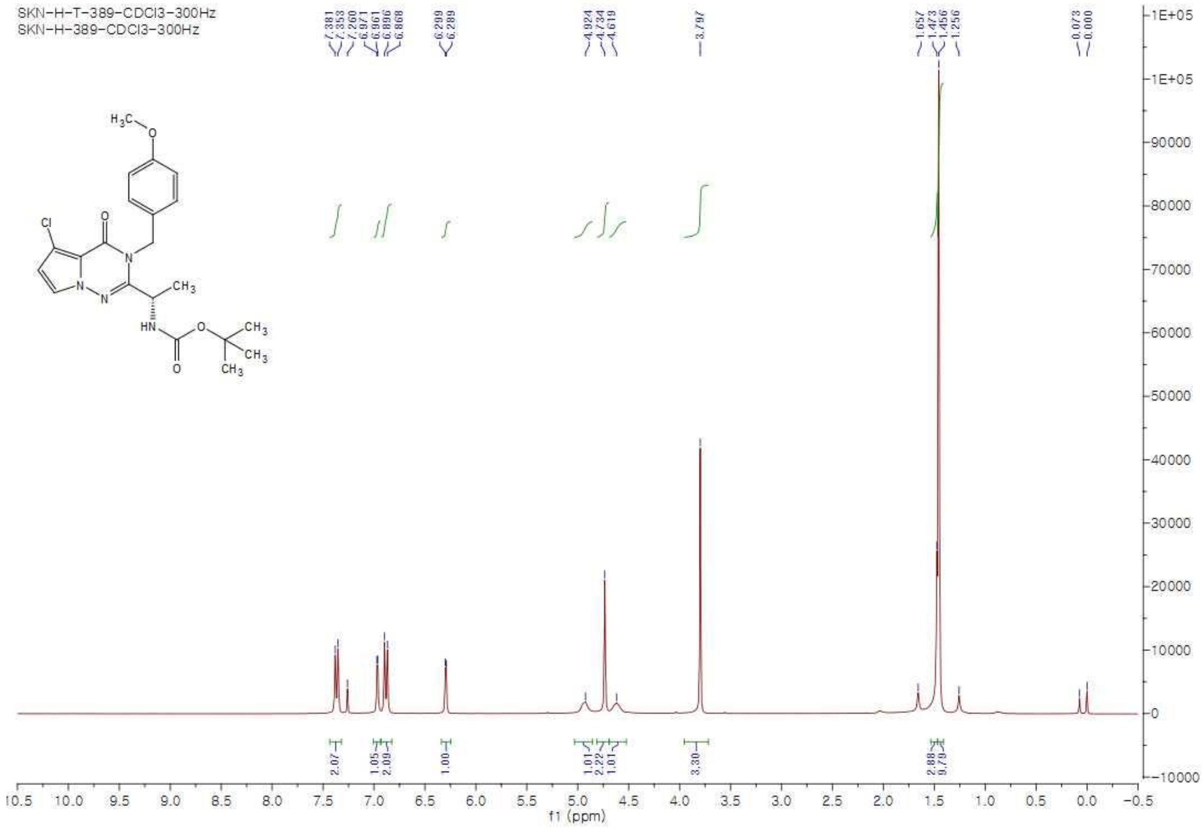

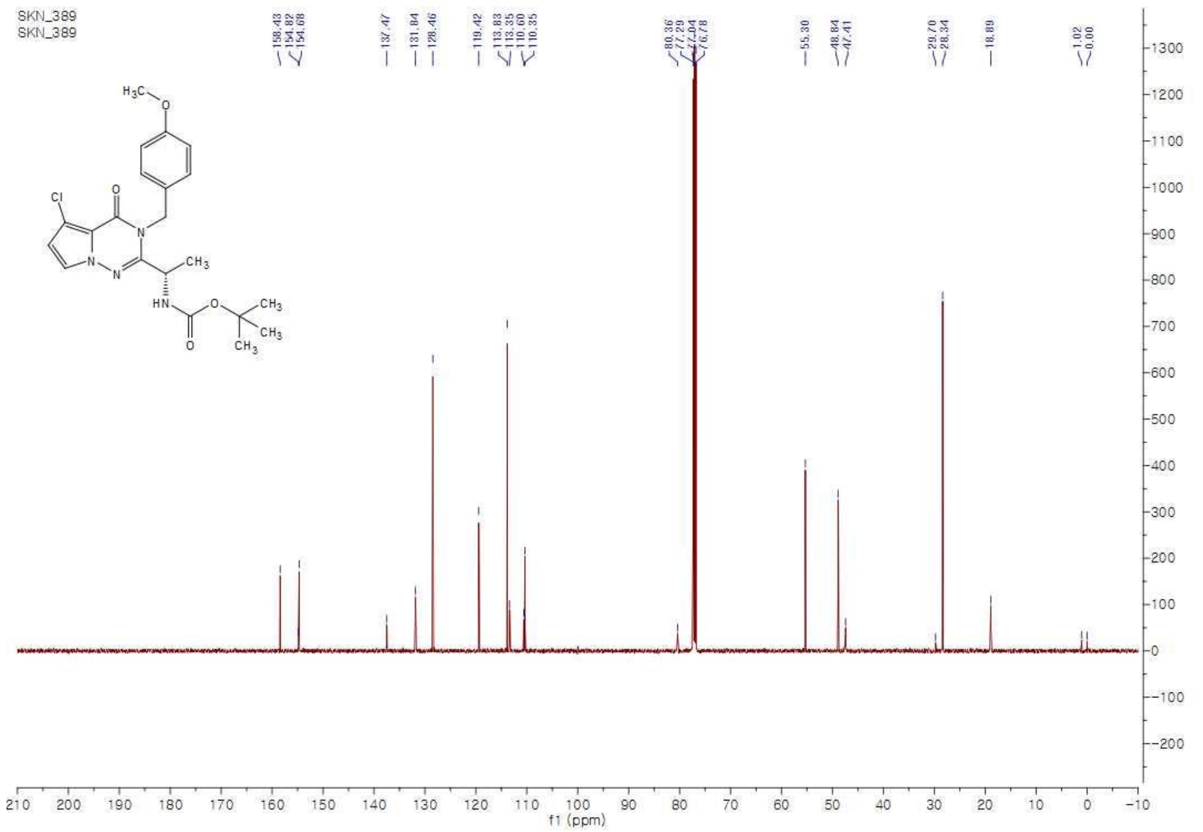

^{1}H and ^{13}C NMR of compound **14h**

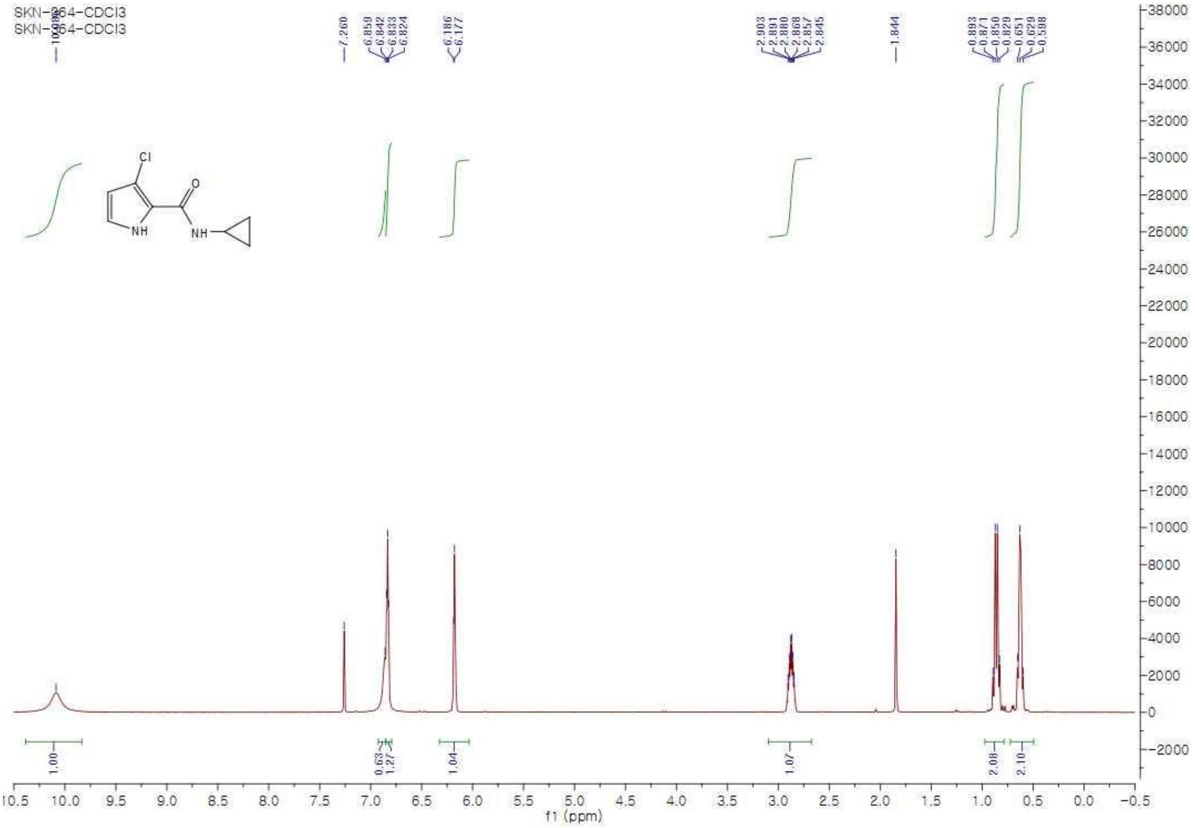

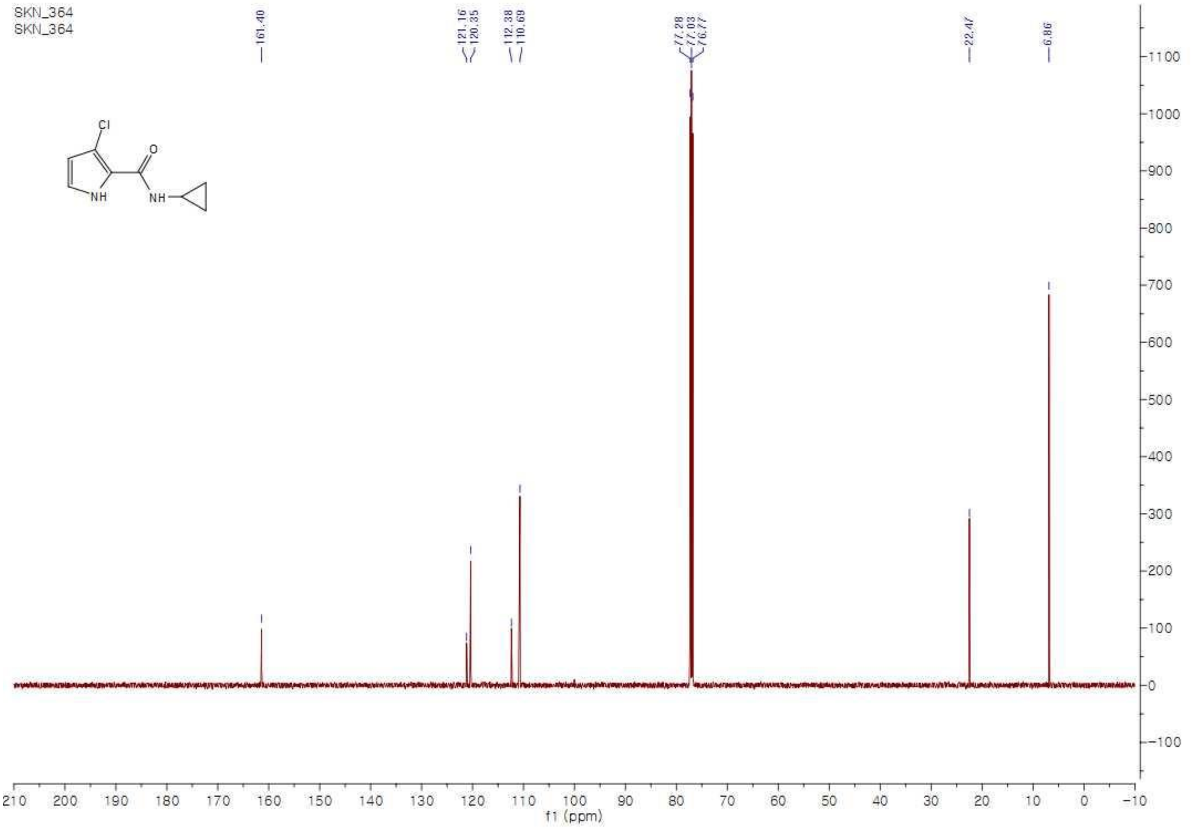

^{1}H and ^{13}C NMR of compound **15h**

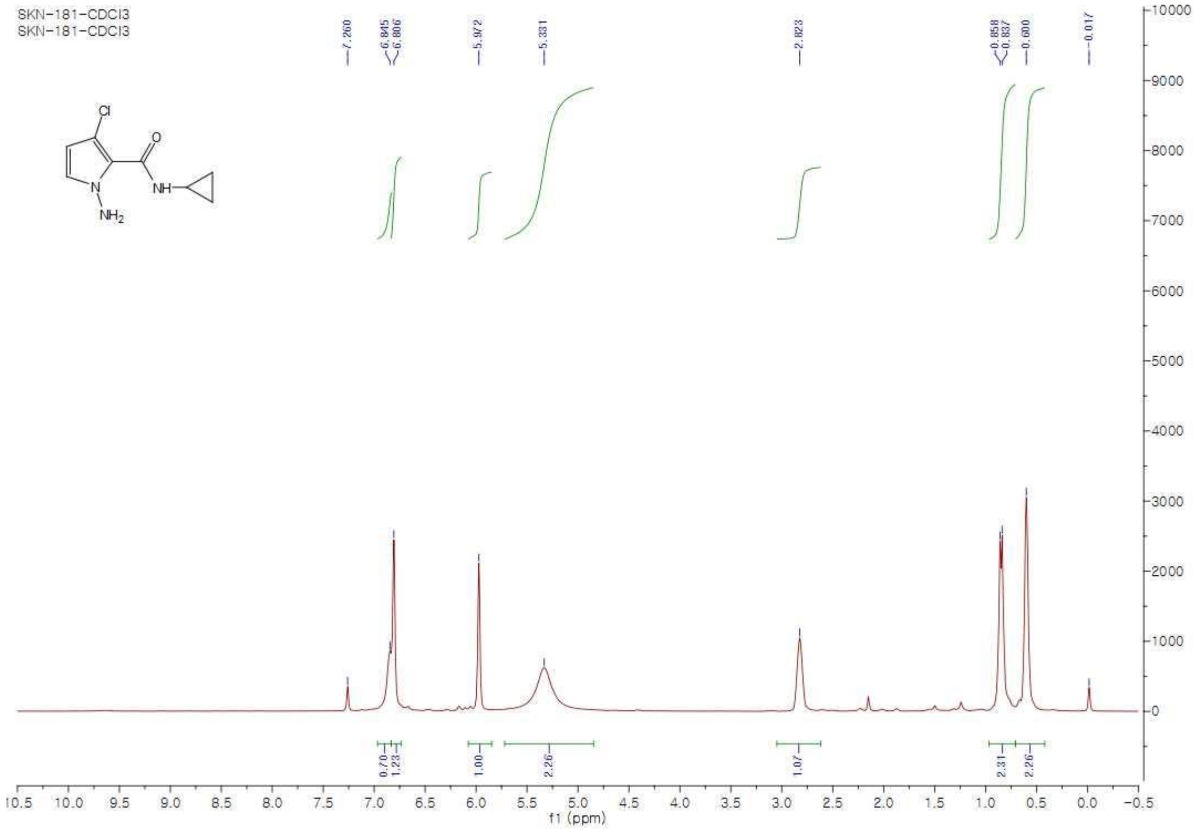

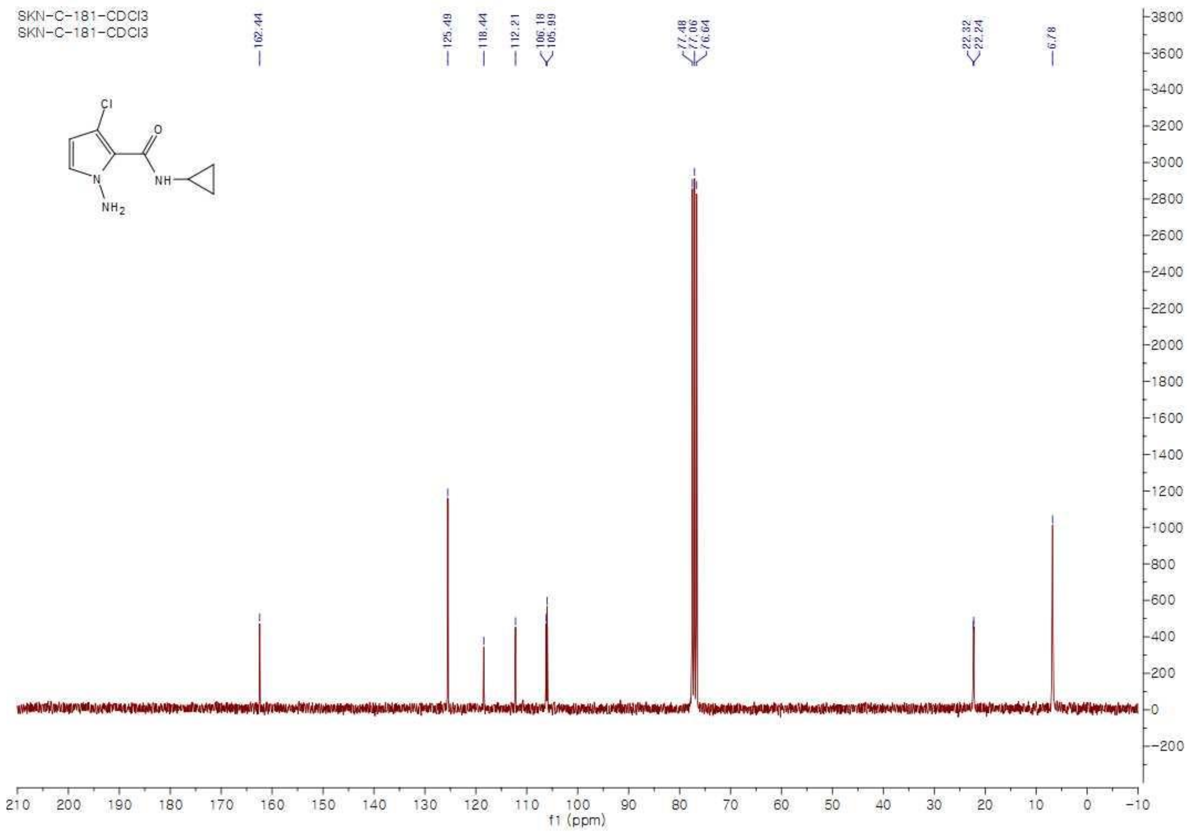

^{1}H and ^{13}C NMR of compound **10h**

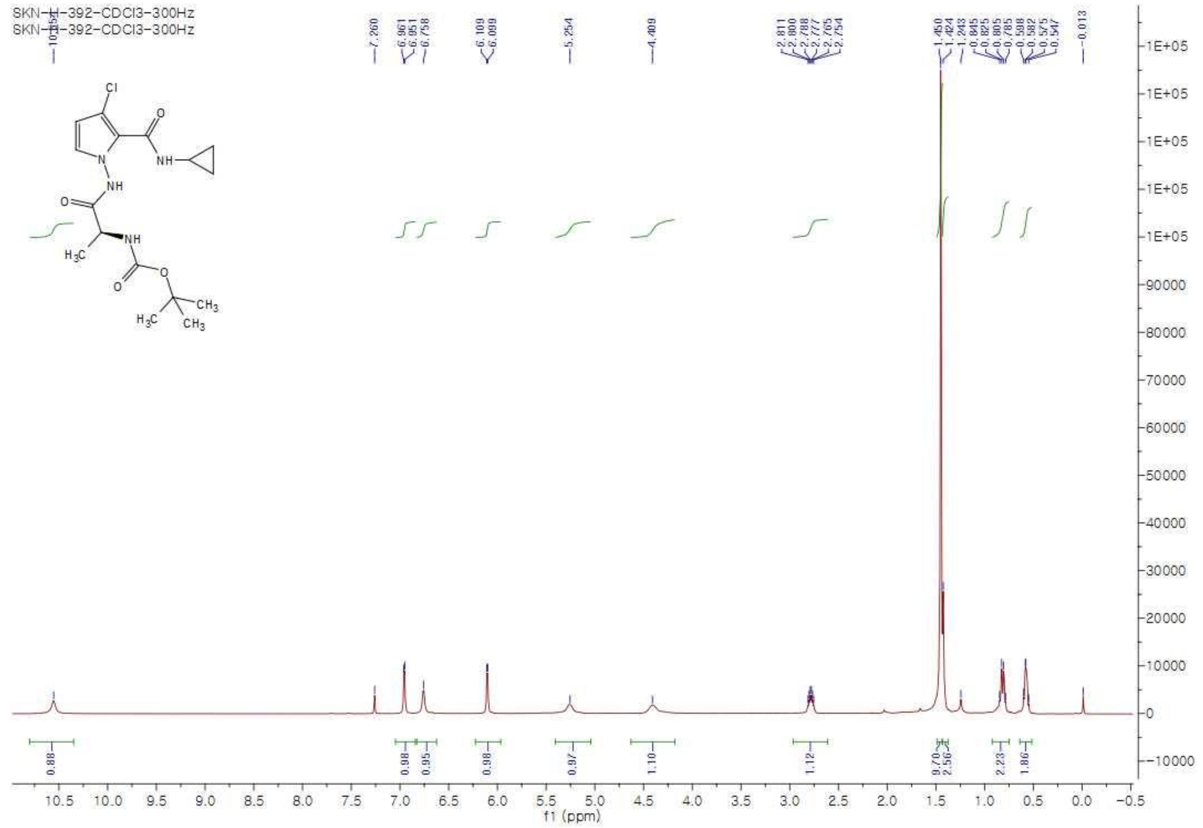

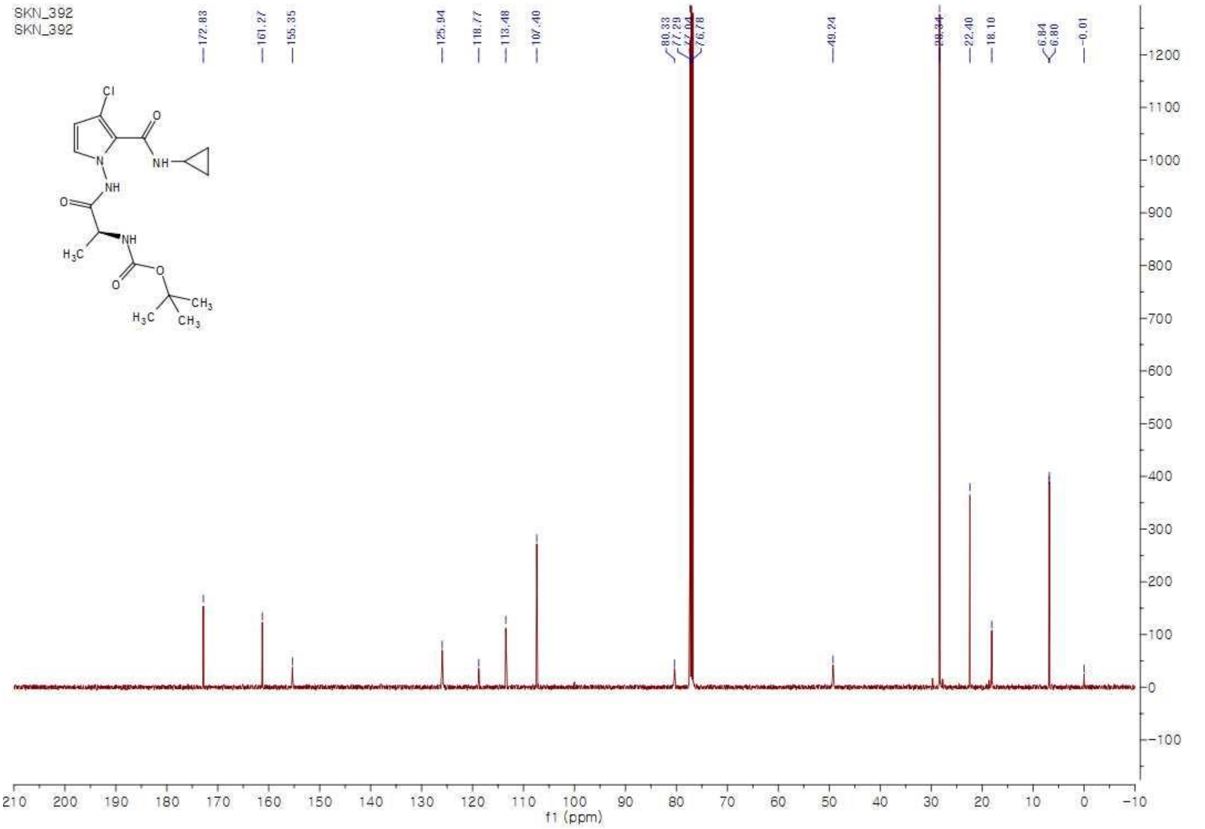

^{1}H and ^{13}C NMR of compound **12h**

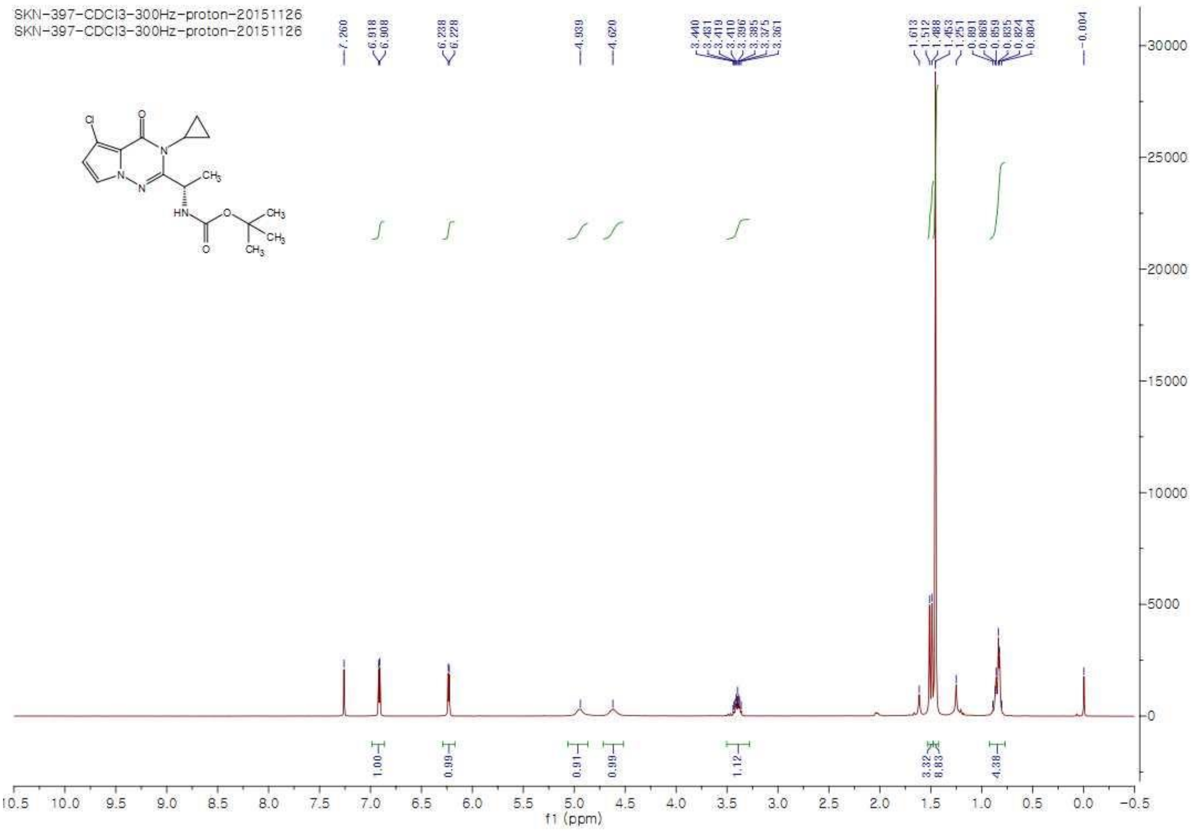

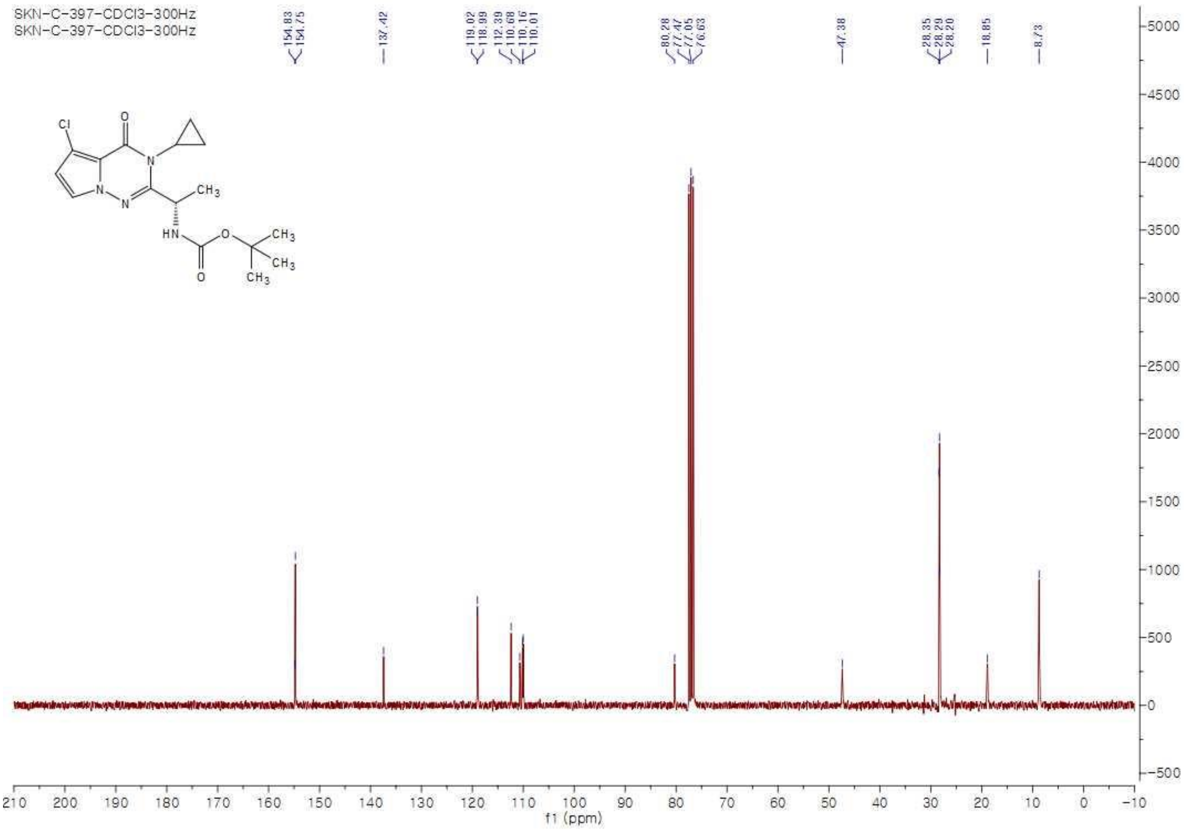

NOE of **11a** and **12a**

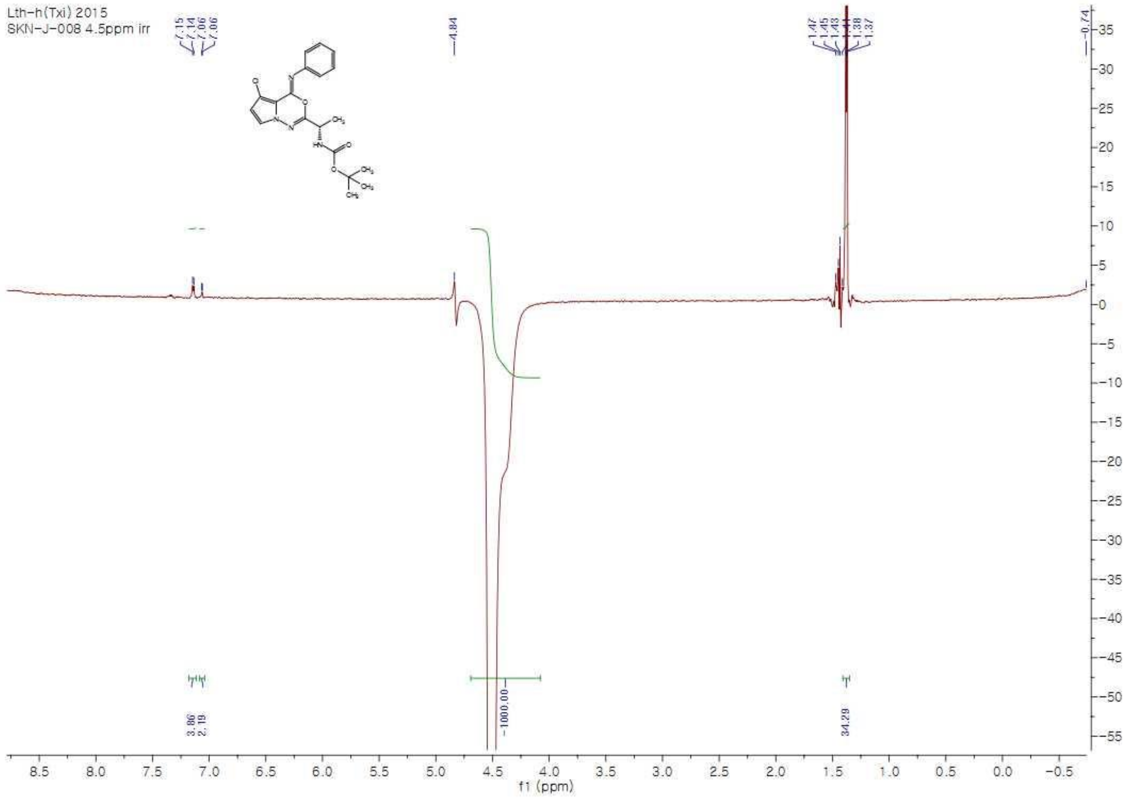

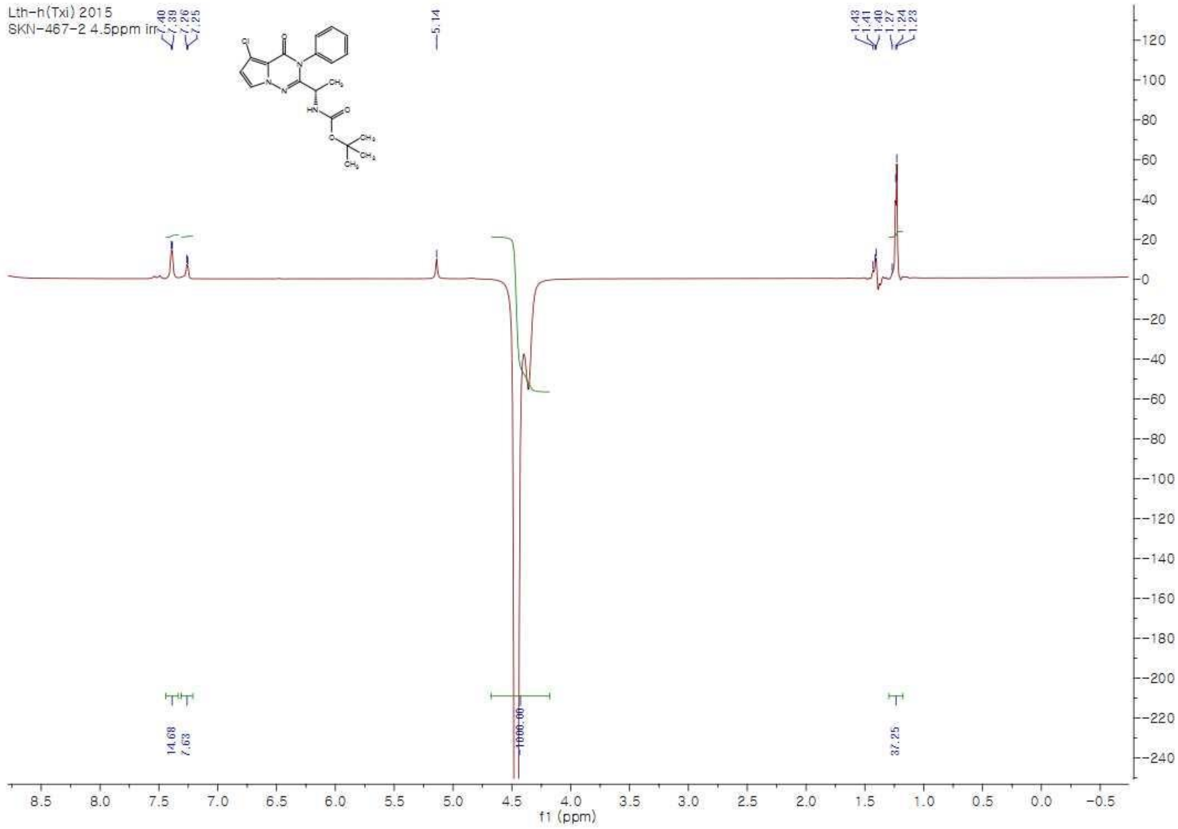

NOE of **11d** and **12d**

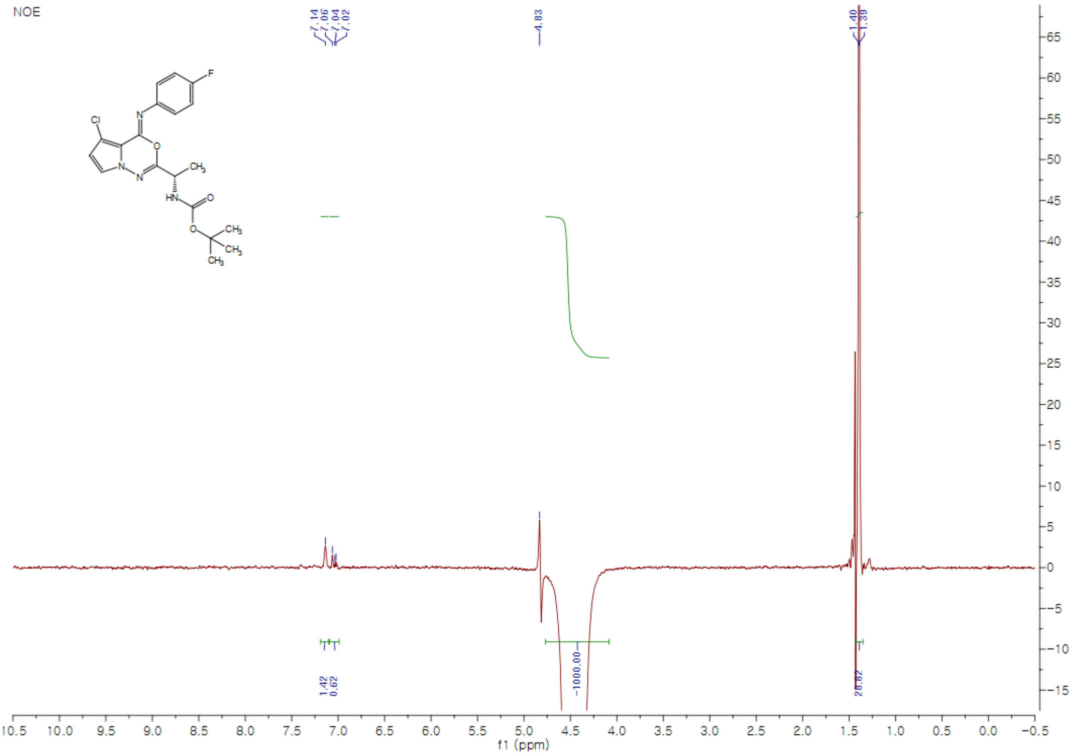

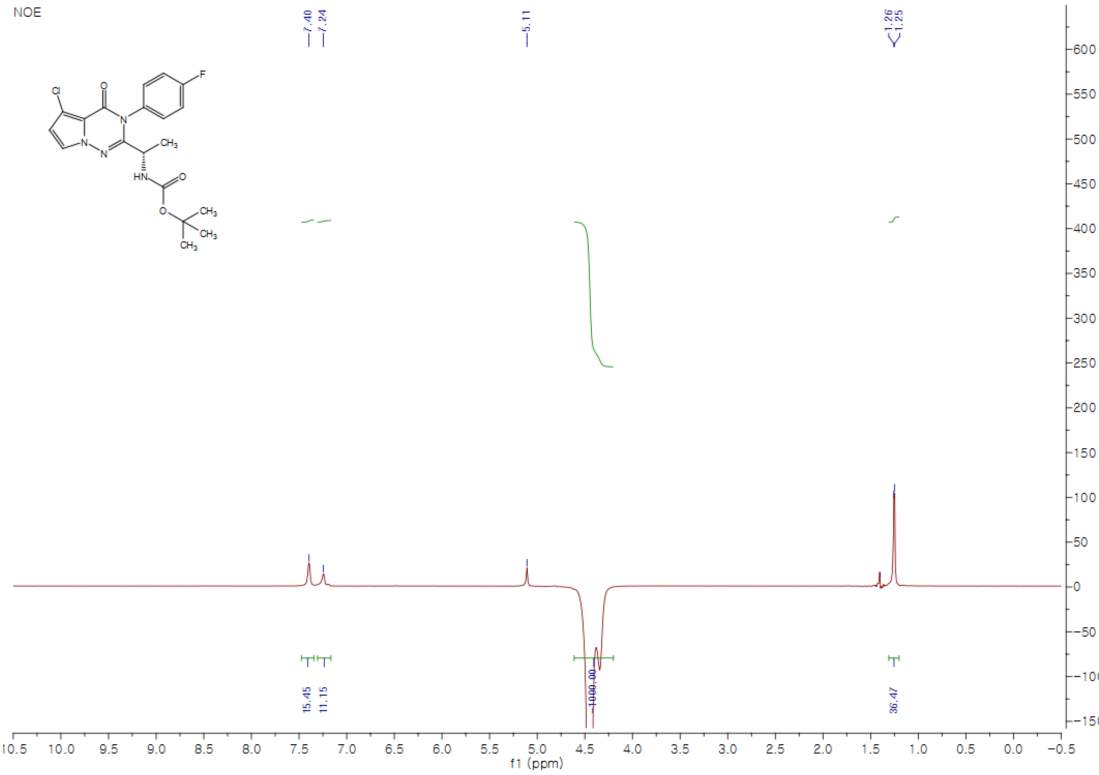

With Mosher's acid for confirming enantiomeric excess (ee) value

(OCH$_3$ peak was used for calculations)

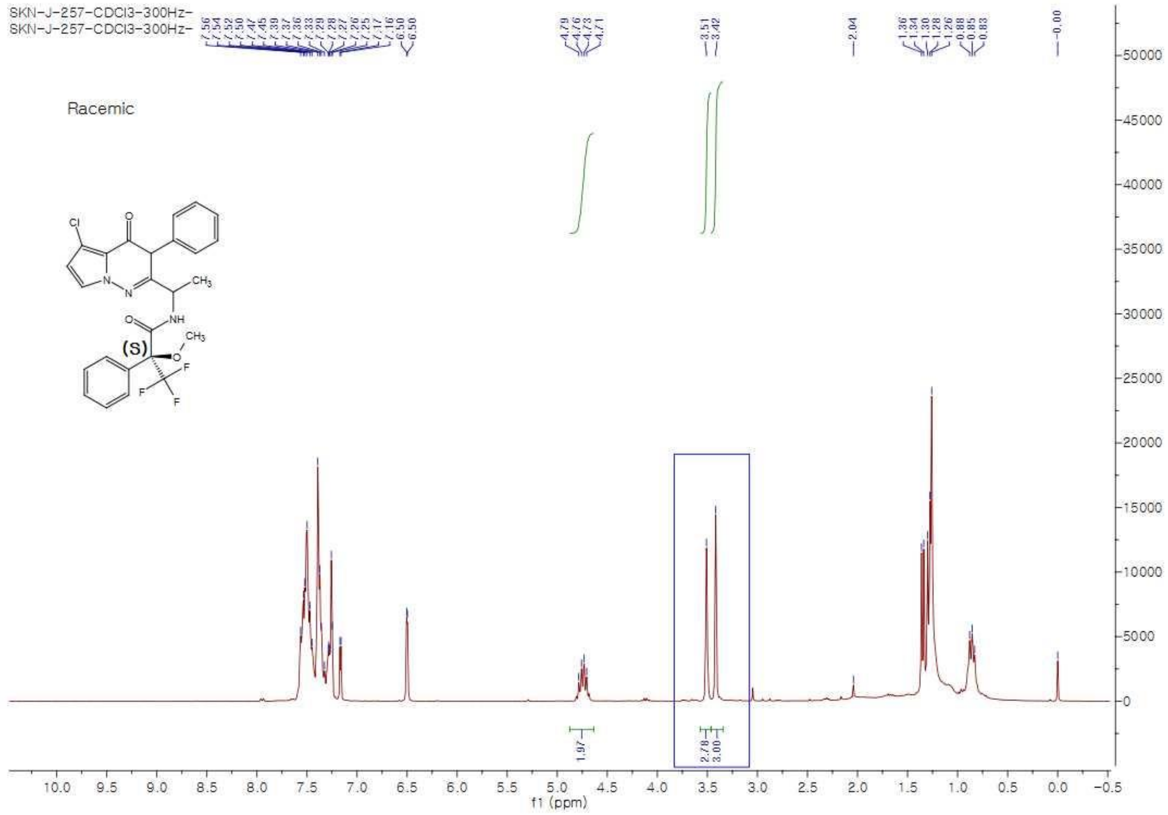

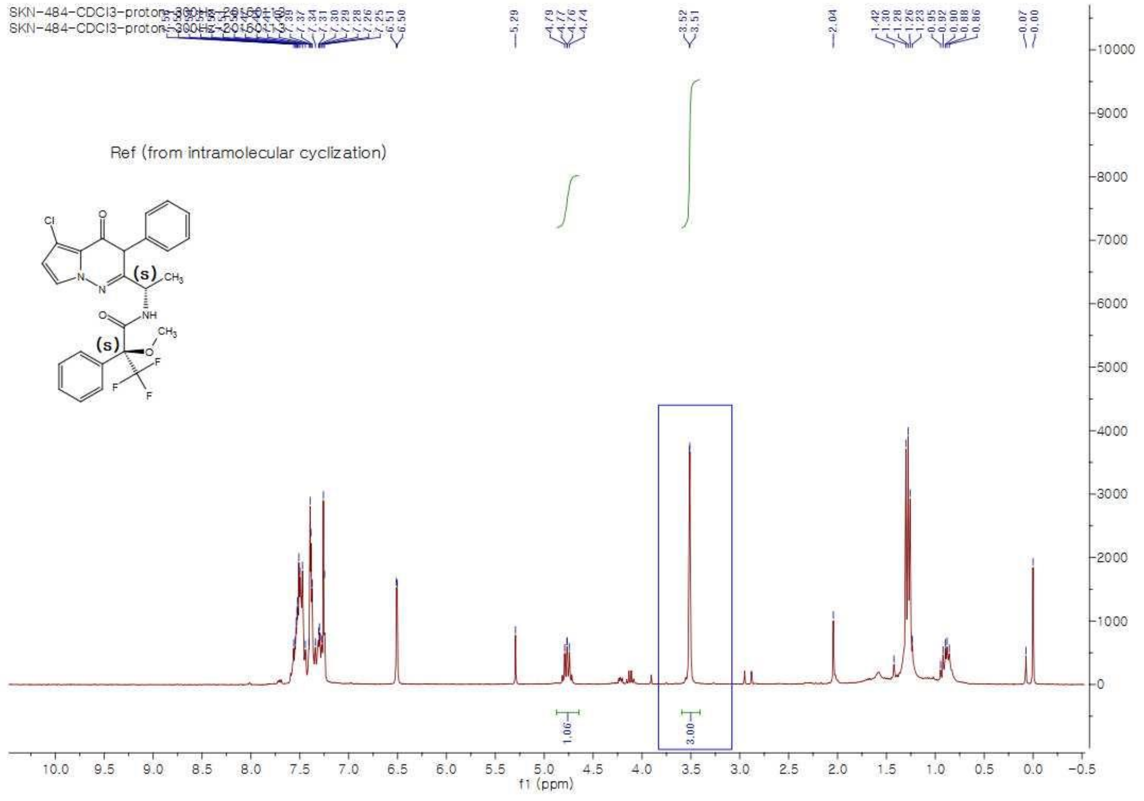

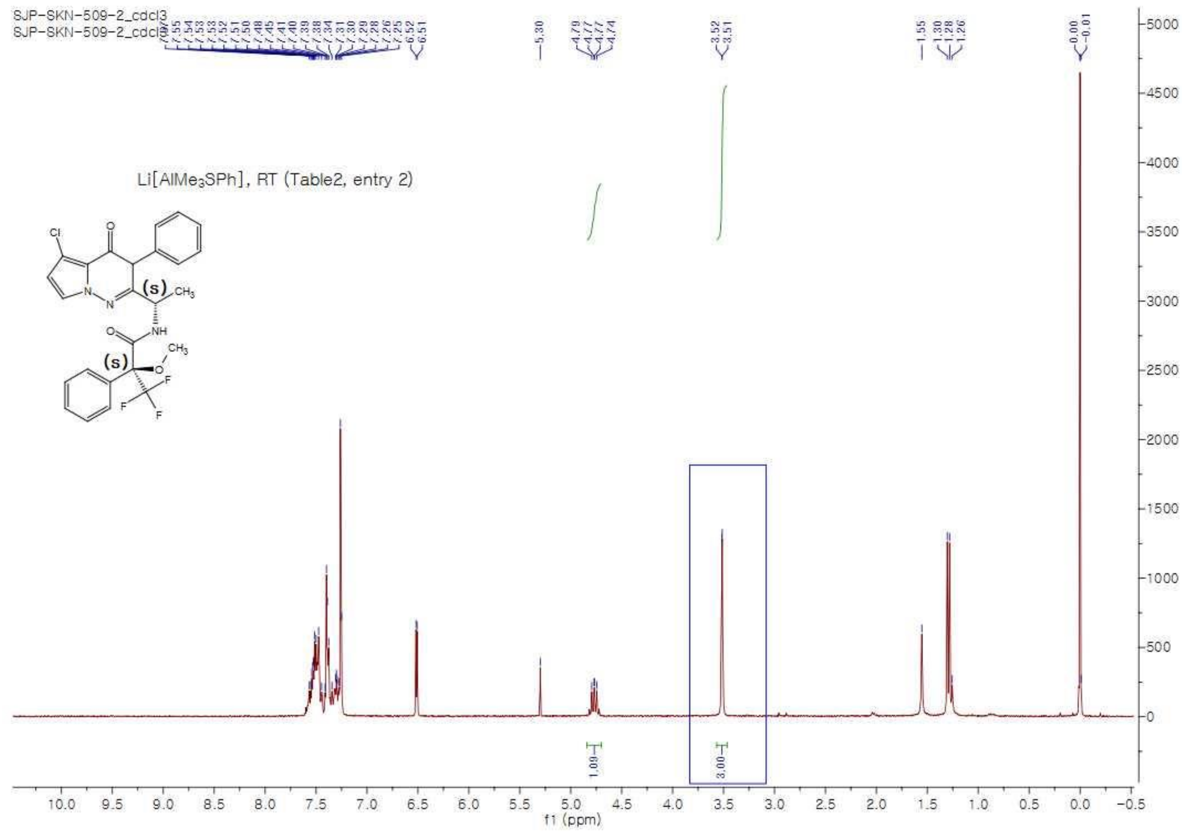
SJP-SKN-509-2_cdcl3
Li[AlMe3SPh], RT (Table2, entry 2)
Cl
O
(s)
CH3
N
NH
(s)
F
f1 (ppm)

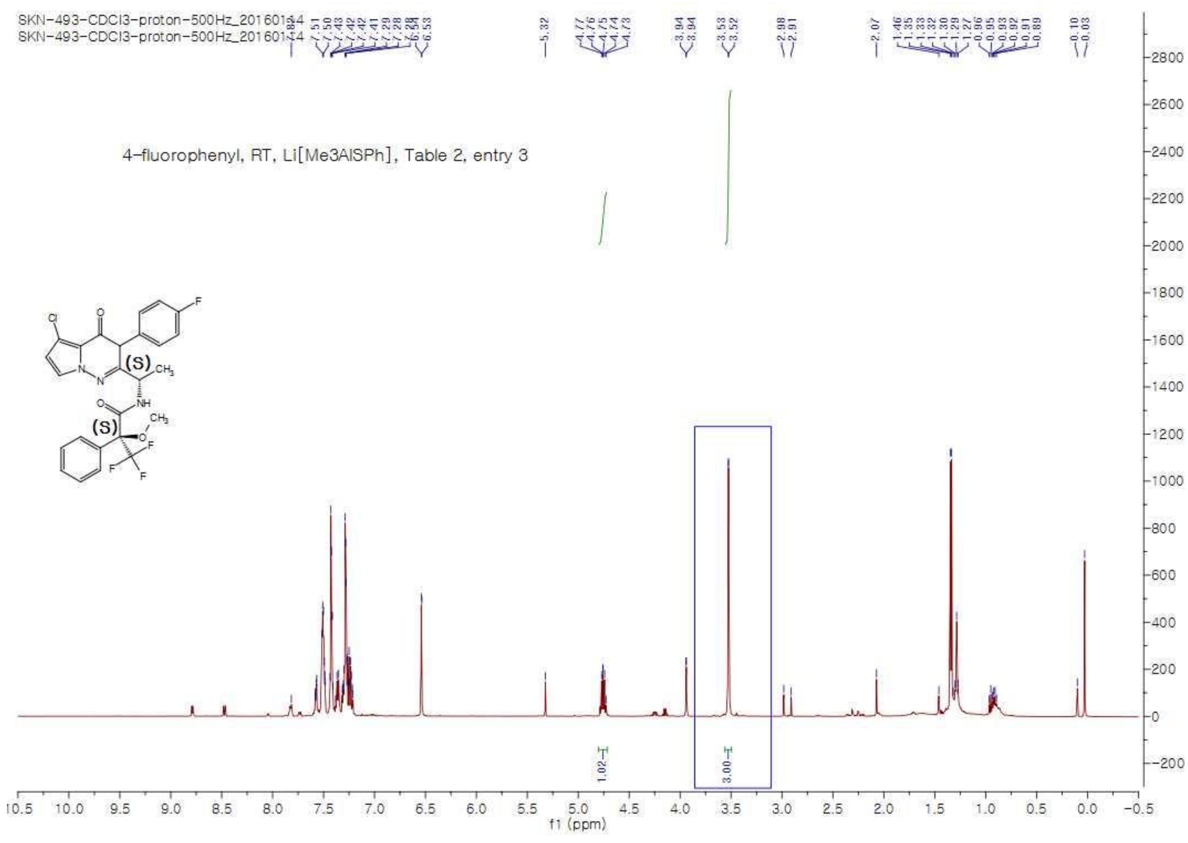
4-fluorophenyl, RT, Li[Me3AlSPh], Table 2, entry 3
Cl
O
F
(S)
CH3
N
NH
(S)
f1 (ppm)

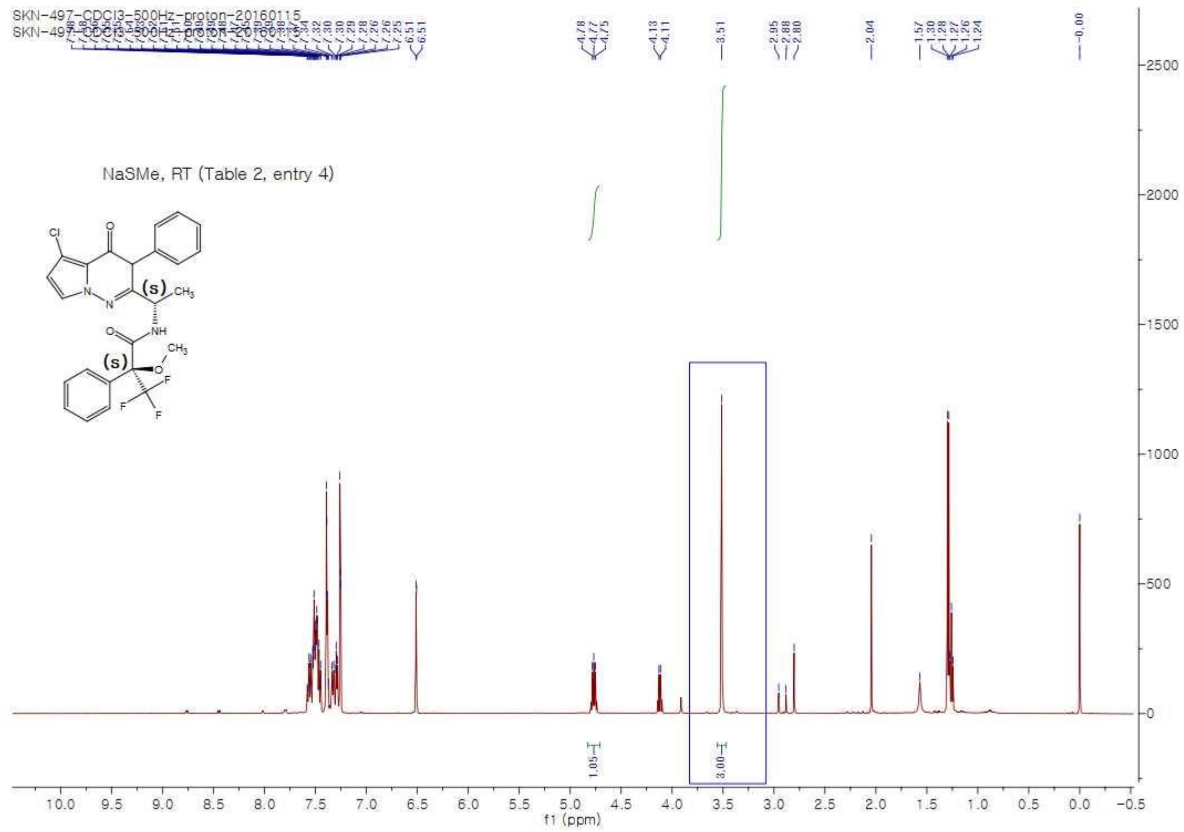
SKN-497-CDCl3-500Hz-proton-20160115
NaSMe, RT (Table 2, entry 4)
f1 (ppm)

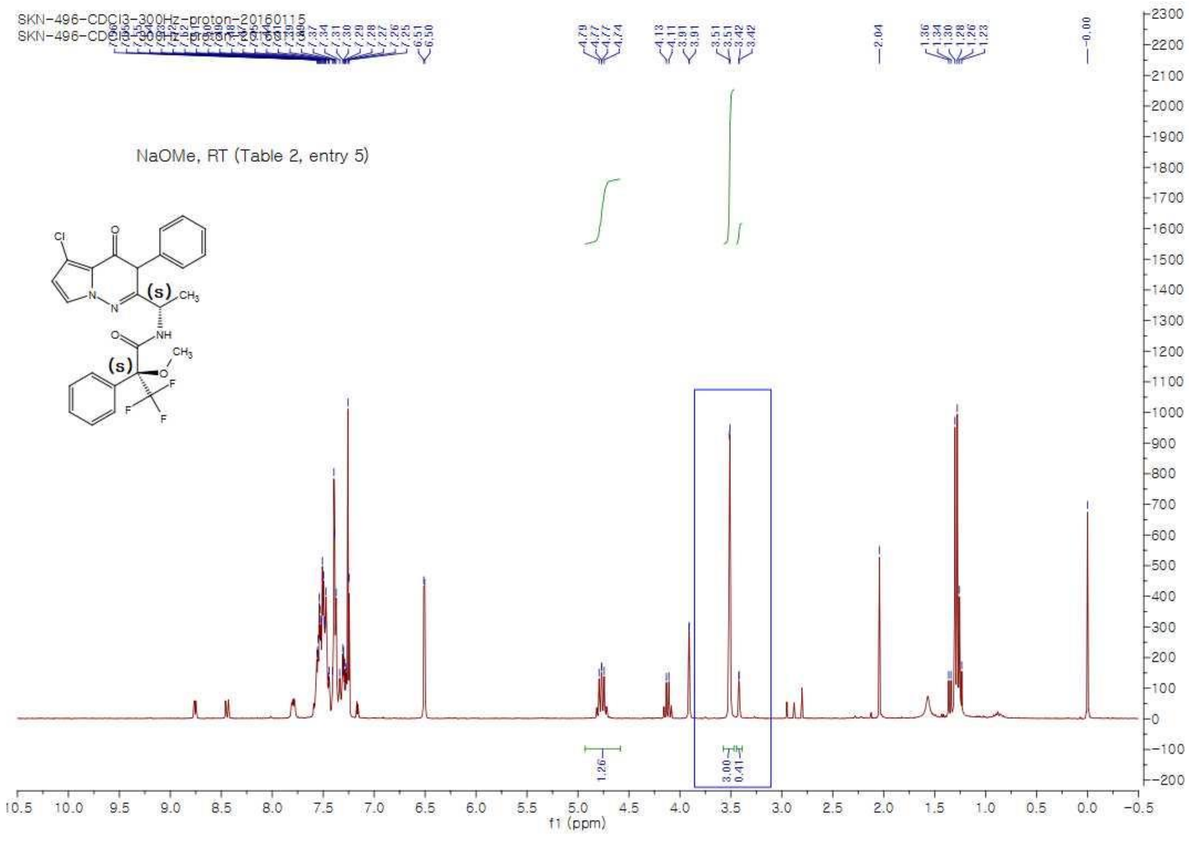
SKN-496-CDCl3-300Hz-proton-20160115
NaOMe, RT (Table 2, entry 5)
f1 (ppm)

Different peak patterns in the NH protons (^{1}H NMR spectra, representative examples, **11a**/**11d** and **12a**/**12d**)

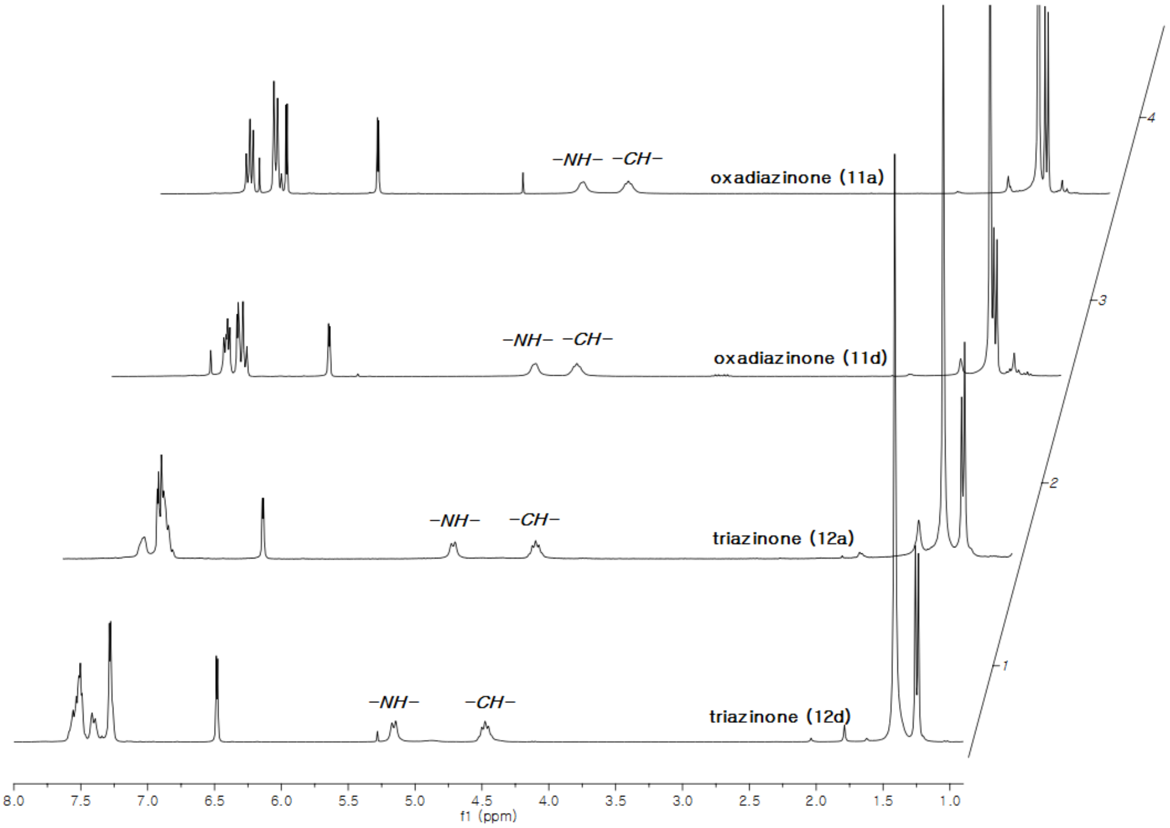

Supplement: File 1 — Experimental and analytical data. [file Beilstein_J_Org_Chem-12-1780-s001.pdf]
